# A strategy based on nucleotide specificity leads to a subfamily-selective and cell-active inhibitor of *N* ^6^-methyladenosine demethylase FTO†
†Electronic supplementary information (ESI) available: Experimental details, including full synthesis procedure, *T*_m_ shift analyses, biochemical and cell-based assay conditions, protein purification methods, crystallisation and structure solution methods. The coordinates and structural factors for FTO in complex with **12**, **16** and **21** have been deposited in the RCSB Protein Data Bank as PDB ID 4CXW, 4CXX and 4CXY. See DOI: 10.1039/c4sc02554g


**DOI:** 10.1039/c4sc02554g

**Published:** 2014-09-22

**Authors:** Joel D. W. Toh, Lingyi Sun, Lisa Z. M. Lau, Jackie Tan, Joanne J. A. Low, Colin W. Q. Tang, Eleanor J. Y. Cheong, Melissa J. H. Tan, Yun Chen, Wanjin Hong, Yong-Gui Gao, Esther C. Y. Woon

**Affiliations:** a Department of Pharmacy , National University of Singapore , 18 Science Drive 4 , 117543 , Singapore . Email: esther.woon@nus.edu.sg ; Fax: +65 6779 1554 ; Tel: +65 6516 2932; b Institute of Molecular and Cell Biology , 61 Biopolis Drive, Proteos , 138673 , Singapore; c School of Biological Sciences , Nanyang Technological University , 60 Nanyang Drive , 637551 , Singapore . Email: ygao@ntu.edu.sg ; Fax: +65 6779 1117

## Abstract

The AlkB family of nucleic acid demethylases are of intense biological and medical interest because of their roles in nucleic acid repair and epigenetic modification. However their functional and molecular mechanisms are unclear, hence, there is strong interest in developing selective inhibitors for them. Here we report the identification of key residues within the nucleotide-binding sites of the AlkB subfamilies that likely determine their substrate specificity. We further provide proof of principle that a strategy exploiting these inherent structural differences can enable selective and potent inhibition of the AlkB subfamilies. This is demonstrated by the first report of a subfamily-selective and cell-active FTO inhibitor **12**. The distinct selectivity of **12** for FTO against other AlkB subfamilies and 2OG oxygenases shall be of considerable interest with regards to its potential use as a functional probe. The strategy outlined here is likely applicable to other AlkB subfamilies, and, more widely, to other 2OG oxygenases.

## Abstract 2

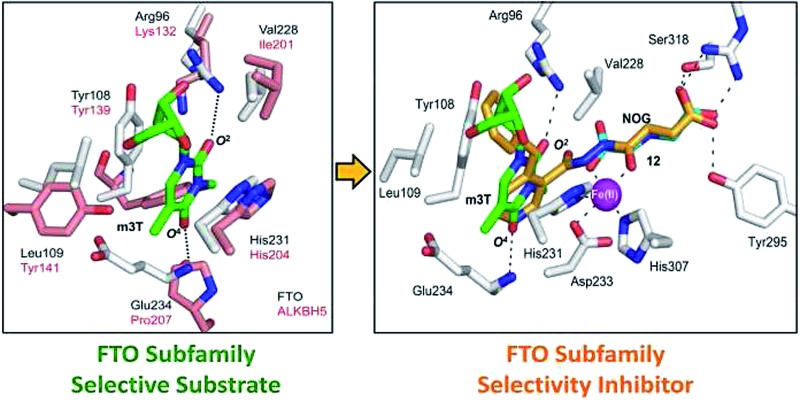
The AlkB family of nucleic acid demethylases are of intense biological and medical interest. The discovery of a highly selective FTO inhibitor should greatly facilitate the study of these enzymes.

## Introduction

**Fig. 1:**
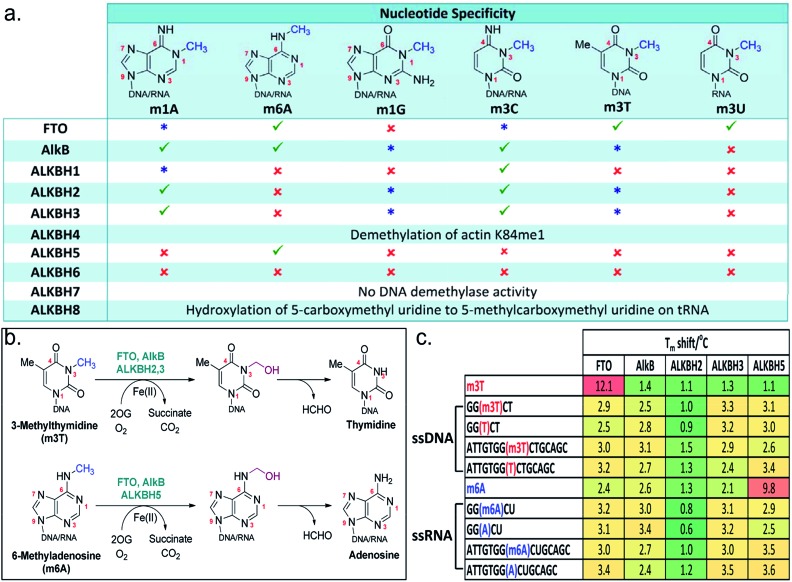
The AlkB subfamilies and their (a) nucleotide specificity. (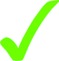 good activity; 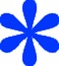 weak activity; 
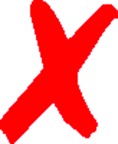
 no detectable activity). (b) Oxidative-demethylation reactions catalysed by AlkB enzymes. (c) *T*_m_ shift studies with m3T and m6A substrates. The sequence for m6A-oligonucleotides is based on the consensus sites for m6A in human.^3^

*N*-Methylation of DNA/RNA modifications can be found in the genomes of diverse organisms, including both prokaryotes and eukaryotes, and is of significant biological and clinical interest.^1,2^ There are at least 100 different modifications that are identified in cellular DNA and RNA. Some of these modifications, such as *N* ^1^-methyladenine (m1A) and *N* ^3^-methylcytosine (m3C) are damaging lesions which can lead to mutations, others are enzyme catalyzed, and have critical roles in cell biology; *N* ^5^-methylcytosine (m5C) and *N* ^6^-methyladenosine (m6A), in particular, are two of several epigenetic mechanisms for the regulation of gene expression (Fig. 1a).^3–6^ *N*-Methylation of nucleic acid can be directly reversed by the AlkB family of enzymes, of which the *Escherichia coli* AlkB is the first to be identified.^7^ Nine human homologues, ALKBH1-8 and FTO (fat mass and obesity-associated protein), have since been reported,^8^ many of these enzymes share common structural and mechanistic features with AlkB. Most of them employ ferrous iron as co-factor, and 2-oxoglutarate (2OG) as co-substrate to bring about oxidative-demethylation of *N*-methylated DNA/RNA substrates.^7^ This is achieved by oxidizing the *N*-methyl group to a hydroxymethyl group, which is coupled to the conversion of 2OG and O_2_ to succinate and CO_2_, respectively. The hydroxymethyl intermediate then fragments spontaneously to give formaldehyde and the unmodified base (Fig. 1b).

The AlkB subfamilies demonstrate notable differences in their ‘substrate specificity’ and ‘nucleotide specificity’ (Fig. 1a). With respect to ‘substrate specificity’, some homologues, such as ALKBH2 showed strong preference for double-stranded DNA/RNA, whereas others, such as FTO and ALKBH3, are specific for single-stranded DNA/RNA.^9,10^ There are also distinct differences in ‘nucleotide specificity’, where methylation on a particular nucleotide is preferentially removed; ALKBH5, for instance, selectively demethylates m6A,^11^ while AlkB is able to accept a diverse range of methylated nucleotides.^12^ The factors determining their substrate specificity remain to be elucidated, although it is beginning to emerge that this can be, at least in part, due to structural differences in the catalytic domain and the nucleotide-recognition lid domain,^12–14^ or a result of the presence of adjunct structural elements.^15,16^

Several AlkB enzymes are currently being targeted for the treatment of a range of human diseases. For instance, there is evidence that ALKBH2 and ALKBH3 both function as DNA/RNA repair enzymes *in vivo*;^17^ ALKBH3 (or prostate cancer antigen (PCA)-1), in particular, is highly expressed in prostate cancer cells and is an attractive therapeutic target for prostate cancer.^18^ Recently, ALKBH5 is reported to be involved in spermatogenesis,^11^ while the FTO gene is found to be strongly linked to obesity in genome wide association studies.^19–21^ FTO provides the strongest link to the common form of obesity, to date. However, despite the physiological connections with these enzymes, their exact molecular mechanisms remain unclear. The biological functions of the remaining human AlkB homologues are also largely unknown. With the exception of ALKBH4 and ALKBH8, which are known to catalyze the demethylation of actin K84me1, and the hydroxylation at the wobble position of tRNA, respectively, the catalytic activity of ALKBH6 and ALKBH7 remains to be clarified.^22,23^

Hence, there is strong interest in developing selective and cell-active small molecule probes of AlkB subfamilies for structural, mechanistic and functional studies, with a longer term view of validating their therapeutic potential. Although there have been few reports of inhibitors of AlkB enzymes, most, if not all, have limited or undetermined selectivity.^24–28^ To date, there is no report of compound that is selective for a specific AlkB subfamily member. In addition, most reported inhibitors bind to the 2OG-binding site; to our knowledge, the nucleotide-binding site has not been rationally explored for inhibition.

Here, we report the use of a combined approach employing crystallographic studies, molecular modelling, fluorescence-based thermal shift analyses and enzyme assays, which led to the identification of significant differences within the nucleotide-binding domains that likely regulate the nucleotide specificity of the AlkB subfamilies. We further provide proof of principle that a strategy exploiting these inherent structural differences can enable selective and potent inhibition of the AlkB subfamily, as demonstrated by the first discovery of a selective and cell-active FTO inhibitor **12**. Compound **12** not only demonstrates selectivity for FTO over other AlkB subfamilies (including ALKBH5 which, like FTO, accepts m6A substrate), it also discriminates against several other 2OG oxygenases. Such selectivity is rarely achieved for any enzyme family, and shall be of considerable interest with respect to its potential use as a functional probe; especially in dissecting the *N* ^6^-methyladenosine modulating roles of FTO and ALKBH5 to facilitate the study of *N* ^6^-methyladenosine dynamics in regulating cellular processes. The strategy outlined here is likely applicable to other AlkB subfamilies, and, more widely, to other 2OG oxygenases. Hence, it should enable the development of functional probes or therapeutic leads for these biologically and clinically important enzymes.

## Results and discussion

### Substrate selectivity of the AlkB subfamilies

To understand and to clarify the demethylase activity of the AlkB subfamilies, we first analyse the catalytic activities of all members of the AlkB family. In line with recent reports,^29–31^ our results reveal that, among the AlkB enzymes investigated, only FTO, AlkB, ALKBH1-3 and ALKBH5 are able to accept *N*-methylated DNA/RNA substrates (Fig. 1a and b). Further analysis of their nucleotide specificities reveals that FTO is the only subfamily member that is capable of removing *N*-methylation on *N* ^3^-methylthymidine (m3T) with reasonable activity; AlkB and ALKBH2,3 are also observed to demethylate m3T, however with very low efficiency, while no detectable activity is observed for all other AlkB homologues.

Consistent with this observation, analysis of the binding affinities of m3T to AlkB subfamilies by differential scanning fluorimetry (DSF, an assay which indirectly measures the effect of ligands on protein stability^32^) showed a particularly large *T*_m_ (melting temperature) shift for FTO (*T*_m_ shift = 12.1 °C), and no significant *T*_m_ shift for all other AlkB enzymes analyzed, indicating a selectivity of m3T for FTO (Fig. 1c). However, unexpectedly, the observed selectivity does not appear to extend to oligonucleotides that contain an m3T modification. In our *T*_m_ shift analyses, both the 5-mer and 15-mer m3T-containing ssDNAs, *i.e.* 5′-GG(m3T)CT-3′ and 5′-ATTGTGG(m3T)CTGCAGC-3′, gave only modest *T*_m_ shifts which are similar to those obtained for their unmethylated counterparts (Fig. 1c). Notably, both m3T-oligonucleotides are substrates for FTO. Thus, it is reasonable to speculate that the polynucleotide backbone of the DNA/RNA substrate is likely not crucial for the binding of a methylated nucleotide. Moreover, the AlkB subfamilies probably only weakly bind to their DNA/RNA substrates. This hypothesis is supported by the large *T*_m_ shift observed for ALKBH5 with m6A (*T*_m_ shift = 9.8 °C), but not with the m6A-containing ssRNAs, 5-mer 5′-GG(m6A)CU-3′ and 15-mer 5′-ATTGTGG(-m6A)CUGCAGC-3′ (Fig. 1c). Both oligonucleotide sequences are based on the consensus sites for m6A in humans and are readily demethylated by ALKBH5.

Notably, m6A did not produce a significant *T*_m_ shift for FTO or AlkB (as observed with ALKBH5), even though both enzymes also accept m6A substrates (Fig. 1c). This implies that there are likely structural elements in ALKBH5 which select strongly for m6A, in line with its exclusivity for m6A substrates.^15,16^ However ALKBH5 likely binds to m6A with a ‘non-productive’ conformation, as ALKBH5 does not demethylate m6A nucleotide. Interestingly, all of the oligonucleotides tested (which are single-stranded DNA or RNA) gave significantly lower *T*_m_ shifts for ALKBH2 compared to other AlkB enzymes; this likely reflects ALKBH2's strong preference for dsDNA/RNA over ssRNA/DNA.

### Structural differences in the nucleotide-binding site of AlkB subfamilies

**Fig. 2:**
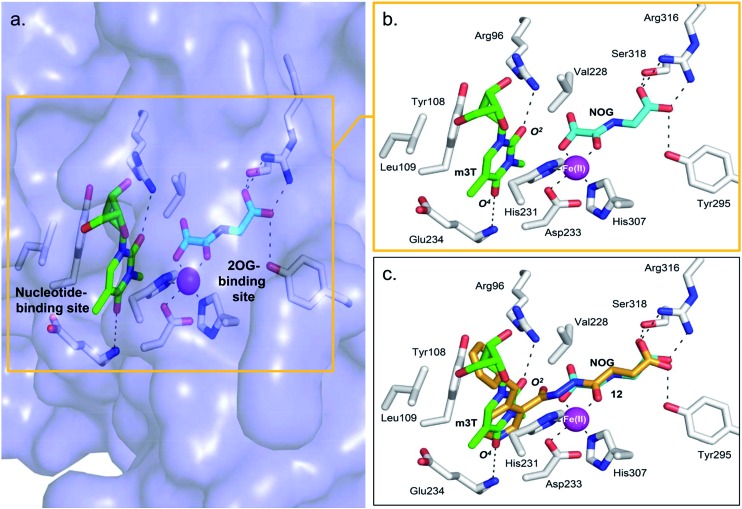
Views from a crystal structure of FTO (purple surface and white stick) bound to m3T (green) and NOG (cyan) (PDB ID 3LFM).^13^ (a) The 2OG-binding site is in close proximity to the nucleotide-binding site. (b) Close-up view showing residues that interact with m3T and NOG. (c) Superimposition of a view from a FTO-m3T-NOG structure with that of a structure of FTO in complex with **12** (orange) (PDB ID ; 4CXW). **12** is closely aligned with both NOG and m3T.

The combined results from nucleotide substrate profiling and *T*_m_ shift analyses suggest that there are likely structural features within the nucleotide-binding site of AlkB subfamilies (a deep, predominantly hydrophobic pocket in which the methylated nucleotide binds) that promote recognition and binding of a specific nucleotide (Fig. 2a). A crystal structure of FTO in complex with m3T and *N*-oxalylglycine (NOG, a catalytically inert amide analogue of 2OG) has been reported (PDB ID ; 3LFM).^13^

Structural analysis reveals that the 2OG-binding site is in close proximity to the nucleotide-binding site, and the recognition of m3T by FTO is achieved *via* interactions with six key residues within the nucleotide-binding site (Fig. 2a and b). In particular, the nucleobase ring of m3T participates in π–π interactions with the side chains of two highly conserved residues, Tyr108 and His231, while the deoxyribose ring of m3T is involved in hydrophobic interactions with the side chains of Leu109 and Val228. m3T is further stabilised by hydrogen-bonding interactions between O^2^-m3T and Arg96 (side chain, 3.1 Å), and between O^4^-m3T and Glu234 (amide backbone, 2.9 Å).

To understand the structural basis for the apparent lack of affinity of m3T for other AlkB demethylases, we superimposed the crystal structures of FTO-m3T-NOG complex (PDB ID 3LFM)^13^ with that of AlkB (PDB ID ; 3I3M),^12^ ALKBH2 (PDB ID ; 3BUC),^9^ ALKBH3 (PDB ID ; 2IUW),^31^ and ALKBH5 (PDB ID ; 4NJ4);^15^ several of these structures are in complex with their methylated oligonucleotide substrates and/or NOG. Inspection of the residues lining the nucleotide-binding sites of AlkB and ALKBH2 revealed that most of the residues that are important for m3T recognition by FTO are either conserved, or substituted by structurally equivalent residues (Fig. S1a and S1b†). The exception is Arg96_FTO_, which is substituted by Met61_AlkB_ and Gln112_ALKBH2_, leading to the loss of interactions with O^2^-m3T in AlkB and ALKBH2. Moreover, as opposed to Glu234_FTO_, the amide backbone of Asp135_AlkB_ and Asp174_ALKBH2_ are positioned too far away (average distance ∼4.6 Å) to make significant contact with O^4^-m3T. Importantly, ALKBH3 and ALKBH5 lack most of the stabilising interaction with m3T, where essential π–π interactions with the nucleobase ring and hydrogen-bonds with O^2^- and O^4^-m3T are either not conserved or significantly weakened (Fig. S1c and d†).

### Strategy for the development of subfamily-selective FTO inhibitor

Overall, our crystallographic analysis revealed significant differences in the nucleotide-binding sites of the AlkB subfamilies, which may, at least partially, rationalise their nucleotide selectivity (Fig. 1b). Importantly, these inherent structural differences may be exploited to achieve selective inhibition of AlkB subfamilies. To date, there is no report of a selective inhibitor of the AlkB enzymes, let alone a subfamily-selective inhibitor. Moreover, with the exception of FTO inhibitor, rhein,^24^ all reported inhibitors bind to the 2OG-binding site.^25–28^ The nucleotide-binding site has not been explored, at least rationally, for inhibition. We reasoned that a strategy that simultaneously occupies both the 2OG- and nucleotide-binding sites may enable both potency and selectivity (Fig. 2a).

**Scheme 1:**
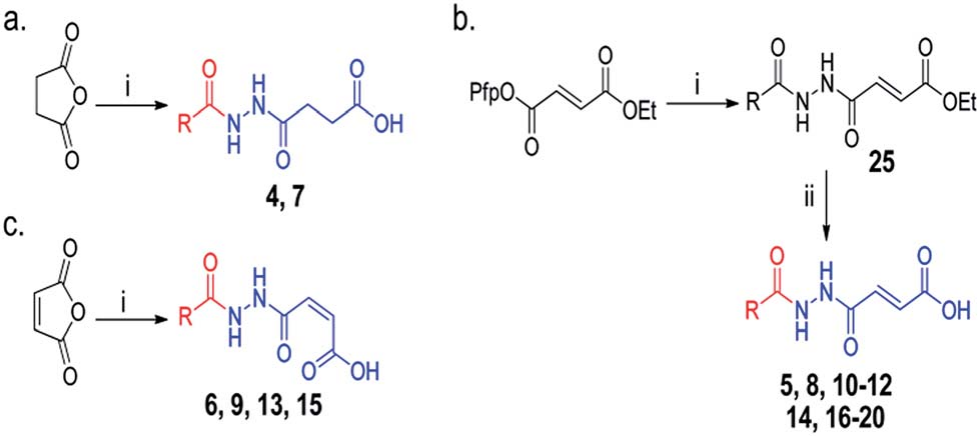
Synthesis of acylhydrazines with m3T-binding components (red) and 2OG-binding components (blue); derivatives of (a) succinate (b) fumarate and (c) maleate. Reagents and conditions: (i) monoacylhydrazines, THF–EtOAc 1 : 1, RT (24 h); (ii) LiOH, THF–H_2_O 1 : 2, RT (3 h). See Table S1† for full structures of compounds.

In the design of a ‘two-component’ inhibitor that is selective for FTO, we linked a ‘2OG-binding component’, namely succinate hydrazide **1**, fumarate hydrazide **2** or maleate hydrazide **3**, which are predicted to chelate to the active site iron in FTO with an appropriate side chain (‘m3T-binding component’) for extension into the nucleotide-binding site (Scheme 1 and Table S1†). Modelling studies based on the structures of FTO in complex with m3T and NOG identified various pyridyl side chains as having the correct relative distance and geometry for mimicking m3T interactions in the nucleotide-binding site; this gives rise to a series of acylhydrazine compounds **1–20** (Table S1†). The synthesis of the succinate and maleate derivatives involves the acylation of succinate or maleate anhydride with the desired monoacylhydrazines, while the synthesis of the fumarate derivatives uses hydrazide coupling as a key step; this involves the nucleophilic substitution of ethyl pentafluorophenyl fumarate with appropriate monoacylhydrazines, followed by basic hydrolysis to obtain the corresponding acids (Scheme 1, see ESI†).

Preliminary evaluation of compounds **1–20** for activities against FTO was achieved using both a DSF-binding assay and a HPLC-based demethylase assay which employs m3T as substrate (Fig. S2†). We identified several compounds with IC_50_ values in the low micromolar range (*i.e.* compounds **8**, **12**, **14**, and **16–20**; Table S1†); the *T*_m_ shift results supported the IC_50_ values, where compounds that were more potent gave higher *T*_m_ shifts than those that were less potent. In general, fumarate derivatives are better FTO inhibitors than the corresponding succinate and maleate derivatives. The most potent inhibitor identified is compound **12** (IC_50_ = 0.81 μM, *T*_m_ shift = 11.2 °C), which is 45-fold more active than the ‘generic’ inhibitor NOG (IC_50_ = 36.5 μM, *T*_m_ shift = 4.1 °C). To verify the HPLC-based assay results, the activity of **12** against FTO was further evaluated using a MALDI-based assay. In this assay, an m6A-ssDNA was used as substrate instead of m3T, and a comparable IC_50_ value of 1.1 μM was obtained (Fig. S3†).

**Fig. 3:**
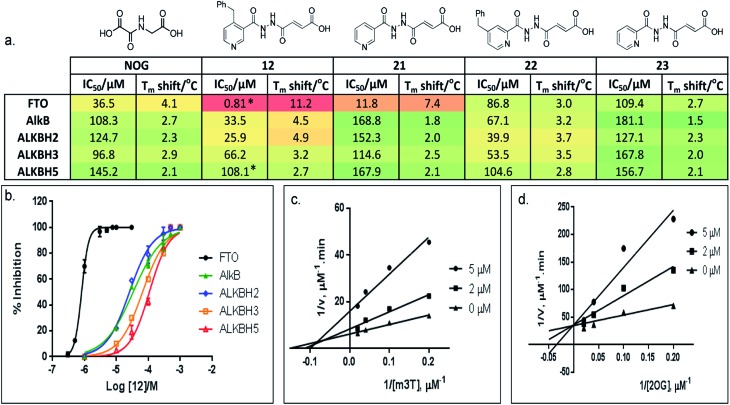
Selective inhibition of AlkB subfamilies. (a) Selectivity studies and (b) enzyme inhibition curves of **12** against representatives of AlkB subfamilies. **12** demonstrates 30-fold to 130-fold selectivity for FTO over other AlkB subfamilies. It also discriminates against other human 2OG oxygenases, as shown by a lack of inhibition for PHD2 and JMJD2A (IC_50s_ > 100 μM and >300 μM for PHD2 and JMJD2A, respectively). *FTO and ALKBH5 gave comparable IC_50s_ of 0.6 μM and 96.5 μM, respectively in HPLC-based assays that use m6A-ssRNA as substrates. (c) Kinetic studies on the mode of inhibition of FTO by **12** with respect to m3T, and (d) 2OG. **12** demonstrates a mixed mode of inhibition with respect to m3T (*K*appi = 2.1 μM, *α* = 2.0), but is a competitive inhibitor of the co-substrate 2OG (*K*appi = 1.4 μM).

Importantly, profiling studies revealed distinct selectivity of **12** for FTO, with significantly reduced inhibition and *T*_m_ shifts against AlkB (IC_50_ = 33.5 μM, *T*_m_ shift = 4.5 °C) and ALKBH2 (IC_50_ = 25.9 μM, *T*_m_ shift = 4.9 °C), and little or no inhibition or *T*_m_ shift against ALKBH3 (IC_50_ = 66.2 μM, *T*_m_ shift = 3.2 °C) and ALKBH5 (IC_50_ = 108.1 μM, *T*_m_ shift = 2.7 °C) (Fig. 3a and b). The selectivity profile of **12** is notable because it is highly consistent with the specificity of m3T for the AlkB subfamilies. This specificity is likely derived from the 4-benzylpyridyl side chain of **12** *i.e.* the ‘m3T-binding component’, as fumarate hydrazide **2** showed poor activity for FTO (IC_50_ > 100 μM, Table S1†). In addition, structurally related succinate hydrazide **1**, maleate hydrazide **3** and daminozide (a plant growth regulator that is known to inhibit KDM2/7 histone demethylases)^33^ also showed poor activities against FTO (IC_50s_ > 100 μM, Table S1†).

The lack of inhibition of **12** against ALKBH5 is also remarkable because ALKBH5, like FTO, accepts m6A in RNA as major physiological substrates.^10,11^ In the FTO and ALKBH5 assays described above, we have used m3T and/or m6A-ssDNA as substrates. To investigate if the selectivity of **12** extends to the demethylation of m6A on RNA by FTO and ALKBH5, we further evaluated the activity of **12** against both enzymes using short m6A-containing ssRNA as substrates. In both assays, comparable IC_50_ values of 0.6 μM and 96.5 μM were obtained for FTO and ALKBH5, respectively (Fig. 3a and S4†). This represents >100-fold selectivity for FTO over ALKBH5. The ability of **12** to discriminate against ALKBH5 shall make it a potential functional probe in dissecting the respective roles of FTO and ALKBH5 in m6A-mediated epigenetic processes.

### Kinetics, crystallographic analyses and mode of inhibition

A detailed kinetic study of FTO inhibition by **12** was then performed using a HPLC-based method. The analyses reveal that **12** demonstrates a mixed mode of inhibition with respect to m3T (*K*appi = 2.1 μM, *α* = 2.0), indicating that **12** binds to both FTO and FTO-m3T complex, possibly with preference for binding to free FTO (Fig. 3c). However **12** is predominantly a competitive inhibitor with respect to co-substrate 2OG (*K*appi = 1.4 μM) (Fig. 3d).

**Fig. 4:**
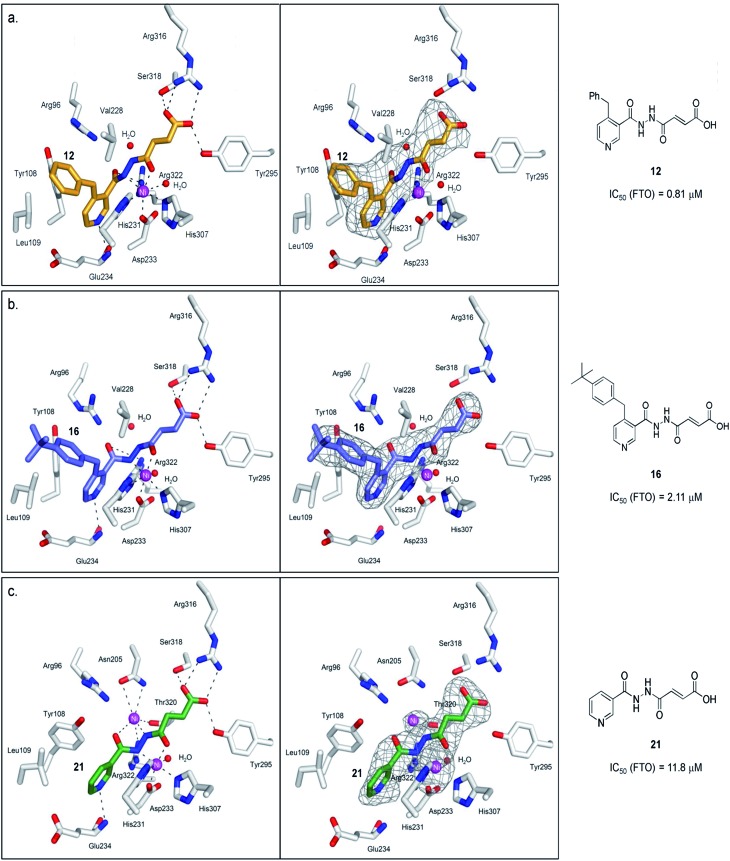
Views from structures of FTO (white sticks) bound to (a) **12** (orange stick, PDB ID ; 4CXW), (b) **16** (purple sticks, PDB ID ; 4CXX), and (c) **21** (green stick, PDB ID ; 4CXY). The unbiased difference Fourier electron density maps (*F*_o_ – *F*_c_), displayed as grey mesh, are shown to the right. Ni(ii) (magenta sphere, substituting for Fe(ii)) is coordinated by the side chains of three highly conserved residues, His231 (2.3 Å), Asp233 (2.4 Å), His307 (2.4 Å), and a putative water molecule located *trans* (2.7 Å) to His231. **12** chelates to nickel in a bidentate manner, with the C-4 carbonyl oxygen of **12** (2.1 Å) *trans* to Asp233 and the hydrazine nitrogen of **12** (2.1 Å) *trans* to His307. The C-1 carboxylate oxygens of **12** are positioned to form salt bridges to Arg316 (3.0 Å and 3.1 Å), and to hydrogen-bonds with the hydroxyl group of Tyr295 (2.7 Å), and to the side chain oxygen of Ser318 (2.9 Å). **16** binds in an analogous manner as **12**. **21** simultaneously binds to the second Ni(ii) *via* its hydrazine nitrogen (2.9 Å) and its pyridyl carbonyl oxygen (3.6 Å); this nickel is further stabilised through coordination to the side chains of Asn205 (3.2 Å, 3.3 Å), Thr320 (3.2 Å) and Arg322 (3.2 Å).

To understand the structural features that underlie the selectivity of **12**, we determined a crystal structure of FTO in complex with **12** (PDB ID ; 4CXW, Fig. 4a). As with other FTO structures, the nickel active site (substituting for iron) is coordinated by the side chains of three highly conserved residues, His231 (2.3 Å), Asp233 (2.4 Å), His307 (2.4 Å), and a putative water molecule located *trans* (2.7 Å) to His231. The ‘2OG-binding component’ of **12** *i.e.* fumarate hydrazide **2** resides in the 2OG-binding site, as predicted. It coordinates to nickel through the acylhydrazine group of **12** in a bidentate manner, with the C-4 carbonyl oxygen of **12** (2.1 Å) *trans* to Asp233 and the hydrazine nitrogen of **12** (2.1 Å) *trans* to His307. The C-1 carboxylate oxygens of **12** are positioned to form salt bridges to Arg316 (3.0 Å and 3.1 Å), and to form hydrogen-bonds with the hydroxyl group of Tyr295 (2.7 Å), and to the side chain oxygen of Ser318 (2.9 Å).

Notably, an overlay of this crystal structure with that of FTO-m3T-NOG complex (PDB ID 3LFM)^13^ reveals that the fumarate hydrazide of **12** is in close alignment with NOG (Fig. 2c). As expected, the ‘m3T-binding component’ of **12** *i.e.* the 4-benzyl pyridine side chain inserts into the nucleotide-binding site, and superimposes almost perfectly with m3T. This enables **12** to make nearly identical interactions as m3T with FTO (Fig. 2c). Hence, as with the nucleobase ring of m3T, the pyridyl ring of **12** is sandwiched between the side chains of Tyr108 and His231 to enable similar π–π interactions. In addition, the pyridyl nitrogen is situated in the same relative position as O^4^-m3T, and forms analogous hydrogen-bonding interactions with the amide backbone of Glu234 (2.9 Å). Although no corresponding interaction with Arg96 is observed for **12**, this is presumably compensated for by an additional hydrogen-bond between the pyridyl carbonyl oxygen of **12** and the side chain of Arg322 (2.8 Å, Fig. 4a). The 4-benzyl substituent of **12** occupies equivalent position as the sugar ring of m3T, and participates in similar hydrophobic interactions with Leu109 and Val228. There is no significant difference in the overall conformations of all the residues in both crystal structures (RMSD of all atoms = 0.5 Å). Similar mode of binding is also observed for **16** (an analogue of **12**, Table S1†) as determined by a crystal structure of FTO in complex with **16** (PDB ID ; 4CXX, Fig. 4b).

**Fig. 5:**
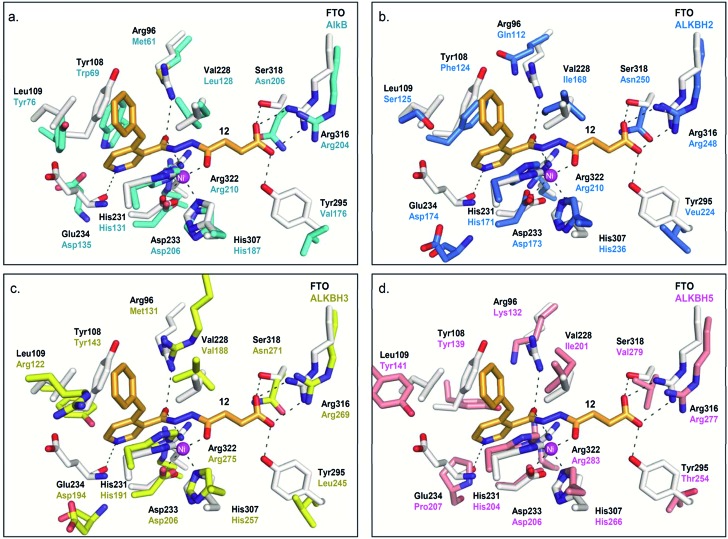
Rationalisation for the selectivity of **12** for FTO over other AlkB subfamilies. Superimposition of views from the crystal structure of FTO-**12** complex (white residues and orange stick, PDB ID ; 3LFM) with a structure of (a) AlkB (cyan residues) (PDB ID ; 3I3M),^12^ (b) ALKBH2 (blue residues) (PDB ID ; 3BUC),^9^ (c) ALKBH3 (yellow residues) (PDB ID ; 2IUW),^31^ and (d) ALKBH5 (salmon residues) (PDB ID ; 4NJ4).^15^ The interaction between the pyridyl nitrogen of **12** and Glu234_FTO_ is likely crucial for its selectivity; **12** is unable to make equivalent interactions with Asp135_AlkB_, Asp174_ALKBH2_, Asp194_ALKBH3_ and Pro207_ALKBH5_.

Modelling study reveals that most of the interactions between **12** and the 2OG-binding site of FTO are conserved in AlkB and ALKBH2,3,5 (Fig. 5). There are, however, significant differences in the interactions of **12** with the nucleotide-binding sites of these demethylases. In particular, the corresponding interaction between the pyridyl nitrogen of **12** and Glu234_FTO_ is either abolished or significantly weakened in all other subfamilies investigated; apparently, the equivalent residues *i.e.* Asp135_AlkB_, Asp174_ALKBH2_, Asp194_ALKBH3_ and Pro207_ALKBH5_ are too distant to make significant contact with the pyridyl nitrogen. In addition, unlike Tyr108_FTO_, Trp69_AlkB_ and Phe124_ALKBH2_, which are involved in π–π interactions with the pyridyl ring of **12**, the corresponding residues in ALKBH3 (Tyr143) and ALKBH5 (Tyr139) are unable to achieve the correct alignment for similar stacking interactions. These factors may, at least in part, rationalise the selectivity of **12** for FTO over other AlkB subfamilies.

The lack of activities of **12** for AlkB is interesting because it has been suggested that the substrate binding ‘lid’ of AlkB is conformationally flexible, and this likely enables AlkB to accept a diverse range of alkylated nucleotide substrates.^9,34^ Hence, it is conceivable that AlkB undergoes a similar conformational change at its nucleotide-binding site to accommodate the binding of **12**. However, superimposition of a crystal structure of the AlkB-dsDNA complex (PDB ID: ; 3I3Q)^14^ with the unliganded AlkB proteins (PDB ID: ; 4NID)^35^ reveals no significant change in the conformation of residues lining the nucleotide-binding site of AlkB (RMSD of all atoms = 0.2 Å, Fig. S5†). Consistent with studies by others,^36^ our data indicate that the flexibility of AlkB is largely localised to the Asp135-Pro142 loop, where movement is observed with Asp135 (Fig. S5a†). Modelling studies with FTO and ALKBH5 also reveal minimal conformational change in the nucleotide-binding sites of both enzymes on binding with their respective substrates (RMSD of all atoms = 0.6 Å (FTO), 0.4 Å (ALKBH2), and 0.3 Å (ALKBH5), Fig. S5†); we were unable to obtain an accurate model of ALKBH3. In line with previous studies, the only notable conformational change observed is the ‘flipping’ of the side chain of Tyr141_ALKBH5_ in the presence of nucleotide substrate.^15^ Overall, our available results imply that the nucleotide-binding sites of AlkB, FTO, and ALKBH2,5 likely remain unchanged upon interaction with **12**.

To probe the roles of the respective residues within the nucleotide-binding site for selective inhibition, we further synthesised compounds **21–23** using the methods described in Scheme 1 (Fig. 3a; see the ESI†). Activity studies suggest that the residues involved in sugar ring interactions are important for potency for all AlkB subfamilies, as **21**, which lacks the participating 4-benzyl group of **12**, has greatly reduced activity against all subfamilies tested (IC_50_ (FTO) = 11.8 μM, IC_50s_ (ALKBH2,3,5) > 100 μM). These interactions, however, are likely not crucial for selectivity, as **21** retains >10-fold selectivity for FTO. In contrast, the interactions with Glu234 appear to be critical, as demonstrated by the dramatic loss of potency and selectivity of the 2-pyridyl compounds, **22** (IC_50_ = 86.8 μM) and **23** (IC_50_ = 109.4 μM), compared with their 3-pyridyl parents, **14** (IC_50_ = 1.02 μM) and **21** (IC_50_ = 11.8 μM), respectively (we were unable to synthesise the 2-pyridyl analogue for **12**). By inference from a crystal structure of FTO in complex with **21** (PDB ID ; 4CXY, Fig. 4c), this subtle switch in the pyridyl nitrogen position prevents **22** and **23** from forming hydrogen-bonds with Glu234. Hence, the results indicate that Glu234 is likely a key determinant for substrate specificity for FTO; this is consistent with a previous report where site-directed mutagenesis of Glu234 abolished FTO demethylase activity on m3T.^13^

In general, arylhydrazine compounds are expected to chelate to metal in solution.^37^ Thus, it is possible that **12** inhibits FTO predominantly through a depletion of iron in solution, or from sequestration of iron at the active site of FTO. However, we observed that the IC_50s_ for **12** and most of its analogues (*i.e.* **14**, **16–20**) are 25-fold to 100-fold less than the concentration of iron (100 μM) used in the biochemical assays. There was also no significant change in the IC_50_ of **12** when the iron concentration was increased from 100 μM to 500 μM (results not shown). These results indicate that the inhibitory activity of **12** is not dependent on the concentration of iron. It also provides evidence that the mode of inhibition of **12** is, at least in part, a result of specific interactions in the catalytic domain of FTO, and not predominantly due to non-specific chelation to iron.

Interestingly, crystallographic analysis of the FTO in complex with **21** (PDB ID ; 4CXY) showed distinct electron density within the 2OG-binding site, which is consistent with the presence of a second nickel that is not normally present (Fig. 4c). **21** apparently induces binding to a second nickel *via* its hydrazine nitrogen (2.9 Å) and its pyridyl carbonyl oxygen (3.6 Å); this nickel is further stabilised by chelation to the side chains of Asn205 (3.2 Å, 3.3 Å), Thr320 (3.2 Å) and Arg322 (3.2 Å). However, the observed two-metal binding is likely dependent on enzyme, as previous mass spectrometric and docking studies of **21** with PHD2 (prolyl hydroxylase domain-containing protein 2) did not indicate two-metal binding.^37^ Moreover, not all acylhydrazines can simultaneously bind to a second metal; in the crystal structures of the FTO-**12** and FTO-**16** complexes, the corresponding position for the second nickel is occupied by a water molecule under our crystallographic conditions (Fig. 4a and b).

### Selectivity profiling and cellular activity

To investigate the selectivity of **12** against other Fe(ii)- and 2OG-dependent oxygenases, **12** was tested for inhibition against two clinically important human 2OG oxygenases, PHD2 ^38,39^ (an oxygen sensing enzyme, which is central to hypoxic response) and JMJD2A^40,41^ (jmjC histone lysine demethylase, targeted for the treatment of cancer). **12** showed IC_50s_ > 100 μM and >300 μM for PHD2 and JMJD2A, respectively, thus demonstrating 100–300 fold selectivity towards FTO. Notably, **21** is a known potent inhibitor of PHD2 (IC_50_ (PHD2) = 82 nM).^31^ The lack of inhibition of **12** for PHD2 and for JMJD2A further demonstrates that the proposed strategy based on nucleotide specificity can enable compounds with the desired selectivity profile. Recently, nucleic acid demethylases from other family have been identified, such as the ten-eleven translocation (TET) enzymes, which are known to regulate the level of 5-methylcytosine (m5C) in CpG regions.^42^ Further characterisation of **12** against DNA/RNA demethylases from a ‘non-AlkB’ family will be important in defining its utility as a chemical probe.

**Fig. 6:**
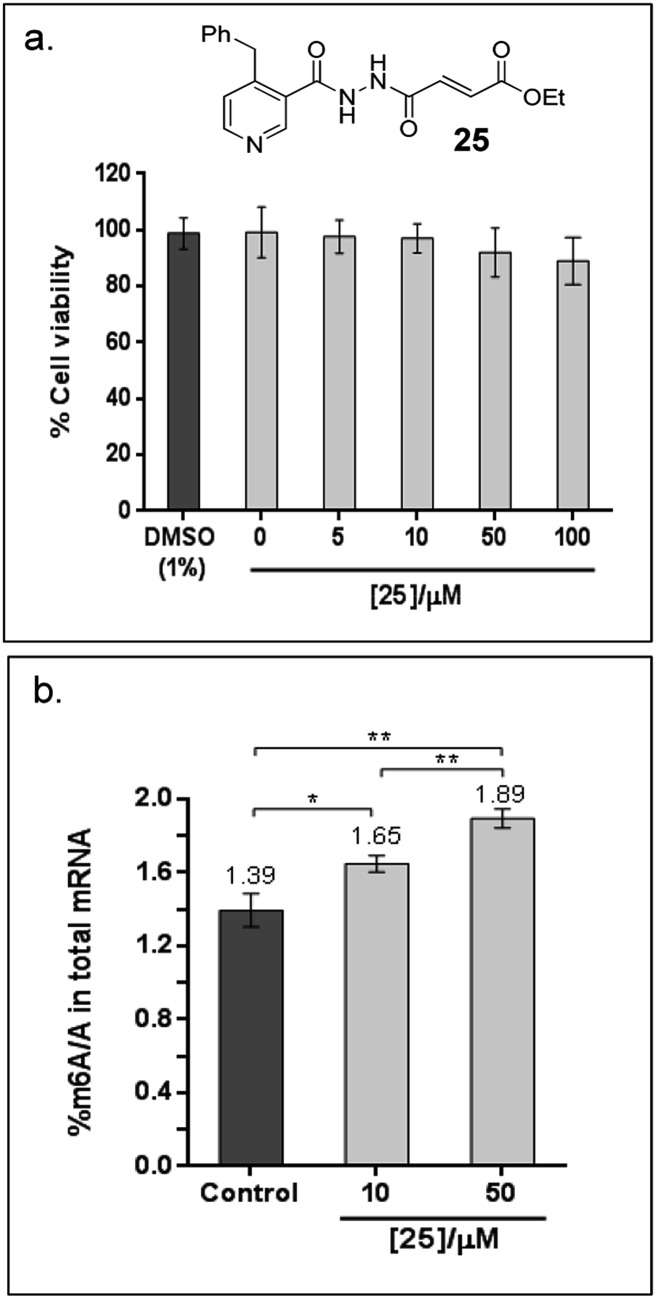
Cellular efficacy of **25**, an ethyl ester derivatives of **12**. (a) Effects of **25** on the viability of HeLa cells, as determined by MTT cytotoxicity assay. The data shows the mean of 3 technical replicates ± s.e.m of 2 separate experiments. (b) There is significant increase in % m6A/A in HeLa cells treated with 10 μM and 50 μM **25** for 6 h compared to the untreated control (1% DMSO), as revealed by Student's *t*-test (*P < 0.05; **P < 0.01). The data shows the mean of 3 technical replicates ± s.e.m of 2 separate experiments.

Finally, to explore the cellular efficacy of **12**, we tested the ethyl ester derivative of **12** *i.e.* **25** on HeLa cells. Preliminary study on the intracellular hydrolysis of **25** reveals that about 35% of **25** that entered the cells is hydrolysed to **12** (for the method, see the ESI†). Notably, despite potential stability and toxicity concerns associated with acylhydrazines in general,^33^ >80% of the cells remain viable after treatment with 100 μM of **25**, as determined by an MTT assay (Fig. 6a). Cells that were treated with **25** showed a significant increase in the level of m6A modification in the total mRNA compared to the untreated control; in particular, the percentage of m6A in mRNA increased by 19% and 36% after treatment with 10 μM or 50 μM of **25**, respectively (Fig. 6b). Hence, the observed effect appears to be concentration dependent, and is, at least partially, due to the inhibition of FTO m6A demethylase activity by **25** in cells. Studies on the selectivity of **25** *in vivo*, and its cellular mechanisms are currently underway. These results will provide insight on the usefulness of **25** as a functional probe, and will be reported in due course.

## Conclusions

Overall we report the combined crystallographic, molecular modelling, *T*_m_ shift, and biochemical studies on the determinants that regulate nucleotide specificity within the AlkB subfamilies. Although structural elements, such as the nucleotide-recognition lid domain and the L1 loop are likely involved in the recognition and/or selection of methylated substrates, some of these domains are not present in all AlkB subfamilies; to date, only FTO and AlkBH5 were reported to possess the L1 loop. Consistent with recent study by others, we demonstrated that the substrate specificity of the AlkB enzymes can arise, at least in part, from structural differences within their nucleotide-binding sites. Notably, Glu234_FTO_ is likely a key residue that determines the affinity and specificity of FTO for its substrates.

We further provide proof of principle that a strategy exploiting these inherent structural differences in the nucleotide-binding sites of AlkB subfamilies can enable selective and potent inhibition of the AlkB enzymes. Through this approach, we identified compound **12** as a potent and subfamily-selective inhibitor of the medically important FTO. Crystallographic analysis reveals that **12** occupies a previously unexplored region of the substrate binding site. Selectivity profiling demonstrates that **12** not only exhibits 30-fold to 130-fold selectivity for FTO over other AlkB subfamilies, it also discriminates against other human 2OG oxygenases, as shown by a lack of inhibition for PHD2 and JMJD2A. The cell-based assay further indicates that **25**, the ethyl ester derivative of **12**, is able to inhibit m6A demethylase activity in cells. To our knowledge, this is the first time that an inhibitor which is selective for a particular AlkB subfamily member is reported. Such selectivity profile shall be of widespread interest with regard to its potential use as a functional probe and, possibly, therapeutic lead.

Importantly, **12** showed clear selectivity for FTO over ALKBH5. To date, FTO and AlkBH5 are the only two enzymes known to demethylate m6A substrate in human. Notably, m6A is one of the most abundant post-translational modifications in eukaryotes RNA, and is widely considered to be a novel epigenetic mark. There is increasing evidence that the dynamic m6A modification is central to the regulation of a wide range of cellular processes. **12** should, therefore, greatly facilitate the study of the respective roles of FTO and ALKBH5 in m6A-mediated epigenetic processes.

It is envisaged that the strategy outlined here is generally applicable to the selective inhibition of other AlkB subfamilies and, more widely, to other 2OG oxygenases. A challenge will be to apply this strategy to the discovery of selective probes or therapeutic leads for every member of this biologically and clinically important class of enzymes.

## Conflict of interest

The authors declare no competing financial interest.
