# Supplementary material for: A strategy based on nucleotide specificity leads to a subfamily-selective and cell-active inhibitor of N 6-methyladenosine demethylase FTO
Source: Chem Sci. 2014 Sep 22;6(1):112–22. doi: 10.1039/c4sc02554g (PMC5424463; doi:10.1039/c4sc02554g)
Supplement: Supplementary file 1 [file SC-006-C4SC02554G-s001.pdf]

Electronic Supplementary Material (ESI) for Chemical Science.
This journal is © The Royal Society of Chemistry 2014

Electronic Supporting Information

A Strategy Based on Nucleotide Specificity Leads to Subfamily-Selective and Cell-Active Inhibitor of N^6-Methyladenosine Demethylase FTO†

Joel D. W. Toh,‡[ab] Lingyi Sun,‡[a] Lisa Z. M. Lau,[a] Jackie Tan,[c] Joanne J. A. Low,[a] Colin W.Q. Tang,[a] Eleanor J. Y. Cheong,[a] Melissa J. H. Tan,[a] Yun Chen,[c] Wanjin Hong,[b] Yong-Gui Gao*[bc] and Esther C. Y. Woon*[a]

[a]*Department of Pharmacy, National University of Singapore, 18 Science Drive 4, 117543, Singapore.*
[b]*Institute of Molecular and Cell Biology, 61 Biopolis Drive, Proteos, 138673, Singapore*
[c]*School of Biological Sciences, Nanyang Technological University, 60 Nanyang Drive, 637551, Singapore.*

* To whom correspondence should be addressed.

Esther C. Y. Woon
Email: esther.woon@nus.edu.sg Fax: Tel: (+65) 6516 2932; (+65) 6779 1554. Address: Department of Pharmacy, National University of Singapore, 18 Science Drive 4, 117543, Singapore.

Yong-Gui Gao
Email: ygao@ntu.edu.sg, Fax: (+65) 6779 1117. Address: School of Biological Sciences, Nanyang Technological University, 60 Nanyang Drive, 637551, Singapore.

Contents:

Figure S1. Structural differences in the nucleotide-binding sites of AlkB subfamilies. S3

Figure S2. HPLC-based activity assay of **12** against FTO. S4

Figure S3. MALDI-based activity assay of **12** against FTO. S5

Figure S4. Enzyme inhibition curves of **12** against FTO and ALKBH5 as determined by HPLC-based assay using m6A-ssRNA as substrate. S6

Figure S5. Conformational changes in the nucleotide binding sites of AlkB, FTO and ALKBH5 on association with nucleotide substrates. S7

Table S1. Inhibitory activities and T_m shift analysis of acylhydrazine compounds **1-20** against FTO. S10

Table S2. Crystallographic data collection and refinement statistics S11

Materials and methods

Protein expression and purification S12

Protein crystallography S15

Differential scanning fluorimetry S15

Inhibition assays S16

Kinetic of FTO inhibition by **12** S21

MTT cell viability assay S21

Determination of intracellular hydrolysis of **25** S21

Cell-based inhibition assay S22

Chemical synthesis S23

Scheme for the synthesis of **12-19** S24

Scheme for the synthesis of **13** S24

Scheme for the synthesis of **14** S25

Scheme for the synthesis of **15** S25

Scheme for the synthesis of **20** S26

Scheme for the synthesis of **22** S26

NMR spectra for selected compounds S59

References S79

Author contributions S81

Figure S1. Structural differences in the nucleotide-binding sites of representative AlkB subfamilies. Superimposition of views from the crystal structure of m3T (green stick) in complex with FTO (white residues) (PDB ID 3LFM)[1] with a structure of (a) AlkB (cyan residue) (PDB ID 3I3M)[2], (b) ALKBH2 (blue residue) (PDB ID 3BUC)[3] , (c) ALKBH3 (yellow residue) (PDB ID 2IUW)[4], and (d) ALKBH5 (salmon residue) (PDB ID 4NJ4)[5]. m3T is stabilized by hydrogen-bonding interactions between O^2-m3T and the side chain of $Arg96_{FTO}$ (3.1 Å), and between O^4-m3T and the amide backbone of $Glu234_{FTO}$ (2.9 Å). $Arg96_{FTO}$ is substituted by $Met61_{AlkB}$ and $Gln112_{ALKBH2}$, leading to the loss of interactions with O^2-m3T in AlkB and ALKBH2; corresponding interactions with $Arg131_{ALKBH3}$ and $Lys132_{ALKBH5}$ are significantly weakened. The amide backbone in $Asp135_{AlkB}$, $Asp174_{ALKBH2}$, $Asp194_{ALKBH3}$ and $Pro207_{ALKBH5}$ are positioned too far away to make significant contact with O^4-m3T. In ALKBH3 and ALKBH5, π-π interactions with the side chains of $Tyr108_{FTO}$ are not conserved.

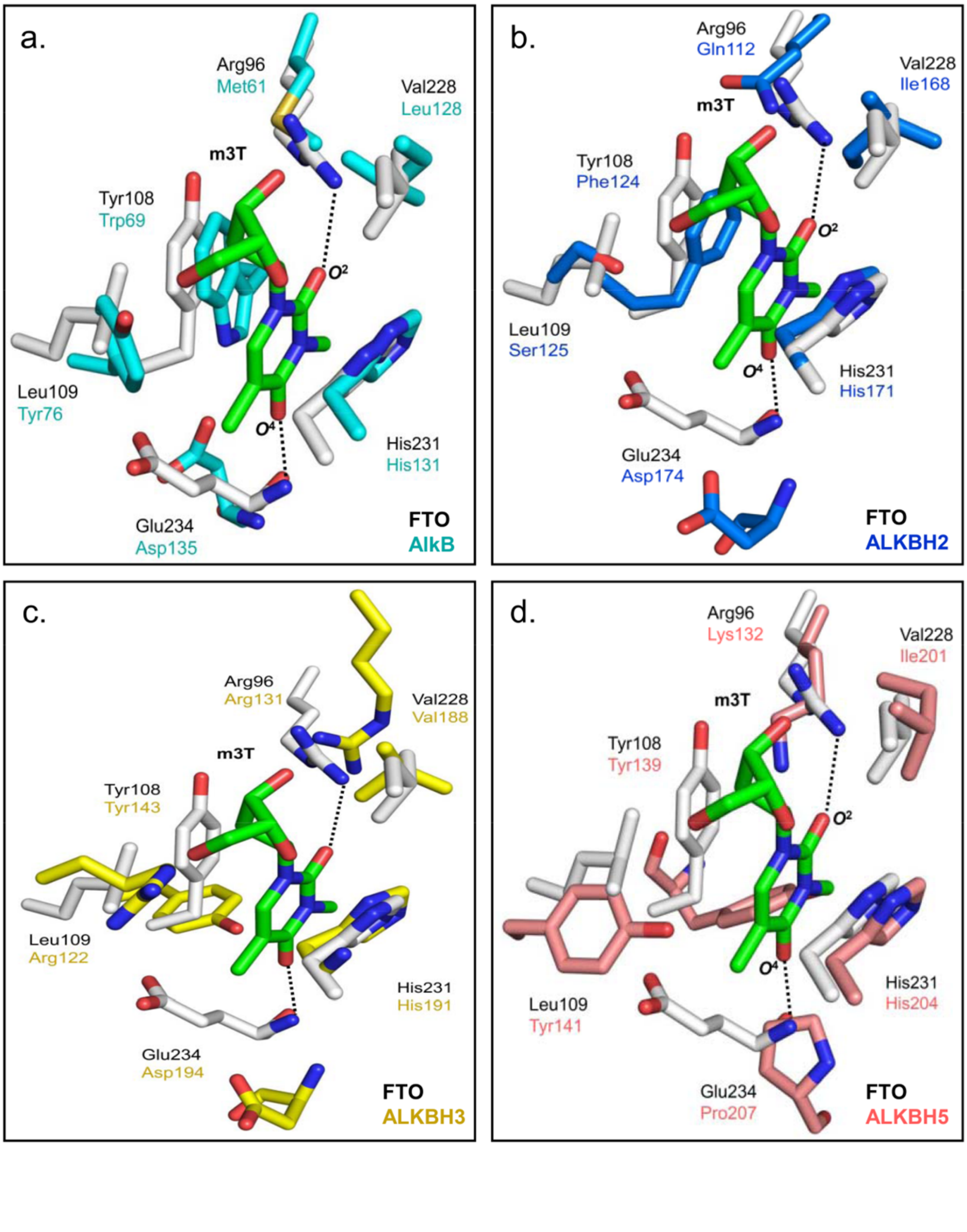

Figure S2. HPLC-based activity assay of **12** against FTO. HPLC traces showing demethylation of m3T substrate (Rt = 7.3 min) by 1 μM FTO to give thymidine product (Rt = 6.4 min) in the presence of (a) 0 μM **12**, and (b) 1 μM **12**. About 20% of the m3T substrate is converted to thymidine product after 1 hour incubation at 4°C in the absence of inhibitor.

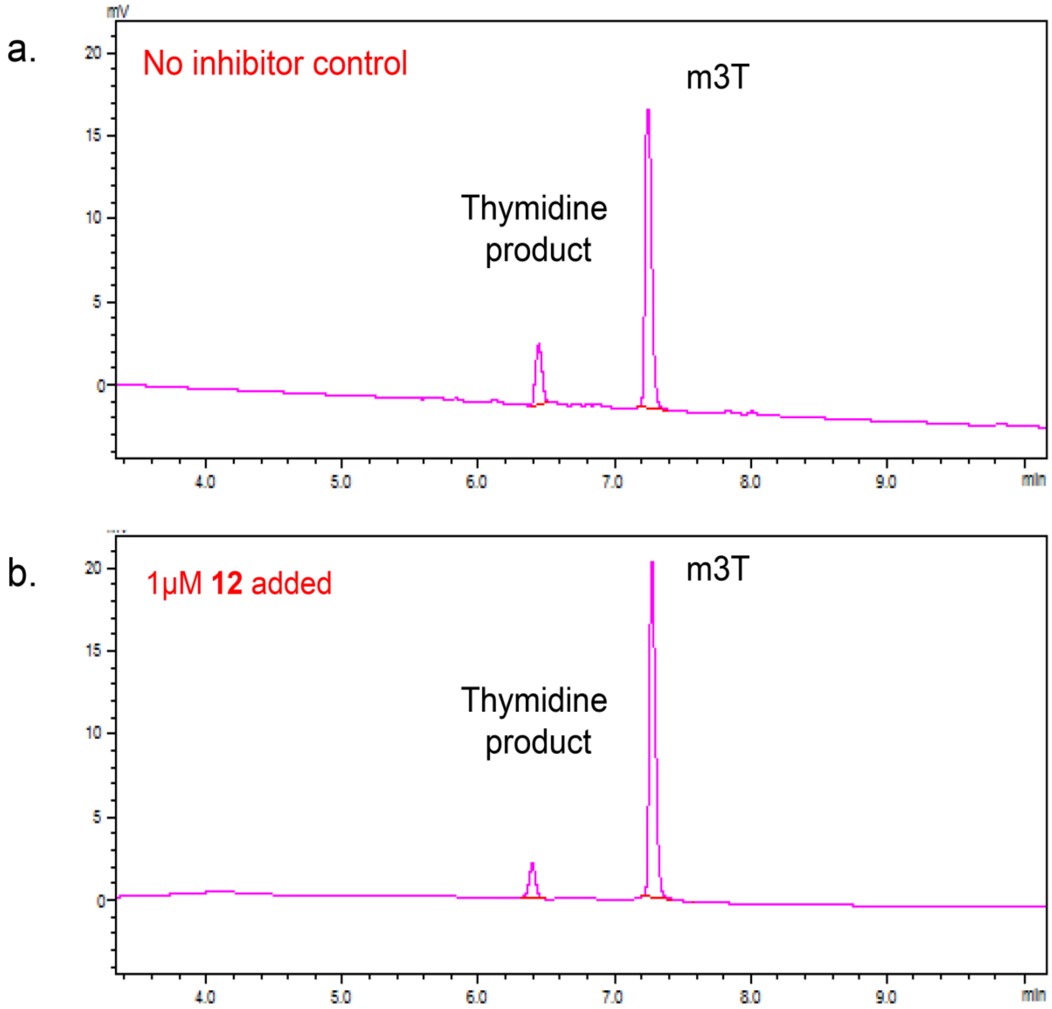

Figure S3. MALDI-based activity assay of **12** against FTO. MALDI-TOF MS spectra showing demethylation (-14 Da) of an 8-mer m6A-ssDNA substrate (TCA(m6A)CAGC) (2392.62 Da) by 2 μM FTO (a) in the absence of **12**, and (b) in the presence of 1 μM **12**. The percentage inhibition was estimated based on the relative intensities of the methylated substrate and the demethylated products.

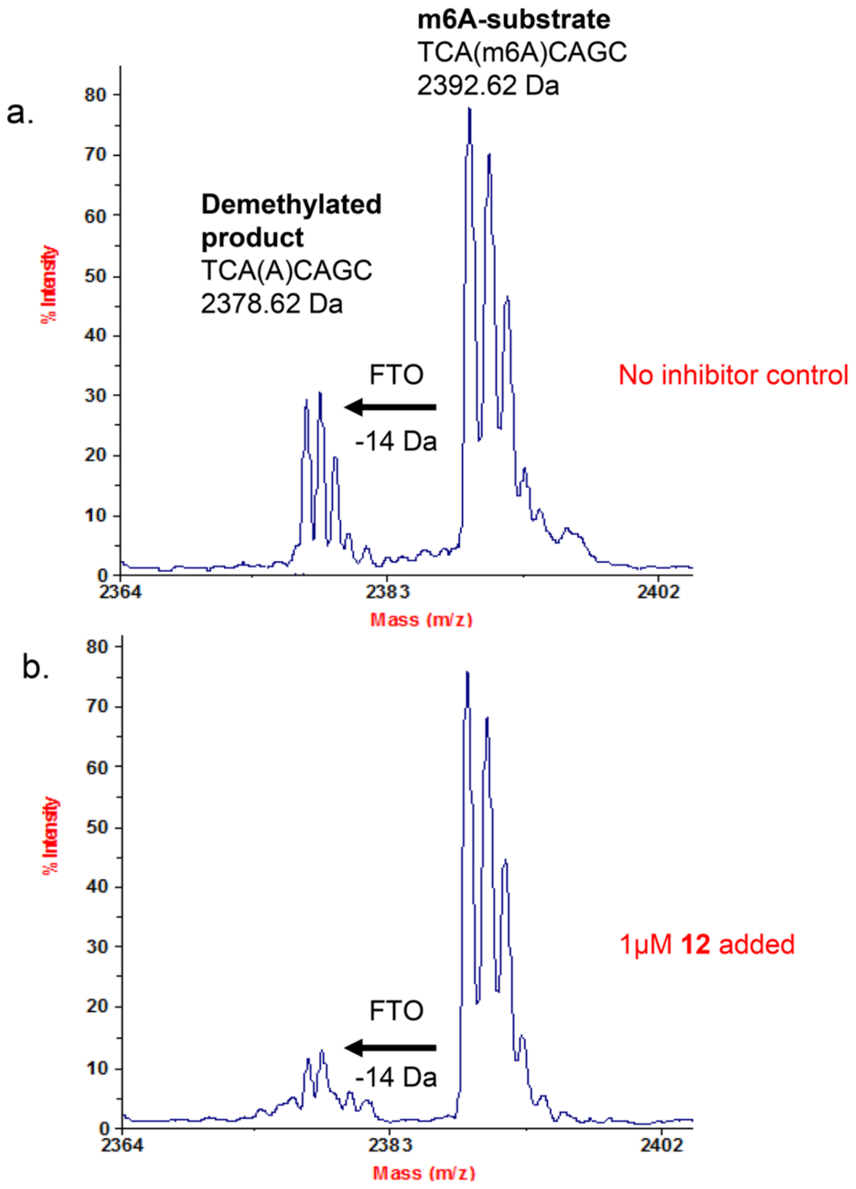

Figure S4. Enzyme inhibition curves of **12** against FTO and ALKBH5, as determined by a HPLC-based assay using m6A-containing ssRNA substrates. The substrate sequences for the FTO and ALKBH5 assays are 5'-CUUGUCA(m6A)CAGCAGA-3' and 5'-GG(m6A)CU-3', respectively. **12** gave IC_{50} values of 0.6 μM and 96.5 μM for FTO and ALKBH5, respectively, which represents >100-fold selectivity for FTO over ALKBH5.

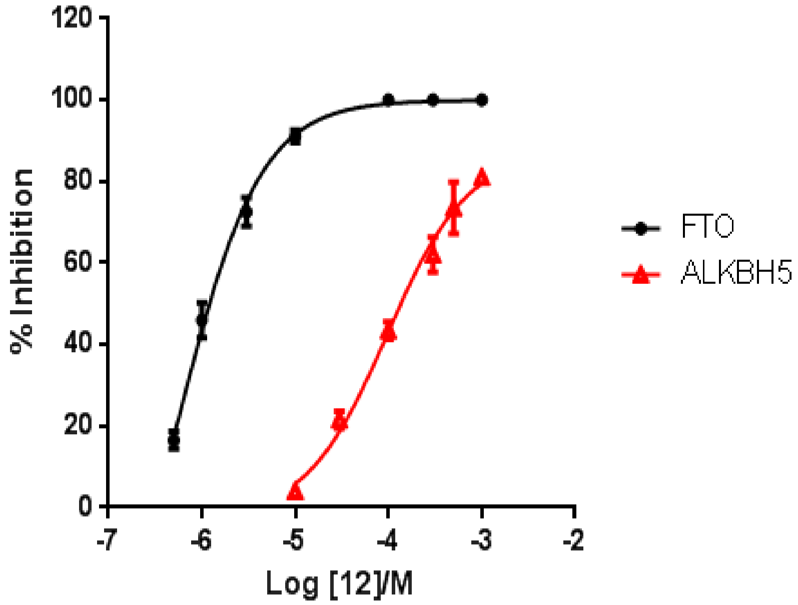

Figure S5. Conformational change in the nucleotide-binding sites of AlkB, FTO, ALKBH2 and ALKBH5 on association with their respective nucleotide substrates. Superimposition of views from the crystal structure of (a) the unliganded AlkB (white ribbon and residues, PDB ID: 3I3Q)[2] with AlkB-dsDNA complex (blue ribbon and residues, PDB ID: 4NID)[6]; (b) unliganded FTO (white ribbon and residues, PDB ID 3LFM)[1] with an model of FTO-GG(m6A)CU complex (orange ribbon and residues); (c) a model of unliganded ALKBH2 (white ribbon and residues) with ALKBH2-dsDNA complex (purple ribbon and residues, PDB ID 3BUC);[3] (d) unliganded ALKBH5 (white ribbon and residues, PDB ID 4NRO)[7] with a model of ALKBH5-GG(m6A)CU complex (salmon ribbon and residues). The liganded FTO and liganded AKLBH5 models were obtained by docking 5'-GGm6ACU-3'substrate onto FTO and ALKBH5 crystal structures, respectively, followed by solvation and energy minimisation using the YASARA energy minimisation server (http://www.yasara.org/minimizationserver.htm). Only minimal conformational change was observed for residues lining the nucleotide-binding site of AlkB, FTO and ALKBH5 on association with their respective oligonucleotide substrates; RMSD of all atoms = 0.2 Å (AlkB), 0.6 Å (FTO), 0.4 Å (ALKBH2), and 0.3 Å (ALKBH5). For clarity, the nucleotide substrates are not shown.

(a)

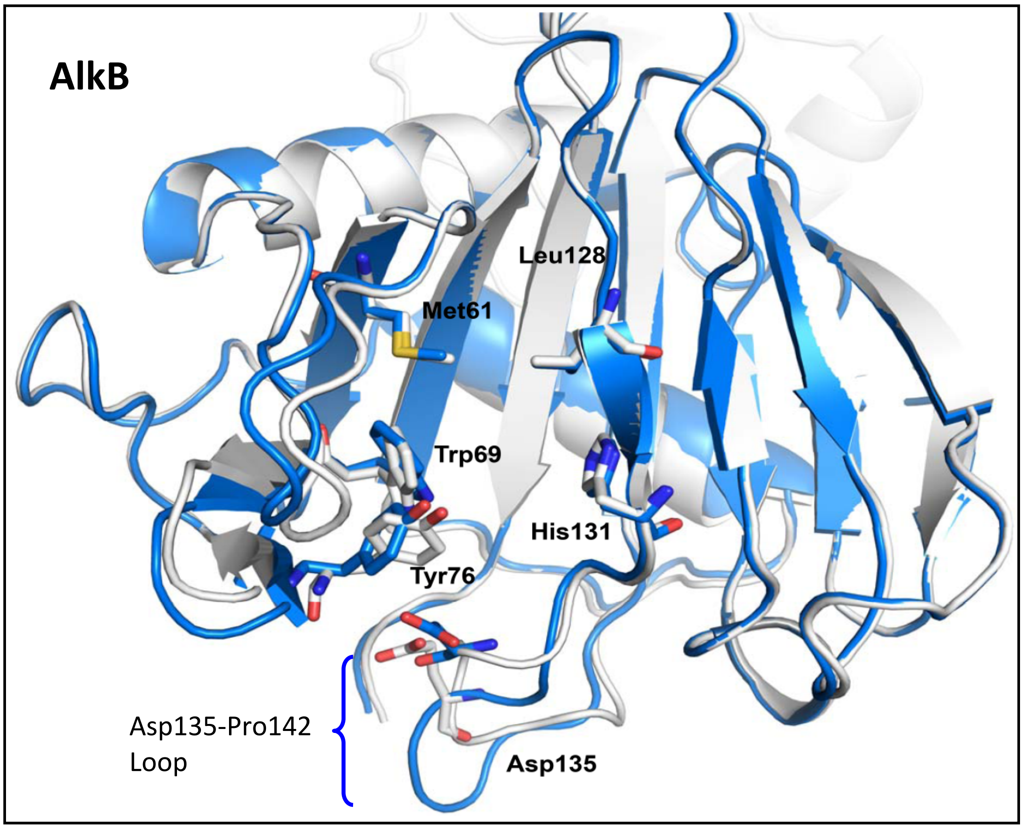

(b)

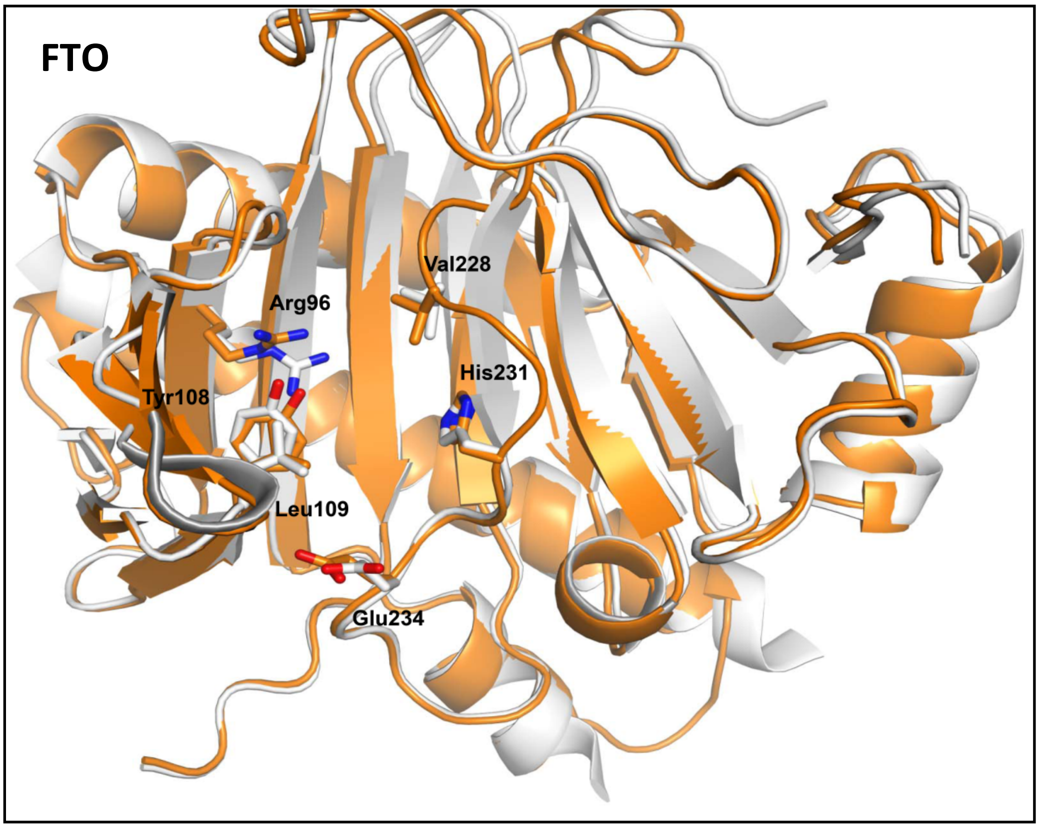
FTO
Val228
Arg96
His231
Tyr108
Leu109
Glu234

(c)

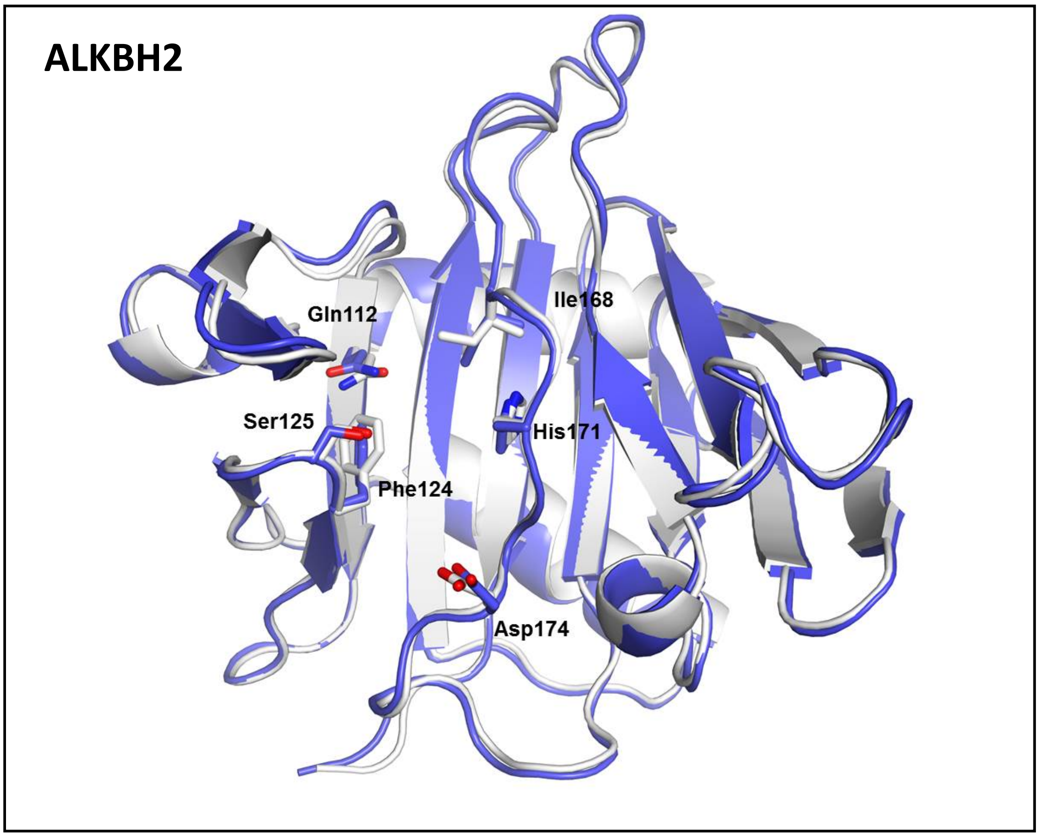
ALKBH2
Gln112
Ile168
Ser125
His171
Phe124
Asp174

(d)

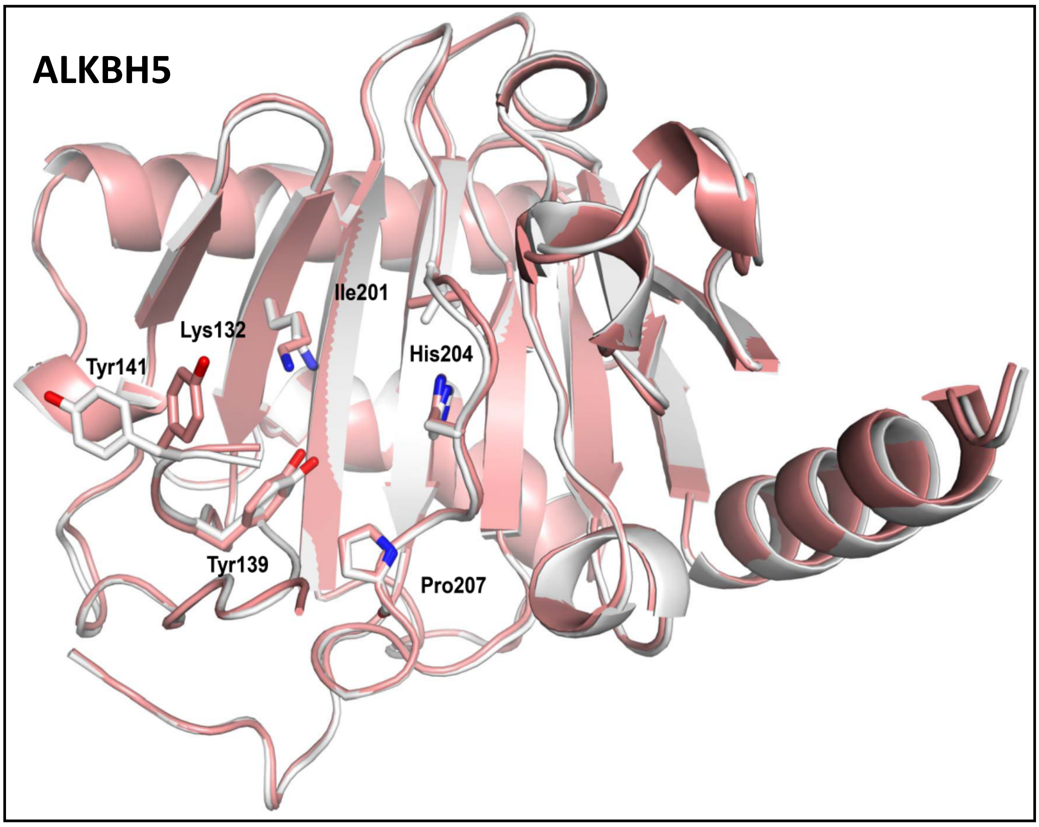

Table S1. Inhibitory activities and T_m shift analyses of acylhydrazine compounds **1-20** against FTO. IC_{50s} were determined with a HPLC-based assay, using eight different concentrations of inhibitors (0, 1, 3, 0, 30, 100, 300, 1000 μM). T_m shift was determined using a Differential Scanning Fluorimetry (DSF)-based binding assay. Details of the assays are given in the methods section. All assays were performed in triplicate for each inhibitor. The % inhibitions at a final compound concentration of 100 μM are in parentheses.

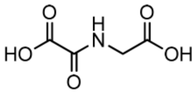
NOG
IC_{50} = 36.5μM
T_m shift = 4.1°C

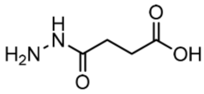
Succinate hydrazide 1
IC_{50} >100μM

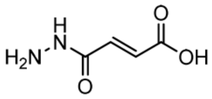
Fumarate hydrazide 2
IC_{50} > 100μM

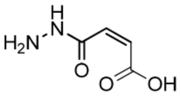
Maleate hydrazide 3
IC_{50} > 100μM

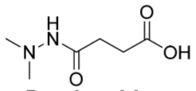
Daminozide
IC_{50} >100μM (35%)

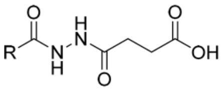
A

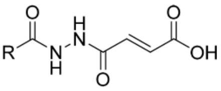
B

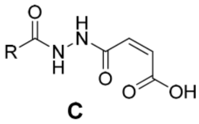
C

Compound	Scaffold	2OG-Binding Component	m3T-Binding Component (R)	IC_{50}/μM (FTO)	T_m shift/°C (FTO)
4	A	Succinate hydrazide	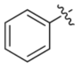	(32%)	0.7
5	B	Fumarate hydrazide		(54%)	2.5
6	C	Maleate hydrazide		(12%)	0.1
7	A	Succinate hydrazide	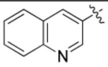	87.4	3.0
8	B	Fumarate hydrazide		3.27	7.2
9	C	Maleate hydrazide		(29%)	0.5
10	B	Fumarate hydrazide	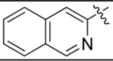	75.7	3.1
11	B	Fumarate hydrazide	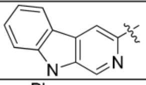	(48%)	1.6
12	B	Fumarate hydrazide	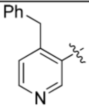	0.81	11.2
13	C	Maleate hydrazide		69.4	3.2
14	B	Fumarate hydrazide	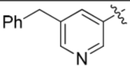	1.02	9.5
15	C	Maleate hydrazide		59.6	3.5

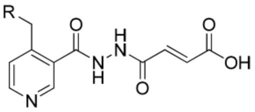

Compound	R	IC_{50}/μM (FTO)	T_m shift/°C (FTO)
16	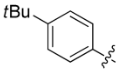	2.11	7.9
17	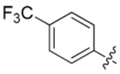	3.05	7.4
18	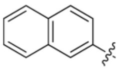	1.70	9.0
19	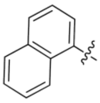	1.48	9.1
20	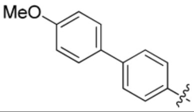	1.28	9.5

Table S2. Crystallographic data collection and refinement statistics.

	**12**	**16**	**21**
PDB ID	4CXW	4CXX	4CXY
Resolution Range (Å)	50.000-3.050 (3.10-3.05)	49.181-2.710 (2.76-2.71)	50.000-2.600 (2.65-2.60)
Space Group	H3	H3	H3
Unit Cell Dimensions (*a*Å, *b*Å, *c*Å, α°, β°, γ°)	141.6, 141.6, 83.8 90, 90, 120	140.7, 140.7, 83.3 90, 90, 120	141.9, 141.9, 83.8 90, 90, 120
Total number of Reflections Observed	59141	67330	101120
Number of unique reflections#	22939 (3570)	16658 (2707)	38122 (5970)
Redundancy#	2.6 (2.6)	4.0 (3.9)	2.6 (2.6)
Completeness (%)#	95.2 (90.5)	99.5 (99.4)	98.6 (95.6)
I/σ(I) #	11.3 (2.0)	18.0 (1.9)	13.5 (1.0)
§R_{merge} (%)#	6.1 (50.5)	4.5 (70.3)	5.1 (98.4)
*R_{work} (%)	18.3	21.0	21.2
†R_{free} (%)	24.6	26.3	24.5
¶RMS Deviation	0.011 (1.385°)	0.011 (1.315°)	0.010 (1.398°)
‡Average B Factor (Å^2)	94.2 (91.6)	90.1 (76.1)	84.5 (77.0)
Number of water molecules	65	113	103

§ $R_{merge} = \sum_j \sum_h | I_{hj} - \langle I_h \rangle | / \sum_j \sum_h \langle I_h \rangle \times 100$

* $R_{cryst} = \sum ||Fobs| - |Fcalc|| / |Fobs| \times 100$

† R_{free}, based on 10% of the total reflections

¶ RMS deviation from ideality for bonds (followed by the value for angles in parentheses)

‡ Overall average B factor (followed by average B factor of ligand in parentheses)

\# Values in parentheses for outermost shell

Materials and methods

AlkB expression and purification

E coli AlkB was expressed and purified as previously reported, with modifications.[8,9] BL21 (DE3) Rosetta T1R transformed with pNIC28-Bsa4 ΔN11 AlkB were grown in a LEX system using 750 mL of Terrific Broth (TB) supplemented with 8 g/L of glycerol and appropriate antibiotics. The culture was incubated at 37°C in the LEX system with aeration and agitation through the bubbling of filtered air through the culture. When the OD600 reached ~2, the temperature was reduced to 18°C and the cultures were induced after 30 to 60 min by addition of 0.5 mM IPTG. Growth was continued for 16-20 h and harvested by centrifugation. The cell pellets were resuspended in 1.5 times the cell pellet weight of lysis buffer containing 100 mM HEPES, 500 mM NaCl, 10 mM Imidazole, 10% glycerol, 0.5 mM TCEP, pH 8.0, Benzonase (4 uL per 750 mL cultivation) 250 U/uL from Merck, Protease Inhibitor Cocktail Set III, EDTA free (1000x dilution in lysis buffer) from Calbiochem and stored at -80°C. Cells were lysed on ice by sonication, and the lysate cleared by centrifugation and filtration. Filtered lysates were loaded onto 1 mL Ni-NTA Superflow (Qiagen) in IMAC Wash buffer 1 (20 mM HEPES, 500 mM NaCl, 10 mM imidazole, 10 % (v/v) glycerol, 0.5 mM TCEP, pH 7.5) and subsequently washed with IMAC Wash buffer 2 (20 mM HEPES, pH7.5, 500 mM NaCl, 25 mM imidazole, 10% (v/v) glycerol, 0.5 mM TCEP). Bound proteins were eluted with 500 mM imidazole and loaded onto a HiLoad 16/60 Superdex-200 column (GE Healthcare) pre-equilibrated with 20 mM HEPES, pH 7.5, 300 mM, NaCl, 10% (v/v) glycerol, 0.5 mM TCEP.

FTO and ΔN31-FTO expression and purification

The human full length FTO construct used in this study was generously provided by Dr. Giles Yeo (University of Cambridge Metabolic Research Lab). Full length FTO and ΔN31-FTO were expressed and purified as previously reported, with modifications.[1,5] BL21 (DE3) Rosetta T1R and BL21 (DE3) *E. coli* cells transformed with pNIC28-Bsa4 FTO and pET28b ΔN31-FTO respectively were grown at 37°C and 200 rpm to an OD_{600} of 0.6. Protein expression was induced by addition of 0.5 mM IPTG (Gold Biotechnology). Growth was continued at 16°C for 16 h then cells were harvested by centrifugation. The resulting cell pellet was stored at -80°C. Cell pellets were resuspended to homogeneity in 25 mM Tris,

pH7.5, 500 mM NaCl, 40 mM imidazole, pH 7.5 and 5 mM β-mercaptoethanol (β-ME). Cells were disrupted by French Press and the lysate clarified by centrifugation and filtration. FTO was purified from the crude cell lysate by Ni affinity chromatography (GE healthcare), with elution achieved by application of gradient to 500 mM imidazole. Further purification was achieved by gel filtration using a HiLoad superdex 200 26/60 (GE healthcare) in a buffer of 25 mM Tris, pH 7.5, 100 mM NaCl, 5% (v/v) glycerol and 5 mM β-ME. ΔN31-FTO was purified from the crude cell lysate by Ni affinity chromatography (GE healthcare), with elution achieved by application of gradient to 500 mM imidazole, followed by anion chromatography using a 5 mL HiTrap Q HP column (GE healthcare), with elution achieved by application of gradient to 1 M NaCl. Further purification was achieved by gel filtration using a HiLoad superdex 200 26/60 (GE healthcare) in a buffer of 25 mM Tris, pH 7.5, 50 mM NaCl and 5 mM β-ME.

Human ALKBH2 expression and purification

Human ALKBH2 was expressed and purified as previously reported, with modifications.[3] BL21 (DE3) *E. coli* cells transformed with pET28b ALKBH2 (encompassing residues 56 - 258) were grown in 37^{0}C and 200 rpm to an OD_{600} of 0.6. Protein expression was induced by addition of 0.5 mM IPTG (Gold Biotechnology). Growth was continued at 37^{0}C for 4 h then cells were harvested by centrifugation. The resulting cell pellet was stored at -80^{0}C. Cell pellets were resuspended to homogeneity in 50 mM Sodium Phosphate, pH 8, 300 mM NaCl, 10% (v/v) glycerol and 5 mM β-ME. Cells were disrupted by French Press and the lysate clarified by centrifugation and filtration. ALKBH2 was purified from the crude cell lysate by Ni affinity chromatography (GE healthcare), with elution achieved by application of gradient to 500 mM imidazole, followed by anion chromatography using a 5 mL HiTrap Heparin HP column (GE healthcare), with elution achieved by application of gradient to 1 M NaCl. Further purification was achieved by gel filtration using a HiLoad superdex 75 16/60 (GE healthcare) in a buffer of 10 mM Tris, pH 8, 100 mM NaCl and 5 mM β-ME.

ALKBH3

Human ALKBH3 (encompassing residues 1- 286, fused to a 39-amino acid His Tag at *N*-terminal) was purchased from ProSpec-Tany Technogene Ltd, Israel.

ALKBH5 expression and purification

Human ALKBH5 was expressed and purified as previously reported.[5] BL21 (DE3) Rosetta T1R transformed with pNIC28-Bsa4 ALKBH5 (encompassing residues 66-292) were grown at 37^{0}C and 200 rpm to an OD_{600} of 0.6. Protein expression was induced by addition of 0.5 mM IPTG (Gold Biotechnology). Growth was continued at 16^{0}C for 16 h then cells were harvested by centrifugation. The resulting cell pellet was stored at -80^{0}C. Cell pellets were resuspended to homogeneity in 20 mM Tris, pH 8, 500 mM NaCl, 40 mM imidazole, pH 8 and 5 mM β-ME. Cells were disrupted by French Press and the lysate clarified by centrifugation and filtration. ALKBH5 was purified from the crude cell lysate by Ni affinity chromatography (GE healthcare), with elution achieved by application of gradient to 500 mM imidazole, followed by anion chromatography using a 5 mL HiTrap Q HP column (GE healthcare), with elution achieved by application of gradient to 1 M NaCl. Further purification was achieved by gel filtration using a HiLoad superdex 75 26/60 (GE healthcare) in a buffer of 20 mM Tris, pH 8, 100 mM NaCl and 5 mM β-ME.

JMJD2A expression and purification

Human JMJD2A was expressed and purified as previously reported, with modifications.[9,10] BL21 (DE3) Rosetta T1R transformed with pNIC28-Bsa4 JMJD2A (encompassing residues 1-359) were grown in a LEX system using 750 mL of Terrific Broth (TB) supplemented with 8 g/L of glycerol and appropriate antibiotics. The culture was incubated at 37^{0}C in the LEX system with aeration and agitation through the bubbling of filtered air through the culture. When the OD600 reached ~2, the temperature was reduced to 18^{0}C, and the cultures were induced after 30 to 60 min by addition of 0.5 mM IPTG. Growth was continued for 16-20 h and harvested by centrifugation. The cell pellets were resuspended in 1.5 times the cell pellet weight of lysis buffer containing 100 mM HEPES, 500 mM NaCl, 10 mM Imidazole, 10% glycerol, 0.5 mM TCEP, pH 8.0, Benzonase (4 uL per 750 mL cultivation) 250 U/uL from Merck, Protease Inhibitor Cocktail Set III, EDTA free (1000x dilution in lysis buffer) from Calbiochem and stored at -80^{0}C. Cells were lysed on ice by sonication, and the lysate cleared by centrifugation and filtration. Filtered lysates were loaded onto 1 mL Ni-NTA Superflow (Qiagen) in IMAC Wash buffer 1 (20 mM HEPES, 500 mM NaCl, 10 mM imidazole, 10% (v/v) glycerol, 0.5 mM TCEP, pH 7.5) and subsequently washed with IMAC Wash buffer 2 (20 mM HEPES, pH7.5, 500 mM NaCl, 25 mM imidazole, 10% (v/v) glycerol, 0.5 mM TCEP). Bound proteins were eluted with 500 mM imidazole and loaded onto a HiLoad 16/60

Superdex-200 column (GE Healthcare) pre-equilibrated with 20 mM HEPES, pH 7.5, 300 mM NaCl, 10% (v/v) glycerol, 0.5 mM TCEP.

PHD2

Full length human PHD2 (fused to a MYC/DDK tag at *C*-terminal) was purchased from Origene Technology, USA.

Protein crystallography

Crystals of ΔN31-FTO in complex with **12**, **16**, and **21** were grown in hanging drops at 293K using vapour diffusion methods. The ratio of protein to reservoir solution was 2:1 (2.25 μL total drop volume). The ΔN31-FTO protein contained 8 mg/mL protein, 25 mM Tris, pH 7.5, 50 mM NaCl, 5 mM β-ME, 1 mM $NiSO_4$ and 1.5 mM ligand. The reservoir solutions for ΔN31-FTO in complex with **12**, **16**, and **21** contained 100 mM Sodium Citrate, pH 5.6, 10% w/v polyethylene glycol (PEG) 3350, 10% t-butanol. The crystals were cryocooled using well solution diluted to 22% glycerol and flash frozen in liquid nitrogen. Crystals of ΔN31-FTO in complex with **21** were screened on PXIII beamline at the Swiss Light Source. ΔN31-FTO in complex with **12** and **16** were screened on PXI beamline at the Swiss Light Source. All diffraction data were collected at 100K. All data were process with X-ray Detector Software (XDS).[11] The structures were determined by molecular replacement using Phaser-MR[12] subroutine in PHENIX[13] with 4IE5 (PDB ID)[14] as search model. Iterative rounds of model building and refinement using COOT and PHENIX were performed until the decreasing R and R_{free} no longer converged.[15]

Differential scanning fluorimetry (DSF)-based binding assay

DSF-based binding assay was performed using a MiniOpticon™ Real-Time PCR Detection System (Bio-Rad), monitoring protein unfolding using SYPRO orange (Invitrogen) according to the reported method.[16] FAM (492 nm) and ROX (610 nm) filters were used for excitation and emission respectively. Reaction mixes contained 2 μM protein, 50 μM $MnCl_2$, 100 μM compounds, 5x SYPRO orange in a final volume of 50 μl. Reagents were prepared in 50mM HEPES buffer, pH 7.5 except metals, which were dissolved as 100 mM stocks in 20 mM HCl, then further diluted in MilliQ water. Compounds tested were prepared in 100% DMSO and added such that the final concentration of DMSO was not more than 1% v/v of assay

mix. Fluorescence readings were taken every 1°C in the range 25-95°C, with the temperature increased linearly by 1°C min^{-1}. The software provided was used to perform global minimum subtraction. The inflection point, representing T_m, was calculated by fitting the Boltzmann equation to the sigmoidal curves obtained; data were processed using GraphPad Prism 6.0™. The T_m shift caused by the addition of small molecules/ fragments was determined by subtraction of the "reference" T_m (protein incubated with metal and 1% v/v DMSO) from the T_m obtained in the presence of the compound. The assay was performed in triplicate for each inhibitor, with standard deviations typically < 1°C.

HPLC-based inhibition assays for FTO

The assay was modified from previously reported method.[17] It is performed in triplicate for each inhibitor, in a final reaction volume of 25 µL. Reaction consisted of FTO (1 µM), 2OG (50 µM), $(NH_4)_2Fe(SO_4)_2 \cdot 6H_2O$ (50 µM), L-ascorbate (2 mM), 3-methylthymidine (m3T substrate, 10 µM) in 50 mM HEPES buffer, pH 7.5, and inhibitor at various concentrations (final DMSO concentration not more than 1% v/v of assay mix). The reaction was incubated at 4°C for 1 h, before analysis on a HPLC system. The substrate (m3T) and the demethylated product (thymidine) were separated using a Zorbax C18 column (4.6 mm x 250 mm) with a gradient of 95% solvent A (water + 0.1% TFA) to 60% solvent B (MeOH) over 8 min, at a flow rate of 1 mL/min at room temperature. The UV detection wavelength was set at 266 nm. The percentage inhibition was estimated based on the peak areas of the 3-methylthymidine substrate and the thymidine product. For IC_{50} determination, eight different concentrations of inhibitors were used (0, 1, 3, 10, 30, 100, 300, 1000 µM); the IC_{50s} were then calculated from the variation in percentage demethylation at different inhibitor concentrations, using nonlinear regression, with normalized dose-response fit on GraphPad Prism 6.0™.

HPLC-based assay for FTO was also performed using an m6A-ssRNA as substrate. The assay was modified from previously reported method.[21] All reaction conditions are as described for the inhibition of m3T demethylation above, except that a 15-mer ssRNA with the sequence 5'-CUUGUCA(m6A)CAGCAGA-3' (Yale University, W.M. Keck Foundation Biotechnology Resource Laboratory) was used as substrate (10 µM). The reaction was analysed on a bio-inert HPLC system. The m6A-ssRNA substrate and the corresponding demethylated product were separated using a Dionex DNAPac PA200 anion-exchange column (8 µm, 4 mm × 25 mm) with a gradient of 98% solvent A (50 mM sodium citrate buffer, pH 5.5) to 65% solvent B (50 mM sodium citrate buffer, pH 5.5 with 1.25 M sodium

chloride) over 18 min, at a flow rate of 1 mL/min at 40°C. The UV detection wavelength was set at 266 nm. The percentage inhibition was estimated based on the peak areas of the m6A-ssRNA substrate and the corresponding demethylated product. For IC_{50} determination, eight different concentrations of inhibitors were used (0, 1, 3, 10, 30, 100, 300, 1000 μM); the IC_{50s} were then calculated from the variation in percentage demethylation at different inhibitor concentrations, using nonlinear regression, with normalized dose-response fit on GraphPad Prism 6.0™.

HPLC-based inhibition assays for AlkB and ALKBH2

The assay was modified from previously reported method.[18] A 15-mer m1A-ssDNA substrate with the sequence 5'-AAAGCAG(m1A)ATTCGAA-3' (Yale University, W.M. Keck Foundation Biotechnology Resource Laboratory) was used in this inhibition assay. The assay was performed in triplicate for each inhibitor, in a final reaction volume of 25 μL. Reaction consisted of AlkB (1 μM) or ALKBH2 (2 μM), 2OG (50 μM), $(NH_4)_2Fe(SO_4)_2 \cdot 6H_2O$ (50 μM), L-ascorbate (2 mM), m1A-ssDNA substrate (10 μM), in 50 mM HEPES buffer, pH 7.5, and inhibitor at various concentrations (final DMSO concentration not more than 1% v/v of assay mix). The reaction was incubated at 4°C for 10 min, before analysis on a bio-inert HPLC system. The m1A-ssDNA substrate and the corresponding demethylated product were separated using a Dionex DNAPac PA200 anion-exchange column (8 μm, 4 mm × 25 mm) with a gradient of 98% solvent A (50 mM sodium citrate buffer, pH 5.5) to 65% solvent B (50 mM sodium citrate buffer, pH 5.5 with 1.25 M sodium chloride) over 18 min, at a flow rate of 1 mL/min at 40°C. The UV detection wavelength was set at 266 nm. The percentage inhibition was estimated based on the peak areas of the m1A-ssDNA substrate and the corresponding demethylated product. For IC_{50} determination, eight different concentrations of inhibitors were used (0, 1, 3, 10, 30, 100, 300, 1000 μM); the IC_{50s} were then calculated from the variation in percentage demethylation at different inhibitor concentrations, using nonlinear regression, with normalized dose-response fit on GraphPad Prism 6.0™.

HPLC-based inhibition assays for ALKBH5

The assay was modified from previously reported method.[5] A 5-mer m6A-ssRNA substrate with the sequence 5'-GG(m6A)CU-3' (Yale University, W.M. Keck Foundation

Biotechnology Resource Laboratory) was used in this inhibition assay. The assay was performed in triplicate for each inhibitor, in a final reaction volume of 25 μL. Reaction consisted of ALKBH5 (4 μM), 2OG (150 μM), $(NH_4)_2Fe(SO_4)_2 \cdot 6H_2O$ (150 μM), L-ascorbate (2 mM), m6A-ssRNA substrate (80 μM), in 50 mM HEPES buffer, pH 7.5, and inhibitor at various concentrations (final DMSO concentration not more than 1% v/v of assay mix). The reaction was incubated at 4°C for 30 min, before analysis on a bio-inert HPLC system. The m6A-ssRNA substrate and the corresponding demethylated product were separated using a Dionex DNAPac PA200 anion-exchange column (8 μm, 4 mm × 25 mm) with a gradient of 98% solvent A (50 mM sodium citrate buffer, pH 5.5) to 65% solvent B (50 mM sodium citrate buffer, pH 5.5 with 1 M sodium chloride) over 18 min, at a flow rate of 1 mL/min at 40°C. The UV detection wavelength was set at 266 nm. The percentage inhibition was estimated based on the peak areas of the m6A-ssRNA substrate and the corresponding demethylated product. For IC_{50} determination, eight different concentrations of inhibitors were used (0, 1, 3, 10, 30, 100, 300, 1000 μM); the IC_{50s} were then calculated from the variation in percentage demethylation at different inhibitor concentrations, using nonlinear regression, with normalized dose-response fit on GraphPad Prism 6.0™.

MALDI-based inhibition assays for ALKBH3

An 8-mer m1A-ssDNA substrate with the sequence 5'-CAG(m1A)ATTC-3' (Yale University, W.M. Keck Foundation Biotechnology Resource Laboratory) was used in this inhibition assay. The assay was performed in triplicate for each inhibitor, in a final reaction volume of 25 μL. Reaction consisted of ALKBH3 (2 μM), 2OG (150 μM), $(NH_4)_2Fe(SO_4)_2 \cdot 6H_2O$ (150 μM), L-ascorbate (2 mM), m6A-ssDNA substrate (100 μM), in 50 mM HEPES buffer, pH 7.5, and inhibitor at various concentrations (final DMSO concentration not more than 1% v/v of assay mix). The reaction was incubated at room temperature for 30 min, before 1:1 quenching with 20% v/v formic acid. 1 uL of the diluted assay mixture was then mixed with 1 uL of 3-hydroxpicolinic acid (3-HPA, the MALDI-TOF-MS matrix, Sigma-Aldrich) and spotted onto the MALDI-TOF-MS plate before analysis. The 3-HPA matrix was prepared by mixing 9 parts of 50 mg/mL 3-hydroxpicolinic acid in 50% MeCN/Milli-Q H_2O with 1 part of 50 mg/mL ammonium citrate in Milli-Q H_2O. The percentage inhibition was estimated based on the relative intensities of the methylated substrate and the demethylated product observed in the mass spectra. For IC_{50} determination, eight different concentrations of inhibitors were used (0, 1, 3, 10, 30, 100, 300, 1000 μM); the

IC_{50s} were then calculated from the variation in percentage demethylation at different inhibitor concentrations, using nonlinear regression, with normalized dose-response fit on GraphPad Prism 6.0™.

MALDI-based inhibition assays for FTO and ALKBH5

The assay was modified from previously reported method.[5] An 8-mer m6A-ssDNA substrate with the sequence 5'-TCA(m6A)CAGC-3' (Yale University, W.M. Keck Foundation Biotechnology Resource Laboratory) was used in this inhibition assay. The assay was performed in triplicate for each inhibitor, in a final reaction volume of 25 μL. Reaction consisted of FTO (2 μM) or ALKBH5 (4 μM), 2OG (150 μM), $(NH_4)_2Fe(SO_4)_2{\cdot}6H_2O$ (150 μM), L-ascorbate (2 mM), m6A-ssDNA substrate (100 μM), in 50 mM HEPES buffer, pH 7.5, and inhibitor at various concentrations (final DMSO concentration not more than 1% v/v of assay mix). The reaction was incubated at room temperature for 30 min, before 1:1 quenching with 20% v/v formic acid. 1 uL of the diluted assay mixture was then mixed with 1 uL of 3-hydroxpicolinic acid (3-HPA, the MALDI-TOF-MS matrix, Sigma-Aldrich) and spotted onto the MALDI-TOF-MS plate before analysis. The 3-HPA matrix was prepared by mixing 9 parts of 50 mg/mL 3-hydroxpicolinic acid in 50% MeCN/Milli-Q H_2O with 1 part of 50 mg/mL ammonium citrate in Milli-Q H_2O. The percentage inhibition was estimated based on the relative intensities of the methylated substrate and the demethylated product observed in the mass spectra. For IC_{50} determination, eight different concentrations of inhibitors were used (0, 1, 3, 10, 30, 100, 300, 1000 μM); the IC_{50s} were then calculated from the variation in percentage demethylation at different inhibitor concentrations, using nonlinear regression, with normalized dose-response fit on GraphPad Prism 6.0™.

MALDI-based inhibition assay for PHD2

The assay was performed as previously reported.[19] A 19-mer peptide substrate with the sequence DLDLEMLAPYIPMDDDFQL (GL Biochem (Shanghai) Ltd) was used in this inhibition assay. The assay was performed in triplicate for each inhibitor, in a final reaction volume of 25 μL. Reaction consisted of PHD2 (4 μM), 2OG (150 μM), $(NH_4)_2Fe(SO_4)_2{\cdot}6H_2O$ (150 μM), L-ascorbate (2 mM), peptide substrate (100 μM), in 50 mM HEPES buffer, pH 7.5, and inhibitor at various concentrations (final DMSO concentration not more than 1% v/v of assay mix). The reaction was incubated at room temperature for 30

min, before 1:1 quenching with methanol. 1 uL of the diluted assay mixture was then mixed with 1 uL of α-cyano-4-hydroxycinnamic acid (CHCA, the MALDI-TOF-MS matrix, Sigma-Aldrich) and spotted onto the MALDI-TOF-MS plate before analysis. The CHCA matrix consisted of 10 mg/mL CHCA in 50% MeCN/Milli-Q H_2O containing 0.1% TFA. The percentage inhibition was estimated based on the relative intensities of the unhydroxylated substrate and the hydroxylated product observed in the mass spectra. For IC_{50} determination, eight different concentrations of inhibitors were used (0, 1, 3, 10, 30, 100, 300, 1000 μM); the IC_{50s} were then calculated from the variation in percentage hydroxylation at different inhibitor concentrations, using nonlinear regression, with normalized dose-response fit on GraphPad Prism 6.0™.

MALDI-based inhibition assay for JMJD2A

The assay was performed as previously reported, with modifications.[20] A peptide substrate with the sequence ARK(me3)STGGK (GL Biochem (Shanghai) Ltd) was used in this inhibition assay. The assay was performed in triplicate for each inhibitor, in a final reaction volume of 25 μL. Reaction consisted of JMJD2A (2 μM), 2OG (50 μM), $(NH_4)_2Fe(SO_4)_2\cdot 6H_2O$ (100 μM), L-ascorbate (2 mM), peptide substrate ARK(me3)STGGK (10 μM), in 50 mM HEPES buffer, pH 7.5, and inhibitor at various concentrations (final DMSO concentration not more than 1% v/v of assay mix). The reaction was incubated at room temperature for 30 min, before 1:1 quenching with methanol followed by addition of four volumes of 20 mM triammonium citrate. 1 uL of the diluted assay mixture was then mixed with 1 uL of α-cyano-4-hydroxycinnamic acid (CHCA, the MALDI-TOF-MS matrix, Sigma-Aldrich) and spotted onto the MALDI-TOF-MS plate before analysis. The CHCA matrix consisted of 10 mg/mL CHCA in 50% MeCN/Milli-Q H_2O containing 0.1% TFA. The percentage inhibition was estimated based on the relative intensities of the methylated substrate and the demethylated products observed in the mass spectra. For IC_{50} determination, eight different concentrations of inhibitors were used (0, 1, 3, 10, 30, 100, 300, 1000 μM); the IC_{50s} were then calculated from the variation in percentage demethylation at different inhibitor concentrations, using nonlinear regression, with normalized dose-response fit on GraphPad Prism 6.0™.

Kinetics of FTO inhibition by 12

To determine the mode of inhibition of FTO by **12** with respect to m3T substrate, various concentrations of m3T (5 μM, 10 μM, 25 μM and 50 μM) were incubated with FTO (1 μM) in the presence of 0 μM, 2 μM, or 5 μM of **12**. Reactions were adjusted to ensure that less than 20% of the substrate was consumed, and were analysis on a HPLC system. The substrate (m3T) and the demethylated product (thymidine) were separated using a Zorbax C18 column (4.6 mm x 250 mm) with a gradient of 95% solvent A (water + 0.1% TFA) to 60% solvent B (MeOH) over 8 min, at a flow rate of 1 mL/min at room temperature. The UV detection wavelength was set at 266 nm. The kinetics parameters for FTO were determined using Lineweaver-Burk plot and Dixon plot on GraphPad Prism 6.0™. Similar reaction conditions were used to determine the mode of inhibition of FTO by **12** with respect to co-substrate 2OG, where varying concentrations of 2OG (5 μM, 10 μM, 25 μM and 50 μM) were incubated with FTO (1 μM) in the presence of 0 μM, 2 μM, or 5 μM of **12**.

MTT cell viability assay

Human HeLa cells were seeded in 96-well plates at a density of $1x10^4$ cells/well, and allowed to grow for 24 h before being exposed to varying concentrations of compound **25** (0, 5, 10, 50 and 100 μM). Cells that were treated with 1% v/v DMSO served as negative control. After incubation at 37°C for 24 h, the supernatant was removed, and each well was washed twice with PBS. 20 μl of MTT (3-(4,5-dimethylthiazol-2-yl)-2,5-diphenyltetrazolium bromide) solution (5 mg/ml in PBS) and 100μl of medium were then introduced, and the solution was incubated at 37°C for another 4 h. The resultant formazan crystals were dissolved in 100 μL DMSO and its concentration is determined by measuring its absorbance intensity at 570 nm using a microplate reader. A background wavelength of 630 nm was used. Cell viability was expressed as a percentage relative to the untreated control cells. All experiments were performed in triplicate.

Determination of intracellular hydrolysis of 25

HeLa cells were treated with 50 μM **25** or 50 μM **12**. After 6 h of incubation at 37°C, the media was aspirated and the cells were washed with PBS. The number of cells in each sample was determined. The cells were then lysed and the compound extracted from the cells using 80% aqueous methanol. All samples were shaken for 10 min on a vortex mixer and then centrifuged for 10 min at 15000 rpm. The resulting supernatant was diluted with water in

proportion to cell count, and analysed by reverse phase HPLC using a Zorbax C18 column (4.6 mm x 250 mm) with a gradient of 95% solvent A (water + 0.1% TFA) to 60% solvent B (MeOH) over 8 min, at a flow rate of 1 mL/min at room temperature. The UV detection wavelength was set at 254 nm. The percentage hydrolysis was estimated based on the peak areas of ethyl ester **25** and the hydrolysis product **12**. The compounds were identified by comparing their retention times with that of standards.

Cell-based inhibition assay of compound 25

The assay was performed as previously reported.[21] Human HeLa cells were treated with either 10 μM or 50 μM of compound **25** or with 1% v/v DMSO (negative control) and allowed to incubation at 37°C for 6 h. After which, the cells were harvested and the total RNA isolated with TRAZOL Reagent (Invitrogen). The mRNA was extracted using PolyATtract® mRNA Isolation Systems (Promega), and the rRNA contaminants were removed using RiboMinus Transcriptome Isolation Kit (Invitrogen). The concentration of the resultant mRNA was measured using NanoDrop.

To digest the mRNA to nucleosides, 1 μg of mRNA was treated with 2 Units of nuclease P1 in 40 μL of buffer containing 25 mM of NaCl, and 2.5 mM of $ZnCl_2$ at 37ºC for 1 hour, followed by the addition of 1M NH_4HCO_3 (3 μL), and alkaline phosphatase (0.5 Unit). After incubation at 37ºC for 3 h, the solution was diluted 3 times, and analyzed on a HPLC system. The nucleosides were separated using a Zorbax C18 column (4.6 mm x 250 mm) with a gradient of 95% solvent A (water + 0.1% TFA) to 60% solvent B (MeOH) over 8 min, at a flow rate of 1mL/min at room temperature. The UV detection wavelength was set at 266 nm. The concentrations of 6-methyladenine (m6A) and adenine (A) were determined based on their peak areas, using calibration plots obtained from pure nucleoside standards. The percentage of m6A/A was then obtained using the calculated concentrations.

Chemical synthesis

All reagents, solvents, and starting materials were obtained from commercial suppliers Aldrich, Alfa Aesar or Acros. Reactions were monitored by TLC, which was performed on precoated aluminum-backed plates (Merck, silica 60 F254). Column chromatography was conducted on silica gel (230-400 mesh). Melting points were determined using a Leica Galen III hot-stage melting point apparatus and microscope. Infrared spectra were recorded from Nujol mulls between potassium bromide discs, on a Perkin Elmer FT-IR spectrometer. NMR spectra were acquired using a Bruker AVC500 NMR spectrometer, in DMSO-d_6, or $CDCl_3$, using TMS as the internal standard. Chemical shifts (δ) were reported in parts per million downfield from the internal standard. The signals were quoted as s (singlet), d (doublet), t (triplet), m (multiplet), (br) br. Coupling constants *J* are given in Hz (± 0.5 Hz). ESI-MS spectra were recorded on a Finnigan MAT LC-MS. High resolution mass spectra (HRMS) were recorded using Bruker MicroTOF. The purity of all compound synthesized were >95% as determined by analytical reverse-phase HPLC (Ultimate 3000). The synthesis of **4**[22], **5**[23], **6**[24], **7**[22], **8**[22], **9**[22], **10**[22], **11**[22], **21**[22], **23**[22], **NOG**[25], **31**[26] and **36**[26] were reported.

Synthesis of compounds 12-19

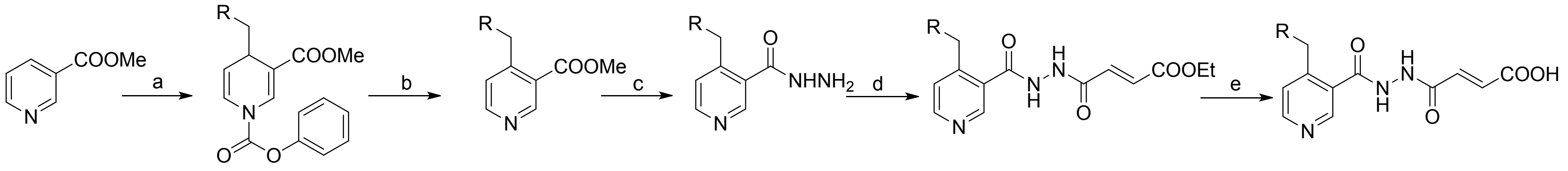

**30**	**31** R = phenyl	**36** R = phenyl	**41** R = phenyl	**25** R = phenyl	**12** R = phenyl
	**32** R = *para tert*-butyl phenyl	**37** R = *para tert*-butyl phenyl	**42** R = *para tert*-butyl phenyl	**47** R = *para tert*-butyl phenyl	**16** R = *para tert*-butyl phenyl
	**33** R = *para* CF_3 phenyl	**38** R = *para* CF_3 phenyl	**43** R = *para* CF_3 phenyl	**48** R = *para* CF_3 phenyl	**17** R = *para* CF_3 phenyl
	**34** R = 2-naphthyl	**39** R = 2-naphthyl	**44** R = 2-naphthyl	**49** R = 2-naphthyl	**18** R = 2-naphthyl
	**35** R = 1-naphthyl	**40** R = 1-naphthyl	**45** R = 1-naphthyl	**50** R = 1-naphthyl	**19** R = 1-naphthyl

Reagents and conditions: (a) phenyl chloroformate, substituted benzyl bromides, zinc dust, Tetrahydrofuran, 0°C; (b) sulphur, decalin, reflux; (c) hydrazine monohydrate, EtOH, reflux; (d) ethyl pentafluorophenyl fumarate, Tetrahydrofuran : EtOAc = 1 : 1, room temperature; (e) lithium hydroxide monohydrate, Tetrahydrofuran : H_2O = 2 : 1, room temperature.

Synthesis of compound 13

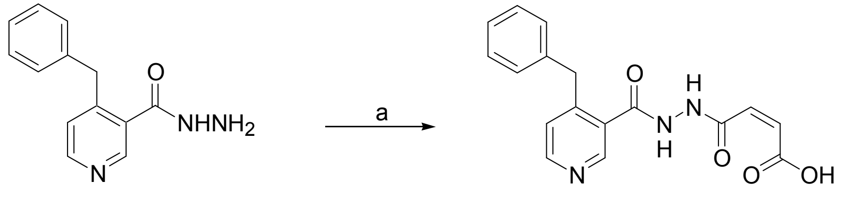

41 13

Reagents and conditions: (a) maleic anhydride, EtOAc, reflux.

Synthesis of compound 14

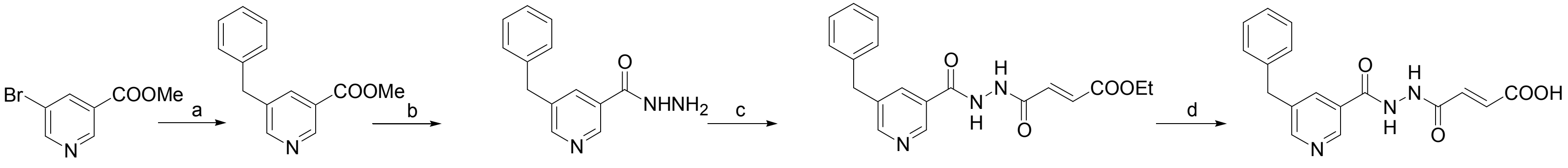

51 52 53 54 14

Reagents and conditions: (a) LiCl, $Pd(PPh_3)_4$, substituted benzyl bromides, indium powder, DMF, 100°C; (b) hydrazine monohydrate, EtOH, reflux; (c) ethyl pentafluorophenyl fumarate, Tetrahydrofuran : EtOAc = 1 : 1, room temperature; (d) lithium hydroxide monohydrate, Tetrahydrofuran : H_2O = 2 : 1, room temperature.

Synthesis of compound 15

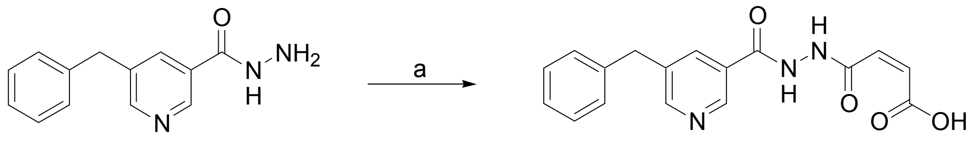

53 15

Reagents and conditions: (a) maleic anhydride, EtOAc, reflux.

Synthesis of compounds 20

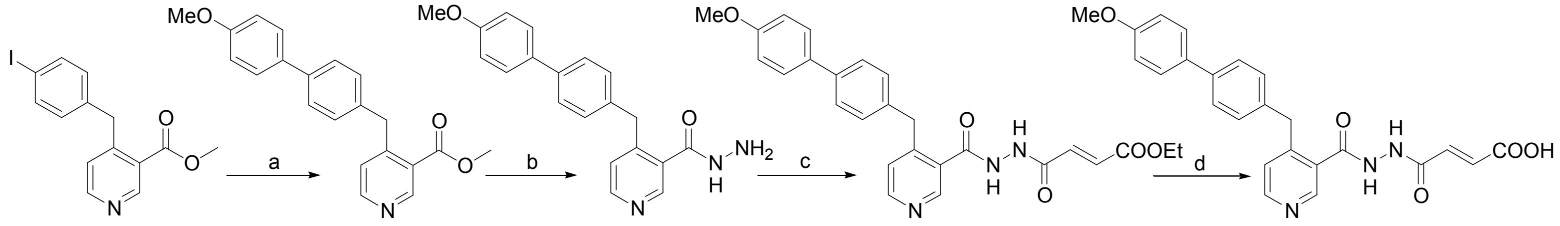

55 56 57 58 20

Reagents and conditions: (a) 4-methoxyphenylboronic acid, $Pd(PPh_3)_4$, potassium carbonate, DMF, 100°C; (b) hydrazine monohydrate, EtOH, reflux; (c) ethyl pentafluorophenyl fumarate, Tetrahydrofuran : EtOAc = 1 : 1, room temperature; (d) lithium hydroxide monohydrate, Tetrahydrofuran : H_2O = 2 : 1, room temperature.

Synthesis of compound 22

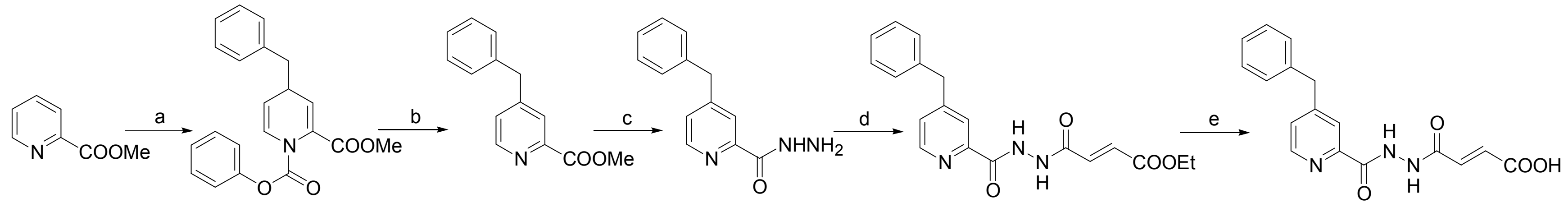

59 60 61 62 63 22

Reagents and conditions: (a) phenyl chloroformate, benzyl bromide, zinc dust, Tetrahydrofuran, 0°C; (b) sulphur, decalin, reflux; (c) hydrazine monohydrate, EtOH, reflux; (d) ethyl pentafluorophenyl fumarate, Tetrahydrofuran : EtOAc = 1 : 1, room temperature; (e) lithium hydroxide monohydrate, Tetrahydrofuran : H_2O = 2 : 1, room temperature.

4-(2-Benzoylhydrazinyl)-4-oxobutanoic acid (4)

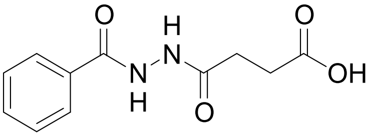

Compound **4** was synthesized as reported.[22] To a stirred solution of benzhydrazide (136 mg, 1.0 mmol) in EtOAc (10 mL) was added a solution of succinic anhydride (100 mg, 1.0 mmol) in EtOAc (5 mL). The mixture was allowed to stand at room temperature for 24 h. The precipitate was collected and recrystallised from EtOH to afford 193 mg (81%) product as a white solid. m.p. 169-172°C; IR(KBr disk) νmax/cm^{-1}: 3194 (br), 1724, 1674, 1614, 1575, 1483, 1211; ^{1}H-NMR (500 MHz, DMSO-d_6) δ 2.46-2.49 (4H, m, CH_2CH_2), 7.48-7.51 (2H, m, 2×ArCH), 7.56-7.59 (1H, m, ArCH), 7.86-7.88 (2H, m, 2×ArCH), 9.93 (1H, br s, NH), 10.32 (1H, br s, NH), 12.15 (1H, br s, COOH) ppm; ^{13}C-NMR (125 MHz, DMSO-d_6) δ 27.9 (CH_2), 28.8 (CH_2), 127.4 (ArCH), 128.4 (ArCH), 131.8 (ArCH), 132.5 (ArC), 165.4 (ArCO), 170.5 (CO), 173.6 (CO) ppm; HRMS (ESI$^-$) calcd. for $C_{11}H_{11}N_2O_4$ (M-H$^+$), 235.0713; found, 235.0715.

Benzoylhydrazide of maleic acid (6)

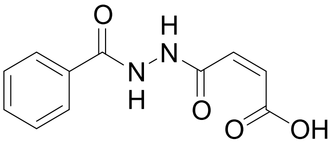

Compound **6** was synthesized as reported.[24] To a solution of phenyl carboxylic acid hydrazide (1.36 g, 10 mmol) in 50 ml EtOAc was added a solution of maleic anhydride (0.98 g, 10 mmol) in 30 ml EtOAc. The reaction mixture was allowed to stand for 2 h. The precipitate was separated by filtration and then recrystallized from EtOH to give 2.25 g (96%) product. m.p. 178-179°C; IR(KBr disk) νmax/cm^{-1}: 3280 (OH), 3200 (NH), 1710 (CO), 1658 (CO); ^{1}H-NMR (60 MHz, DMSO-d_6) δ 6,45 (2H, s, CH=CH), 7.28-8.27 (5H, m, C_6H_5), 10.68 (2H, s, br. 2NH).

4-Oxo-4-(2-(chinoline-3-carbonyl)hydrazinyl)butanoic acid (7)

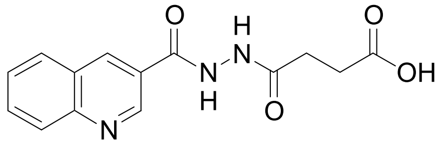

Compound **7** was synthesized as reported.[22] A suspension of chinoline-3-carbohydrazide (37 mg, 0.2 mmol) in EtOAc (10 mL) was heated to 70 °C to dissolve the starting material. Then, a solution of succinic anhydride (20 mg, 0.2 mmol) in EtOAc (2 mL) was added and the reaction mixture was stirred at 70 °C for 1 h, at 50 °C for 3 h and finally at room temperature for 3 h. Solids were collected and washed with EtOAc to afford 42 mg (74%) of **7** as a white solid. m.p. 208-211ºC; IR (KBr disk) νmax/cm^{-1}: 3438 (bs), 3197, 1728, 1611, 1594, 1491; ^{1}H-NMR (500 MHz, DMSO-d_6) δ 2.47-2.51 (4H, m, 2×CH_2), 7.72 (1H, dd, J=7.0 Hz, 7.0 Hz, ArCH), 7.90 (1H, dd, J=7.0 Hz, 7.0 Hz, ArCH), 8.09-8.14 (2H, m, 2×ArCH), 8.88 (1H, d, J=2.0 Hz, ArCH), 9.28 (1H, d, J=2.0 Hz, ArCH), 10.11 (1H, br s, NH), 10.72 (1H, br s, NH), 12.11 (1H, br s, COOH) ppm; ^{13}C-NMR (125 MHz, DMSO-d_6) δ 28.1 (CH_2), 28.8 (CH_2), 125.3 (ArC), 126.4 (ArC), 127.6 (ArCH), 128.8 (ArCH), 129.2 (ArCH), 131.7 (ArCH), 136.1 (ArCH), 148.6 (ArC), 148.6 (ArCH), 164.1 (CO), 170.5 (CO), 173.6 (COOH) ppm; HRMS (ESI$^+$) calcd. for $C_{14}H_{13}N_3NaO_4$ (M+Na^+), 310.0798; found, 310.0803.

(*E*)- 4-Oxo-4-(2-(quinoline-3-carbonyl)hydrazinyl)but-2-enoic acid (8)

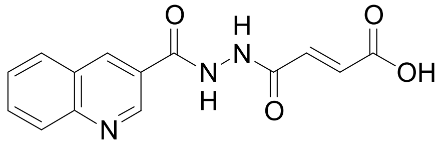

Compound **8** was synthesized as reported.[22] (*E*)-Ethyl 4-oxo-4-(2-(quinoline-3-carbonyl)hydrazinyl)but-2-enoate (90 mg, 0.3 mmol) was added to a stirring solution of LiOH.H2O (38 mg, 0.9mmol) in H_2O:THF (1:2) (15 ml) at room temperature. TLC analysis (EtOAc: MeOH, 4:1) indicated complete consumption of starting material (Rf 0.5) after 3 h. The reaction mixture was then washed with EtOAc (3 x 10 mL). The aqueous layer was acidified with 1 N HCl to pH 3, and the white precipitate formed was filtered, washed with H_2O, and dried under vacuo to give the product as a yellow solid 50 mg (63%). m.p. > 330ºC; IR (KBr disk) 3450 (br), 3186, 1698, 1607, 1487 cm^{-1}; ^{1}H-NMR (500 MHz, DMSO-d_6) δ 6.70 (1H, d, J= 15.5 Hz, =C-H), 7.08 (1H, d, J = 15.5 Hz, =C-H), 7.75 (1H, dd, J 7.5, 7.6 Hz, ArCH), 7.93 (1H, dd, J 7.5,

7.6, ArCH), 8.12-8.17 (2H, m, 2 x ArCH) 8.93 (1H, d, J 2.0, ArCH), 9.31 (1H, d, J 2.0, ArCH), 10.79 (H, bs, NH), 11.06 (H, bs, NH); ^{13}C-NMR (125 MHz, DMSO-d_6) δ 125.0 (ArC), 126.4 (ArC), 127.7 (ArCH), 128.6 (ArCH), 129.3 (ArCH), 131.3 (=CH), 131.7 (ArCH), 134.1 (=CH), 136.5 (ArCH), 148.5 (ArCH), 148.6 (ArC) 162.4 (C=O), 163.9 (C=O), 166.1 (COOH); MS (ESI$^-$) m/z 284.1 [M-H$^+$]; HMRS (ESI$^-$) calcd. for $C_{14}H_{12}N_3O_4$ [M-H$^+$] 286.0822; found 286.0822.

(*Z*)-4-Oxo-4-(2-(chinoline-3-carbonyl)hydrazinyl)but-2-enoic acid (9)

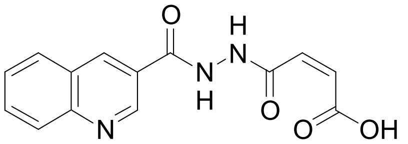

Compound **9** was synthesized as reported.[22] A suspension of chinoline-3-carbohydrazide (37 mg, 0.2 mmol) in EtOAc (10 mL) was heated to 70 °C to dissolve the starting material. Then, a solution of maleic anhydride (20 mg, 0.2 mmol) in EtOAc (2 mL) was added and the reaction mixture was stirred at 70 °C for 4 h. The mixture was cooled to room temperature, solids were collected and washed with EtOAc to afford 53 mg (94%) of **9** as a white solid. m.p. 188-191ºC; IR (KBr disk) vmax/cm^{-1}: 3241 (bs), 1683, 1646, 1541, 1285; ^{1}H-NMR (500 MHz, DMSO-d_6) δ 6.37 (1H, d, J=12.0 Hz, olefinic CH), 6.45 (1H, d, J=12.0 Hz, olefinic CH), 7.73 (1H, dd, J=7.5 Hz, 7.5 Hz, ArCH), 7.91 (1H, dd, J=7.5 Hz 7.5 Hz, ArCH), 8.10-8.15 (2H, m, 2×ArCH), 8.91 (1H, d, J=2.0 Hz, ArCH), 9.30 (1H, d, J=2.0 Hz, ArCH), 10.72 (1H, br s, NH), 10.99 (1H, br s, NH), 13.14 (1H, br s, COOH) ppm; ^{13}C-NMR (125 MHz, DMSO-d_6) δ125.1 (ArC), 126.4 (ArC), 126.9 (olefinic CH), 127.6 (ArCH), 127.6 (ArCH), 128.8 (ArCH), 129.3 (ArCH), 131.6 (ArCH), 132.9 (olefinic CH), 136.3 (ArCH), 148.6 (ArCH), 148.7 (ArC), 163.1 (CO), 163.9 (CO), 167.0 (COOH) ppm; HRMS (ESI$^-$) calcd. for $C_{14}H_{10}N_3O_4$ (M-H$^+$), 284.0677; found, 284.0671.

(*E*)-4-(2-(Isochinoline-3-carbonyl)hydrazinyl)-4-oxobut-2-enoic acid (10)

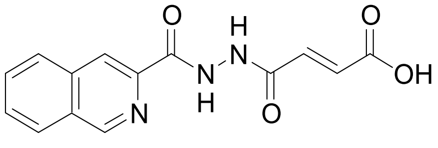

Compound **10** was synthesized as reported.[22] (*E*)-Ethyl 4-(2-(isochinoline-3-carbonyl)hydrazinyl)-4-oxobut-2-enoate (150 mg, 0.5 mmol) was added to a stirred solution of LiOH·H_2O (63 mg, 1.5 mmol) in H_2O:THF (1:2 v/v) (15 ml) at room

temperature. TLC analysis (EtOAc: MeOH, 4:1) indicated complete consumption of starting material (Rf 0.5) after 3 h. The reaction mixture was then washed with EtOAc (3×10 mL). The aqueous layer was acidified with to pH 3 with 1 N HCl and precipitates were filtered off, washed with water and dried in vacuo to afford 120 mg (88%) of **10** as a yellow solid. m.p. >330°C; IR (KBr disk) νmax/cm^{-1}: 3209, 1714, 1605, 1486, 1300, 1186; ^{1}H-NMR (500 MHz, DMSO-d$_6$) δ 6.67 (1H, d, J=15.5 Hz, olefinic CH), 7.05 (1H, d, J=15.5 Hz, olefinic CH), 7.84-7.87 (1H, m, ArCH), 7.90-7.93 (1H, m, ArCH), 8.24 (1H, d, J=8.0 Hz, ArCH), 8.29 (1H, d, J=8.0 Hz, ArCH), 8.61 (1H, s, ArCH), 9.43 (1H, s, ArCH), 10.85 (1H, br s, NH), 10.89 (1H, br s, NH); ^{13}C-NMR (125 MHz, DMSO-d$_6$) δ120.5 (ArCH), 127.9 (ArCH), 128.1 (ArCH), 129.4 (ArC), 129.5 (ArCH), 131.1 (olefinic CH), 131.6 (ArCH), 134.3 (olefinic CH), 135.2 (ArC), 142.8 (ArC), 151.9 (ArCH) 161.7 (C=O), 162.5 (C=O), 164.8 (COOH); HRMS (ESI$^-$) calcd. for $C_{14}H_{10}N_3O_4$ (M-H$^+$), 284.0677; found 284.0670.

(*E*)-4-(2-(9*H*-Pyrido[3,4-b]indole-3-carbonyl)hydrazinyl)-4-oxobut-2-enoic acid (11)

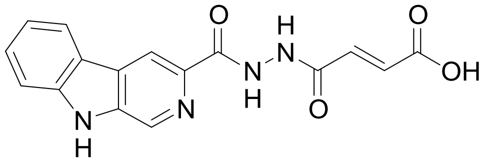

Compound **11** was synthesized as reported.[22] (*E*)-Ethyl 4-(2-(9H-pyrido[3,4-b]indole-3carbonyl)hydrazinyl)-4-oxobut-2-enoate (340 mg, 1.0 mmol) was added to a stirred solution of LiOH·H_2O (125 mg, 3.0 mmol) in H_2O:THF (1:2 v/v) (30 ml) at room temperature. TLC analysis (EtOAc:MeOH, 4:1 v/v) indicated complete consumption of starting material (Rf 0.5) after 3 h. The reaction mixture was then washed with EtOAc (3×10 mL). The aqueous layer was acidified to pH 3with 1 N HCl and the obtained precipitate was washed with water and dried in vacuo to afford 240 mg (76%) of **11** as a yellow solid. m.p. >330°C; IR (KBr disk) νmax/cm^{-1}: 3425 (br), 1688, 1611, 1493, 1458; ^{1}H-NMR (500 MHz, DMSO-d$_6$) δ 6.67 (1H, d, J=15.5 Hz, olefinic CH), 7.10 (1H, d, J=15.5 Hz, olefinic CH), 7.33 (1H, dd, J=7.5 Hz, 8.0 Hz, ArCH), 7.62 (1H, dd, J=7.5 Hz, 8.0 Hz, ArCH), 7.69 (1H, d, J=8.0 Hz, ArCH), 8.44 (1H, d, J=8.0 Hz, ArCH), 8.89 (1H, s, ArCH), 8.95 (1H, s, ArCH), 10.62 (1H, d, J=2.0 Hz, NH), 10.91 (1H, d, J=2.0 Hz, NH), 12.09 (1H, br s, indole NH); ^{13}C-NMR (125 MHz, DMSO-d$_6$) δ 112.3 (ArCH), 114.8 (ArCH), 120.2 (ArCH), 120.9 (ArC),

122.4 (ArCH), 128.0 (ArC), 128.8 (ArCH), 130.9 (olefinic CH), 132.7 (ArCH), 134.5 (olefinic CH), 137.4 (ArC), 138.1 (ArC), 141.0 (ArC) 161.5 (C=O), 163.0 (C=O), 166.1 (COOH); HRMS (ESI$^-$) calcd. for $C_{18}H_{16}N_4O_4Na$ (M-H$^+$), 323.0786; found, 323.0794.

4-[*N'*-(4-Benzyl-pyridine-3-carbonyl)-hydrazino]-4-oxo-but-2-enoic acid (12)

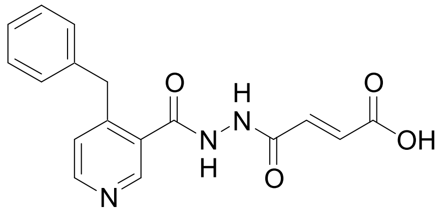

Lithium hydroxide monohydrate (36 mg, 0.84 mmol) in 4 ml water was added to a stirred solution of **25** (100 mg, 0.28 mmol) in 8 ml Tetrahydrofuran at room temperature. The reaction mixture was stirred at room temperature for 3 h. The reaction mixture was then washed by EtOAc (3×10 ml), and the aqueous layer was acidified by 2 N HCl to pH = 3. The precipitate was collected to give 80 mg product as white solid. Yield 88%. m.p. 223 – 224°C. MS (ESI, negative ion) [M-H]$^-$ 323.9. IR (KBr disk) υ/cm^{-1}: 3180 (NH), 3027 (NH), 1705 (CO acid), 1587 (CO amide), 1563 (CO amide). ^{1}H NMR (400 MHz, DMSO-d$_6$) δ 4.17 (2H, s, benzyl CH$_2$), 6.69 (1H, d, J = 15.6 Hz, CH), 7.04 (1H, d, J = 15.6 Hz, CH), 7.19 – 7.31 (5H, m, CH), 8.55 (1H, d, J = 5.2 Hz, CH), 8.59 (1H, s, CH), 10.73 (1H, s, NH), 10.75 (1H, s, NH). ^{13}C NMR (100 MHz, DMSO-d$_6$) δ 36.9 (benzyl CH$_2$), 124.9 (CH), 126.3 (CH), 128.5 (C2'', C6''), 129.0 (C3'', C5''), 130.1 (C4''), 131.3 (C1''), 134.0 (C4'), 139.2 (C2'), 147.8 (C3'), 148.7 (C5'), 150.9 (C1'), 162.2 (CO), 165.6 (CO), 166.0 (CO). HRMS (ESI, negative ion) $C_{17}H_{14}N_3O_4$ [M-H]$^-$ requires 324.0984; Found 324.0986.

4-[*N'*-(4-Benzyl-pyridine-3-carbonyl)-hydrazino]-4-oxo-but-2-enoic acid (13)

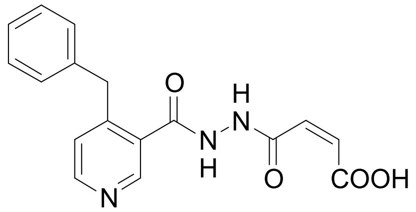

To a solution of **41** (113 mg, 0.5 mmol) in 10 ml EtOAc was added maleic anhydride (50 mg, 0.5 mmol). The reaction mixture was refluxed for 2 h. Upon cooling, the precipitate was filtered and washed with EtOAc to give 80 mg product as pale yellow

solid. Yield, 49%. m.p. 184 – 186°C. MS (ESI, negative ion) [M-H]$^-$ 323.9. IR (KBr disk) υ/cm^{-1}: 3188 (NH), 3026 (NH), 1717 (CO acid), 1608 (CO amide), 1592 (CO amide). ^{1}H NMR (400 MHz, DMSO-d$_6$) δ 4.15 (2H, s, benzyl CH$_2$), 6.33 (1H, d, *J* = 15 Hz, CH), 6.43 (1H, d, *J* = 15 Hz, CH), 7.20 – 7.22 (1H, m, CH), 7.25 (1H, d, *J* = 5.2 Hz, CH), 7.28 – 7.29 (4H, m, CH), 8.53 (1H, d, *J* = 5.2 Hz, CH), 8.57 (1H, s, CH), 10.66 (1H, br, NH), 10.72 (1H, s, NH). ^{13}C NMR (100 MHz, DMSO-d$_6$) δ 36.9 (benzyl CH$_2$), 124.9 (CH), 126.3 (CH), 126.8 (C4''), 128.4 (C2'', C6''), 129.0 (C3'', C5''), 130.1 (C1''), 132.9 (C4'), 139.2 (C2'), 147.8 (C3'), 148.6 (C5'), 150.8 (C1'), 163.0 (CO), 165.6 (CO), 166.9 (CO). HRMS (ESI, negative ion) $C_{17}H_{14}N_3O_4$ [M-H]$^-$ requires 324.0984; Found 324.0991.

4-[*N'*-(5-Benzyl-pyridine-3-carbonyl)-hydrazino]-4-oxo-but-2-enoic acid (14)

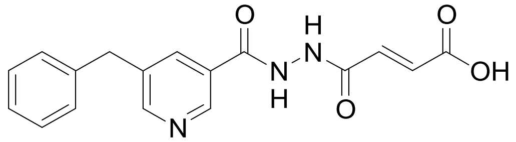

Lithium hydroxide monohydrate (36 mg, 0.84 mmol) in 3 ml water was added to a stirred solution of **54** (100 mg, 0.28 mmol) in 6 ml Tetrahydrofuran at room temperature. The reaction mixture was stirred at room temperature for 3 h. The reaction mixture was then washed by EtOAc (3×10 ml), and the aqueous layer was acidified by 2 N HCl to pH = 3. The precipitate was collected to give 60mg product as white solid. Yield 66%. m.p. 269 – 270°C. MS (ESI, negative ion) [M-H]$^-$ 323.9. IR (KBr disk) υ/cm^{-1}: 3167 (NH), 3029 (NH), 1692 (CO acid), 1599 (CO amide), 1585 (CO amide). ^{1}H NMR (400 MHz, DMSO-d$_6$) δ 4.06 (2H, s, benzyl CH$_2$), 6.65 (1H, d, *J* = 15.6 Hz, CH), 7.02 (1H, d, *J* = 15.6 Hz, CH), 7.20 – 7.34 (5H, m, CH), 8.05 (1H, t, *J* = 2.0Hz, CH), 8.70 (1H, d, *J* = 2.0 Hz, CH), 8.87 (1H, d, *J* = 2.0 Hz, CH), 10.66 (1H, s, NH), 10.83 (1H, s, NH). ^{13}C NMR (100 MHz, DMSO-d$_6$) δ 37.7 (benzyl CH$_2$), 126.3 (CH), 127.7 (CH), 128.6 (C2'', C6''), 128.7 (C3'', C5''), 131.2 (C4''), 134.0 (C1''), 135.1 (C4'), 136.9 (C2'), 139.9 (C3'), 146.1 (C5'), 152.7 (C1'), 162.2 (CO), 163.8 (CO), 166.0 (CO). HRMS (ESI, negative ion) $C_{17}H_{14}N_3O_4$ [M-H]$^-$ requires 324.0984; Found 324.0980.

4-[*N'*-(5-Benzyl-pyridine-3-carbonyl)-hydrazino]-4-oxo-but-2-enoic acid (15)

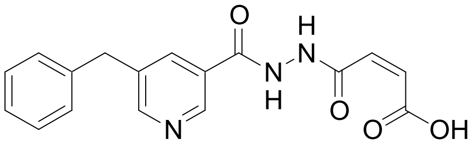

To a solution of **53** (75 mg, 0.33 mmol) in 5 ml EtOAc was added maleic anhydride (33 mg, 0.33 mmol). The reaction mixture was refluxed for 2 h. Upon cooling, the precipitate was filtered and washed with EtOAc to give 90 mg product as pale green solid. Yield, 83%. m.p. 159 – 161°C. MS (ESI, negative ion) $[M-H]^-$ 323.9. ^1H NMR (400 MHz, DMSO-d_6) δ 4.05 (2H, s, benzyl CH_2), 6.32 (1H, d, *J* = 15 Hz, CH), 6.42 (1H, d, *J* = 15 Hz, CH), 7.19 – 7.24 (1H, m, CH), 7.27 – 7.34 (4H, m, CH), 8.06 (1H, t, *J* = 2.0 Hz, CH), 8.70 (1H, d, *J* = 2.0 Hz, CH), 8.87 (1H, d, *J* = 2.0 Hz, CH), 10.60 (1H, br, NH), 10.80 (1H, s, NH). ^{13}C NMR (100 MHz, DMSO-d_6) δ 37.7 (benzyl CH_2), 126.3 (CH), 126.8 (CH), 127.7 (C4’’), 128.6 (C2’’, C6’’), 128.7 (C3’’, C5’’), 132.8 (C1’’), 135.2 (C4’), 136.8 (C2’), 139.9 (C3’), 146.1 (C5’), 152.6 (C1’), 163.1 (CO), 163.8 (CO), 166.9 (CO). HRMS (ESI, negative ion) $C_{17}H_{14}N_3O_4$ $[M-H]^-$ requires 324.0984; Found 324.0991.

4-{*N'*-[4-(4-tert-Butyl-benzyl)-pyridine-3-carbonyl]-hydrazino}-4-oxo-but-2-enoic acid (16)

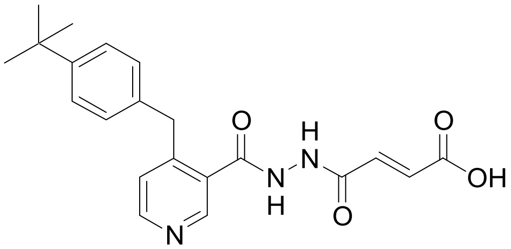

Lithium hydroxide monohydrate (16 mg, 0.375 mmol) in 2 ml water was added to a stirred solution of **47** (51 mg, 0.125 mmol) in 4 ml Tetrahydrofuran at room temperature. The reaction mixture was stirred at room temperature for 3 h. The reaction mixture was then washed by EtOAc (3×10 ml), and the aqueous layer was acidified by 2 N HCl to pH = 3. The precipitate was collected to give 20 mg product as white solid. Yield 42%. m.p. 267 – 268°C. MS (ESI, negative ion) $[M-H]^-$ 380.1. IR (KBr disk) υ/cm^{-1}: 3180 (NH), 3024 (NH), 1702 (CO acid), 1607 (CO amide), 1590 (CO amide). ^1H NMR (400 MHz, DMSO-d_6) δ 1.24 (9H, s, CH_3), 4.11 (2H, s, benzyl CH_2), 6.68 (1H, d, *J* = 15.2 Hz, CH), 7.03 (1H, d, *J* = 15.2 Hz, CH), 7.21 - 7.31 (5H, m, CH), 8.53 (1H, d, *J* = 5.2 Hz, CH), 8.57 (1H, s, CH), 10.73 (1H, s, NH),

10.74 (1H, s, NH). ^{13}C NMR (100 MHz, DMSO-d$_6$) δ 31.1 (C(*C*H$_3$)$_3$) 34.0 (*C*(CH$_3$)$_3$) 36.4 (benzyl CH$_2$), 124.9 (CH), 125.2 (C3'', C5''), 128.6 (C2'', C6''), 130.0 (CH), 131.3 (C1''), 134.0 (C4''), 136.2 (C4'), 147.8 (C2'), 148.6 (C3'), 148.8 (C5'), 150.8 (C1'), 162.2 (CO), 165.7 (CO), 166.0 (CO). HRMS (ESI, negative ion) $C_{21}H_{22}N_3O_4$ [M-H]$^-$ requires 380.1610; Found 380.1613.

4-Oxo-4-{*N'*-[4-(4-trifluoromethyl-benzyl)-pyridine-3-carbonyl]-hydrazino}-but-2-enoic acid (17)

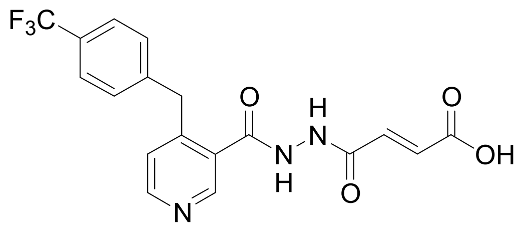

Lithium hydroxide monohydrate (31 mg, 0.72 mmol) in 3 ml water was added to a stirred solution of **48** (100 mg, 0.24 mmol) in 6 ml Tetrahydrofuran at room temperature. The reaction mixture was stirred at room temperature for 3 h. The reaction mixture was then washed by EtOAc (3×10 ml), and the aqueous layer was acidified by 2 N HCl to pH = 3. The precipitate was collected to give 70 mg product as white solid. Yield 74%. m.p. 235 – 237°C. MS (ESI, negative ion) [M-H]$^-$ 391.9. IR (KBr disk) υ/cm^{-1}: 3170 (NH), 3028 (NH), 1706 (CO acid), 1638 (CO amide), 1594 (CO amide). ^{1}H NMR (400 MHz, DMSO-d$_6$) δ 4.31 (2H, s, benzyl CH$_2$), 6.68 (1H, d, J = 15.2 Hz, CH), 7.03 (1H, d, J = 15.2 Hz, CH), 7.43 (1H, d, J = 5.2 Hz, CH), 7.53 (2H, d, J = 8.0 Hz, CH), 7.65 (2H, d, J = 8.0 Hz, CH), 8.62 (1H, d, J = 5.2 Hz, CH), 8.68 (1H, s, CH), 10.79 (1H, s, NH), 10.85 (1H, s, NH). ^{13}C NMR (100 MHz, DMSO-d$_6$) δ 36.8 (benzyl CH$_2$), 125.3 (CH), 125.4 (CH), 125.8 (C1''), 129.9 (C2'', C6'', C3'', C5''), 130.6 (C4''), 131.4 (C4'), 133.9 (C2'), 143.7 (CF$_3$), 146.5 (C3'), 149.5 (C5'), 149.9 (C1'), 162.2 (CO), 164.8 (CO), 166.0 (CO). HRMS (ESI, negative ion) $C_{18}H_{13}F_3N_3O_4$ [M-H]$^-$ requires 392.0858; Found 392.0852.

4-[*N'*-(4-Naphthalen-2-ylmethyl-pyridine-3-carbonyl)-hydrazino]-4-oxo-but-2-enoic acid (18)

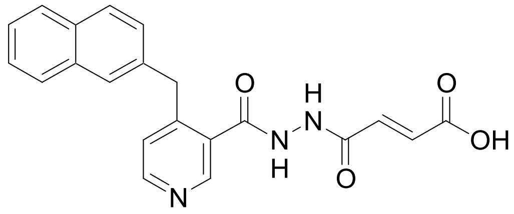

Lithium hydroxide monohydrate (13 mg, 0.3 mmol) in 1.5 ml water was added to a stirred solution of **49** (40 mg, 0.1 mmol) in 3 ml Tetrahydrofuran at room temperature. The reaction mixture was stirred at room temperature for 3 h. The reaction mixture was then washed by EtOAc (3×10 ml), and the aqueous layer was acidified by 2 N HCl to pH = 3. The precipitate was collected to give 15 mg product as pale yellow solid. Yield 40%. m.p. 223 – 224°C. MS (ESI, negative ion) $[M-H]^-$ 373.9. IR (KBr disk) υ/cm^{-1}: 3171 (NH), 3022 (NH), 1698 (CO acid), 1590 (CO amide). ^{1}H NMR (400 MHz, DMSO-d_6) δ 4.34 (2H, s, benzyl CH_2), 6.70 (1H, d, J = 15.6 Hz, CH), 7.04 (1H, d, J = 15.6 Hz, CH), 7.33 (1H, d, J = 4.8 Hz, CH), 7.44 – 7.51 (3H, m, CH), 7.83 – 7.87 (4H, m, CH), 8.54 (1H, d, J = 4.8 Hz, CH), 8.61 (1H, s, CH), 10.76 (1H, s, NH), 10.80 (1H, s, NH). ^{13}C NMR (100 MHz, DMSO-d_6) δ 37.0 (benzyl CH_2), 125.5 (CH), 126.0 (CH), 127.2 (C3''), 127.3 (C2''), 127.4 (C1''), 127.4 (C4''), 127.5 (C5''), 127.9 (C10''), 128.0 (C6''), 131.3 (C9''), 131.6 (C7''), 133.0 (C8''), 134.0 (C4'), 136.8 (C2'), 147.9 (C3'), 148.5 (C5'), 150.9 (C1'), 162.2 (CO), 165.7 (CO), 166.0 (CO). HRMS (ESI, negative ion) $C_{21}H_{16}N_3O_4$ $[M-H]^-$ requires 374.1141; Found 374.1144.

4-[*N'*-(4-Naphthalen-1-ylmethyl-pyridine-3-carbonyl)-hydrazino]-4-oxo-but-2-enoic acid (**19**)

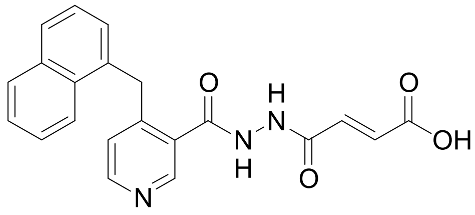

Lithium hydroxide monohydrate (33 mg, 0.75 mmol) in 2.5 ml water was added to a stirred solution of **50** (100 mg, 0.25 mmol) in 5 ml Tetrahydrofuran at room temperature. The reaction mixture was stirred at room temperature for 3 h. The reaction mixture was then washed by EtOAc (3×10 ml), and the aqueous layer was

acidified by 2 N HCl to pH = 3. The precipitate was collected to give 68 mg product as pale yellow solid. Yield 73%. m.p. 253 – 255°C. MS (ESI, negative ion) $[M-H]^-$ 373.9. IR (KBr disk) υ/cm^{-1}: 3168 (NH), 3019 (NH), 1702 (CO acid), 1604 (CO amide), 1583 (CO amide). ^{1}H NMR (400 MHz, DMSO-d_6) δ 4.64 (2H, s, benzyl CH_2), 6.68 (1H, d, J = 15.6 Hz, CH), 6.88 (1H, d, J = 4.8 Hz, CH), 7.03 (1H, d, J = 15.6 Hz, CH), 7.41 (1H, d, J = 6.8 Hz, CH), 7.47 – 7.53 (3H, m, CH), 7.87 (1H, d, J = 8.0 Hz, CH), 7.94 – 7.98 (2H, m, CH), 8.44 (1H, d, J = 4.8 Hz, CH), 8.66 (1H, s, CH), 10.79 (1H, s, NH), 10.87 (1H, s, NH). ^{13}C NMR (100 MHz, DMSO-d_6) δ 34.3 (benzyl CH_2), 123.9 (CH), 124.0 (CH), 125.6 (C2''), 125.8 (C3''), 126.3 (C10''), 127.4 (C5''), 127.9 (C4''), 128.6 (C1''), 130.1 (C9''), 131.3 (C6''), 131.5 (C8''), 133.5 (C7''), 133.9 (C4'), 134.4 (C2'), 147.7 (C3'), 148.3 (C5'), 151.0 (C1'), 162.2 (CO), 165.8 (CO), 166.0 (CO). HRMS (ESI, negative ion) $C_{21}H_{16}N_3O_4$ $[M-H]^-$ requires 374.1141; Found 374.1144.

4-{*N'*-[4-(4'-Methoxy-biphenyl-4-ylmethyl)-pyridine-3-carbonyl]-hydrazino}-4-oxo-but-2-enoic acid (20)

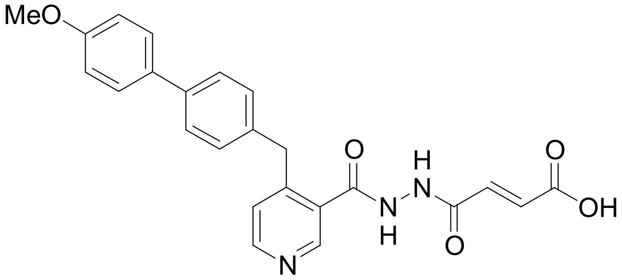

Lithium hydroxide monohydrate (14 mg, 0.33 mmol) in 2 ml water was added to a stirred solution of **58** (50 mg, 0.11 mmol) in 4 ml Tetrahydrofuran at room temperature. The reaction mixture was stirred at room temperature for 3 h. The reaction mixture was then washed by EtOAc (3×5 ml), and the aqueous layer was acidified by 2 N HCl to pH = 3. The precipitate was collected to give 15 mg product as colourless crystals. Yield 32%. m.p. 251 – 253°C. MS (ESI, negative ion) $[M-H]^-$ 323.8. IR (KBr disk) υ/cm^{-1}: 3302 (NH), 3197 (NH), 1689 (CO ester), 1605 (CO amide), 1558 (CO amide). ^{1}H NMR (400 MHz, DMSO-d_6) δ 3.78 (3H, s, OCH_3) 4.18 (2H, s, benzyl CH_2), 6.69 (1H, d, J = 15.6 Hz, CH), 7.00 (2H, d, J = 8.8 Hz, CH), 7.03 (1H, d, J = 15.6 Hz, CH), 7.32 (1H, d, J = 4.8 Hz, CH), 7.35 (2H, d, J = 8.0 Hz, CH), 7.52 (2H, d, J = 8.0 Hz, CH), 7.56 (2H, d, J = 8.8 Hz, CH), 8.55 (1H, d, J = 4.8 Hz, CH), 8.60 (1H, s, CH), 10.74 (1H, s, NH), 10.76 (1H, s, NH). ^{13}C NMR (100 MHz,

DMSO-d_6) δ 36.5 (benzyl CH_2), 55.1 (OCH_3), 114.3 (C3''', C5'''), 125.0 (CH), 126.2 (CH), 126.3 (C2''', C6'''), 127.6 (C2'', C6''), 129.5 (C3'', C5''), 131.4 (C1'''), 132.2 (C4''), 134.0 (C1''), 137.7 (C4'), 137.9 (C2'), 147.9 (C3'), 148.7 (C5'), 150.9 (C1'), 158.8 (C4'''), 162.3 (CO), 165.7 (CO), 166.1 (CO). HRMS (ESI, negative ion) $C_{24}H_{20}N_3O_5$ $[M-H]^-$ requires 430.1403; Found 430.1420.

(*E*)-4-(2-Nicolinoylhydrazinyl)-4-oxobut-2-enoic acid (21)

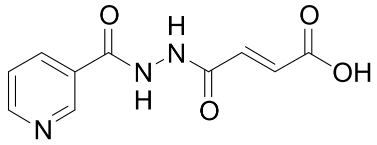

Compound **21** was synthesized as reported.[22] (*E*)-Ethyl 4-(2-nicolinoylhydrazinyl)-4-oxobut-2-enoate (150 mg, 0.57 mmol) was added to a stirred solution of LiOH·H_2O (75 mg, 1.8 mmol) in H_2O:THF (1:2 v/v) (15 ml) at room temperature. TLC analysis (EtOAc:MeOH, 4:1 v/v) indicated complete consumption of starting material (Rf 0.6) after 3 h. The reaction mixture was then washed with EtOAc (3×10 mL). The aqueous layer was acidified with to pH 3 with 1 N HCl. The obtained precipitate was filtered off, washed with water, and dried under vacuo to afford 87 mg (65%) of 9 as a white solid. m.p. 274-276°C; IR (KBr disk) νmax/cm^{-1}: 3400-3100 (br), 3206, 3076, 1702, 1619, 1596, 1153; ^{1}H-NMR (500 MHz, DMSO-d_6) δ 6.68 (1H, d, J=15.5 Hz, olefinic CH), 7.05 (1H, d, J=15.5 Hz, olefinic CH), 7.68-7.71 (1H, m, ArCH), 8.36-8.40 (1H, m, ArCH), 8.83-8.85 (1H, m, ArCH), 9.10 (1H, d, J=2.0 Hz, ArCH), 10.76 (1H, br s, NH), 10.99 (1H,br s, NH); ^{13}C-NMR (125 MHz, DMSO-d_6) δ 124.3 (ArCH), 128.5 (ArC), 131.4 (olefinic CH), 134.1 (olefinic CH), 137.0 (ArCH), 147.2 (ArCH), 151.1 (ArCH), 162.3 (C=O), 163.3 (C=O), 166.1 (COOH); HRMS (ESI^-) calcd. for $C_{10}H_8N_3O_4$ ($M-H^+$), 234.0520; found, 234.0514.

4-[*N'*-(4-Benzyl-pyridine-2-carbonyl)-hydrazino]-4-oxo-but-2-enoic acid (22)

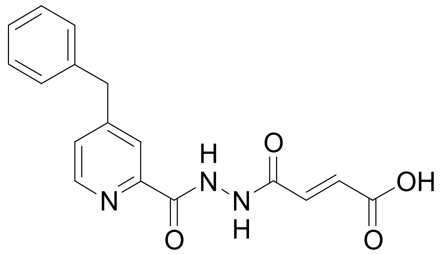

Lithium hydroxide monohydrate (22 mg, 0.51 mmol) in 1.5 ml water was added to a stirred solution of **63** (60 mg, 0.17 mmol) in 3 ml Tetrahydrofuran at room temperature. The reaction mixture was stirred at room temperature for 3 h. The reaction mixture was then washed by EtOAc (3×5 ml), and the aqueous layer was acidified by 2 N HCl to pH = 3. The precipitate was collected to give 40 mg product as white solid. Yield 72%. m.p. = 248 – 249°C. MS (ESI, negative ion) $[M-H]^-$ 323.8. IR (KBr disk) υ/cm^{-1}: 3343 (NH), 3240 (NH), 1718 (CO acid), 1647 (CO amide), 1599 (CO amide). ^{1}H NMR (400 MHz, DMSO-d_6) δ 4.10 (2H, s, benzyl CH_2), 6.63 (1H, d, J = 15.6 Hz, CH), 7.01 (1H, d, J = 15.6 Hz, CH), 7.22 – 7.35 (5H, m, CH), 7.52 – 7.53 (1H, m, CH), 7.88 (1H, s, CH), 8.57 (1H, d, J = 4.8 Hz, CH), 10.67 (1H, s, NH), 10.72 (1H, s, NH). ^{13}C NMR (100 MHz, DMSO-d_6) δ 40.0 (benzyl CH_2), 122.4 (CH), 126.5 (CH), 127.1 (C4''), 128.6 (C2'', C6''), 128.9 (C3'', C5''), 131.0 (C1''), 134.2 (C4'), 139.1 (C2'), 148.7 (C3'), 148.9 (C5'), 152.2 (C1'), 161.7 (CO), 162.3 (CO), 166.0 (CO). HRMS (ESI, negative ion) $C_{17}H_{14}N_3O_4$ $[M-H]^-$ requires 324.0984; Found 324.0975.

(*E*)-4-Oxo-4-(2-picolinoylhydrazinyl)but-2-enoic acid (23)

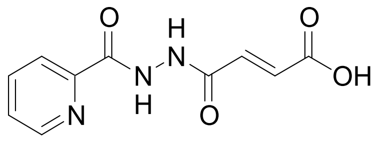

Compound **23** was synthesized as reported.[22] (*E*)-Ethyl 4-oxo-4-(2-picolinoylhydrazinyl)but-2-enoate (150 mg, 0.6 mmol) was added to a solution of LiOH·H_2O (75 mg, 1.8 mmol) in water:THF (1:2) (15 ml) and the mixture was stirred at room temperature. TLC analysis (EtOAc: MeOH, 4:1) indicated complete consumption of starting material (Rf 0.6) after 3 h. The reaction mixture was then washed with EtOAc (3×10 mL). The aqueous layer was acidified to pH 3 with 1 N HCl. A white precipitate was obtained, filtered off, washed with water and dried in vacuo to afford 130 mg (98%) of **23** as a white solid. m.p. 272-275 °C; IR (KBr disk)

vmax/cm^{-1}: 3400-3100 (br), 3317, 3213, 1724, 1630, 1153; ^{1}H-NMR (500 MHz, DMSO-d_6) δ 6.66 (1H, d, J=15.5 Hz, olefinic CH), 7.04 (1H, d, J=15.5 Hz, olefinic CH), 7.65-7.68 (1H, m, ArCH), 8.02-8.06 (2H, m, 2×ArCH), 8.69-8.70 (1H, m, ArCH), 10.73 (1H, br s, NH), 10.75 (1H, br s, NH); ^{13}C-NMR (125 MHz, DMSO-d_6) δ 122.4 (ArCH), 127.1 (ArCH), 131.2 (olefinic CH), 134.2 (olefinic CH), 138.0 (ArCH), 148.7 (ArCH), 148.8 (ArC), 161.8 (C=O), 162.4 (C=O), 166.1 (COOH); HRMS (ESI^-) calcd. for $C_{10}H_8N_3O_4$ ($M-H^+$) 234.0520; found 234.0512.

***N*-oxalylglycine (NOG)**

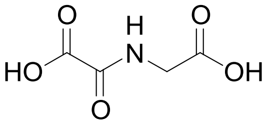

NOG was synthesized as reported.[25] A stirred solution of 25 (24.5 g, 140 mmol) in water (467 mL) was treated under argon at room temperature with aqueous sodium hydroxide (1 M, 308 mL, 308 mmol). The reaction was maintained at room temperature for 2 h and then percolated during 20 min through a column of Amberlite IR-l20(H) [600 mL, 1140 mequiv?; previously washed with water (2 L)]. The column was eluted with water until the pH of the eluent had risen to 4. The eluent was evaporated to give an oil (17.3 g) that solidified on standing. The solid was dissolved in the minimum volume of boiling ethyl acetate, diluted with an equal volume of dichloromethane, cooled rapidly, and then filtered through cotton wool to remove some greasy residues. The crystals that formed from the filtrate were washed with dichloromethane and dried at room temperature over P_4O_{10} under vacuum to give 11.65 g (57%) **NOG**. m.p. 113-115 °C; ^{1}H NMR (DMSO-d_6) δ 3.80 (2H, d, J = 6 Hz, CH_2), 8.97 (1H, t, J = 6 Hz, NH); ^{13}C NMR (D_2O) δ 41.5, 160.5, 162.1, 172.5.

4-Benzyl-4*H*-pyridine-1,3-dicarboxylic acid 3-methyl ester 1-phenyl ester (31) [24]

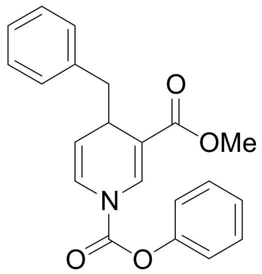

Phenyl chloroformate (1 g, 6.5 mmol) was added dropwise to a solution of **30** (0.89 g, 6.5 mmol) in 15 ml dry Tetrahydrofuran at 0°C. The reaction mixture was stirred for 1 h in an ice bath. Benzyl zinc bromide (6 mmol) in 15 ml dry Tetrahydrofuran (prepared by treating benzyl bromide with zinc dust in dry Tetrahydrofuran at room temperature) was then added dropwise to the cold phenoxycarbonyl pyridinium chloride solution. The reaction mixture was stirred for 2 h in an ice bath. The reaction was then quenched by 20 ml saturated NH_4Cl solution. The reaction mixture was extracted with EtOAc (3 × 20 ml), and the extract was washed with saturated $NaHCO_3$, saturated NH_4Cl, and brine. The extract was concentrated in *vacuo*, and the residue was purified by column chromatography (Hexane : EtOAc = 6 : 1) to give 500 mg **31** as yellow oil. Yield 24%. MS (ESI, positive ion) $[M+H]^+$ 350.0. ^1H NMR (400 MHz, $CDCl_3$) δ 2.59 (1H, dd, J_1 = 12 Hz, J_2 = 8Hz, benzyl CH_2), 2.97 (1H, dd, J_1 = 12 Hz, J_2 = 4Hz, benzyl CH_2), 3.53 – 3.57 (1H, m, CH), 3.72 (3H, s, OCH_3), 5.02 (1H, s, br, CH), 6.77 (1H, d, J = 8 Hz, CH), 7.05 – 7.19 (7H, m, CH), 7.30 – 7.34 (3H, m, CH), 7.95 (1H, s, br, CH).

4-(4-*tert*-Butyl-benzyl)-4*H*-pyridine-1,3-dicarboxylic acid 3-methyl ester 1-phenyl ester (32)

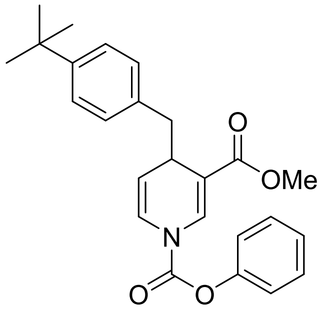

Phenyl chloroformate (0.74 g, 4.8 mmol) was added dropwise to a solution of **30** (0.66 g, 4.8 mmol) in 15ml dry Tetrahydrofuran at 0°C. The reaction mixture was stirred for 1 h in an ice bath. 4–*tert*-Butyl benzyl zinc bromide (4.4 mmol) in 15 ml dry Tetrahydrofuran (prepared by treating 4–*tert*-butyl benzyl bromide with zinc dust in dry Tetrahydrofuran at room temperature) was then added dropwise to the cold phenoxycarbonyl pyridinium chloride solution. The reaction mixture was stirred for 2 h in an ice bath. The reaction was then quenched by 20 ml saturated NH_4Cl solution. The reaction mixture was extracted with EtOAc (3 × 20 ml), and the extract was washed with saturated $NaHCO_3$, saturated NH_4Cl, and brine. The extract was concentrated in *vacuo*, and the residue was purified by column chromatography (Hexane : EtOAc = 6 : 1) to give 480 mg **32** as yellow oil. Yield 50%. MS (ESI, positive ion) $[M+H]^+$ 406.1. ^1H NMR (400 MHz, $CDCl_3$) δ 1.31 (9H, s, $C(CH_3)_3$), 2.60 (1H, dd, J_1 = 12.8 Hz, J_2 = 9.2Hz, benzyl CH_2), 3.03 (1H, dd, J_1 = 12.8 Hz, J_2 = 3.6Hz, benzyl CH_2), 3.57 – 3.61 (1H, m, CH), 3.79 (3H, s, OCH_3), 5.12 (1H, s, br, CH), 6.85 (1H, d, J = 6 Hz, CH), 7.12 – 7.15 (3H, m, CH), 7.26 – 7.32 (4H, m, CH), 7.38 – 7.42 (2H, m, CH), 8.04 (1H, s, br, CH).

4-(4-Trifluoromethyl-benzyl)-4*H*-pyridine-1,3-dicarboxylic acid 3-methyl ester 1-phenyl ester (33)

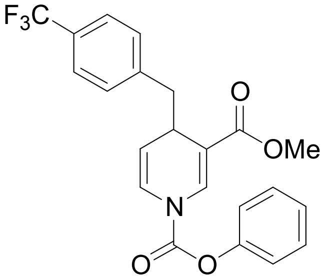

Phenyl chloroformate (1 g, 6.5 mmol) was added dropwise to a solution of **30** (0.89 g, 6.5 mmol) in 15 ml dry Tetrahydrofuran at 0°C. The reaction mixture was stirred for 1 h in an ice bath. 4–trifluoromethyl benzyl zinc bromide (5 mmol) in 15 ml dry Tetrahydrofuran (prepared by treating 4–trifluoromethyl benzyl bromide with zinc dust in dry Tetrahydrofuran at room temperature) was then added dropwise to the cold phenoxycarbonyl pyridinium chloride solution. The reaction mixture was stirred for 2 h in an ice bath. The reaction was then quenched by 20 ml saturated NH_4Cl solution. The reaction mixture was extracted with EtOAc (3 × 20 ml), and the extract was washed with saturated $NaHCO_3$, saturated NH_4Cl, and brine. The extract was concentrated in *vacuo*, and the residue was purified by column chromatography (Hexane : EtOAc = 6 : 1) to give 1.7 g **33** as white solid. Yield 82%. MS (ESI, positive ion) $[M+H]^+$ 417.9. ^1H NMR (400 MHz, $CDCl_3$) δ 2.76 (1H, dd, J_1 = 12.8 Hz, J_2 = 8.4 Hz, benzyl CH_2), 3.08 (1H, dd, J_1 = 12.8 Hz, J_2 = 3.2 Hz, benzyl CH_2), 3.64 – 3.68 (1H, m, CH), 3.81 (3H, s, OCH_3), 5.07 (1H, s, br, CH), 6.87 (1H, d, J = 8 Hz, CH), 7.11 – 7.13 (2H, m, CH), 7.28 – 7.31 (3H, m, CH), 7.39 – 7.42 (2H, m, CH), 7.55 (2H, d, J = 8 Hz, CH), 8.03 (1H, s, CH).

4-Naphthalen-2-ylmethyl-4*H*-pyridine-1,3-dicarboxylic acid 3-methyl ester 1-phenyl ester (34)

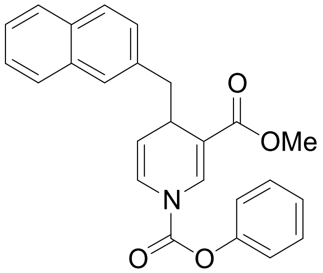

Phenyl chloroformate (1 g, 6.5 mmol) was added dropwise to a solution of **30** (0.89 g, 6.5 mmol) in 15 ml dry Tetrahydrofuran at 0°C. The reaction mixture was stirred for 1 h in an ice bath. 2–naphthyl zinc bromide (5 mmol) in 15 ml dry Tetrahydrofuran (prepared by treating 2–(bromomethyl)naphthalene with zinc dust in dry Tetrahydrofuran at room temperature) was then added dropwise to the cold phenoxycarbonyl pyridinium chloride solution. The reaction mixture was stirred for 2 h in an ice bath. The reaction was then quenched by 20 ml saturated NH_4Cl solution. The reaction mixture was extracted with EtOAc (3 × 20 ml), and the extract was washed with saturated $NaHCO_3$, saturated NH_4Cl, and brine. The extract was concentrated in *vacuo*, and the residue was purified by column chromatography (Hexane : EtOAc = 6 : 1) to give 0.75 mg **34** as yellow oil. Yield 38%. MS (ESI, positive ion) $[M+H]^+$ 400.2. ^1H NMR (400 MHz, $CDCl_3$) δ 2.81 (1H, dd, J_1 = 12.8 Hz, J_2 = 9.2 Hz, benzyl CH_2), 3.23 (1H, dd, J_1 = 12.8 Hz, J_2 = 3.2 Hz, benzyl CH_2), 3.69 – 3.74 (1H, m, CH), 3.80 (3H, s, OCH_3), 5.11 (1H, s, br, CH), 6.84 (1H, d, J = 8.8 Hz, CH), 7.19 – 7.46 (9H, m, CH), 7.76 – 7.82 (3H, m, CH), 8.04 (1H, s, CH).

4-Naphthalen-1-ylmethyl-4*H*-pyridine-1,3-dicarboxylic acid 3-methyl ester 1-phenyl ester (35)

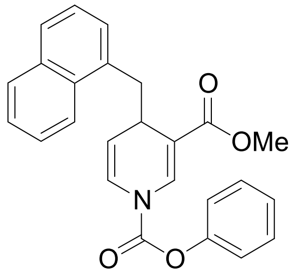

Phenyl chloroformate (1.65 g, 10.7 mmol) was added dropwise to a solution of **30** (1.5 g, 10.7 mmol) in 15 ml dry Tetrahydrofuran at 0°C. The reaction mixture was stirred for 1 h in an ice bath. 1–naphthyl zinc bromide (8.2 mmol) in 15 ml dry

Tetrahydrofuran (prepared by treating 1–(bromomethyl)naphthalene with zinc dust in dry Tetrahydrofuran at room temperature) was then added dropwise to the cold phenoxycarbonyl pyridinium chloride solution. The reaction mixture was stirred for 2 h in an ice bath. The reaction was then quenched by 20 ml saturated NH_4Cl solution. The reaction mixture was extracted with EtOAc (3 × 20 ml), and the extract was washed with saturated $NaHCO_3$, saturated NH_4Cl, and brine. The extract was concentrated in *vacuo*, and the residue was purified by column chromatography (Hexane : EtOAc = 6 : 1) to give 1.4 g **35** as yellow oil. Yield 43%. MS (ESI, positive ion) $[M+H]^+$ 400.2. ^1H NMR (400 MHz, $CDCl_3$) δ 2.85 – 2.91 (1H, m, benzyl CH_2), 3.42 – 3.51 (1H, m, benzyl CH_2), 3.79 – 3.81 (1H, m, CH), 3.83 (3H, s, OCH_3), 4.95 (1H, s, br, CH), 6.86 (1H, d, *J* = 8.8 Hz, CH), 7.16 – 7.28 (9H, m, CH), 7.37 – 7.42 (3H, m, CH), 8.13 (1H, s, CH).

4-Benzyl-nicotinic acid methyl ester (36)[26]

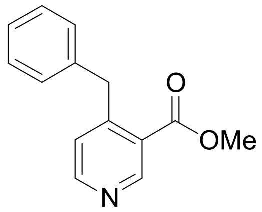

A solution of **31** (500 mg, 1.44 mmol) and sulphur (46 mg, 1.44 mmol) in 3 ml decalin was heated to reflux for 5 h. The reaction mixture was cooled down to the room temperature and extracted by 1 N HCl (4 × 4 ml). The aqueous extract was basified with 2 N NaOH to pH = 8 and extracted by DCM (3 × 10 ml). The DCM was removed in *vacuo* to give 200 mg **36** as rose red oil. Yield 62%. MS (ESI, positive ion) $[M+H]^+$ 228.0. ^1H NMR (400 MHz, $CDCl_3$) δ 3.90 (3H, s, OCH_3), 4.40 (2H, s, benzyl CH_2), 7.09 (1H, d, *J* = 5.2 Hz, CH), 7.15 (2H, d, *J* = 7.2 Hz, CH), 7.24 (1H, d, *J* = 7.2 Hz, CH), 7.30 (2H, t, *J* = 7.2 Hz, CH), 8.57 (1H, d, *J* = 4 Hz, CH), 9.09 (1H, s, CH).

4-(4-*tert*-Butyl-benzyl)-nicotinic acid methyl ester (37)

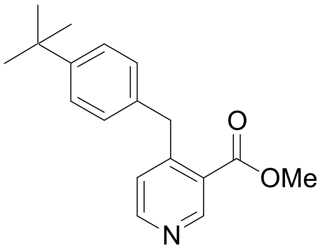

A solution of **32** (0.892 mg, 2.2 mmol) and sulphur (71 mg, 2.2 mmol) in 6 ml decalin was heated to reflux for 5 h. The reaction mixture was cooled down to the room temperature and extracted by 1 N HCl (4 × 4 ml). The aqueous extract was basified with 2 N NaOH to pH = 8 and extracted by DCM (3 × 10 ml). The DCM was removed in *vacuo* to give 200 mg **37** as brown oil. Yield 78%. MS (ESI, positive ion) $[M+H]^+$ 284.3. ^1H NMR (400 MHz, $CDCl_3$) δ 1.31 (9H, s, $C(CH_3)_3$), 3.92 (3H, s, OCH_3), 4.38 (2H, s, benzyl CH_2), 7.08 (2H, d, J = 8.4 Hz, CH), 7.15 (1H, d, J = 3.6 Hz, CH), 7.33 (2H, d, J = 8.4 Hz, CH), 8.59 (1H, s, br, CH), 9.11 (1H, s, CH).

4-(4-Trifluoromethyl-benzyl)-nicotinic acid methyl ester (38)

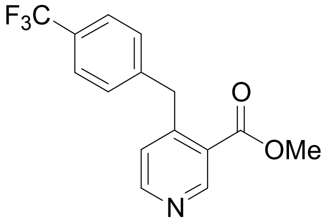

A solution of **33** (0.99 g, 2.44 mmol) and sulphur (76 mg, 2.44 mmol) in 6 ml decalin was heated to reflux for 5 h. The reaction mixture was cooled down to the room temperature and extracted by 1 N HCl (4 × 4 ml). The aqueous extract was basified with 2 N NaOH to pH = 8 and extracted by DCM (3 × 10 ml). The DCM was removed in *vacuo* to give 0.47 g **38** as brown oil. Yield 67%. MS (ESI, positive ion) $[M+H]^+$ 296.1. ^1H NMR (400 MHz, $CDCl_3$) δ 3.91 (3H, s, OCH_3), 4.48 (2H, s, benzyl CH_2), 7.14 (1H, d, J = 5.2 Hz, CH), 7.28 (2H, d, J = 8.0 Hz, CH), 7.56 (2H, d, J = 8 Hz, CH), 8.64 (1H, s, br, CH), 9.15 (1H, s, CH).

4-Naphthalen-2-ylmethyl-nicotinic acid methyl ester (39)

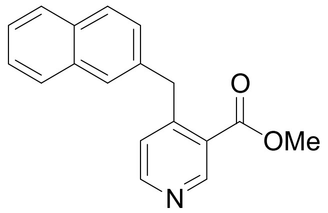

A solution of **34** (700 mg, 1.75 mmol) and sulphur (56 mg, 1.75 mmol) in 5 ml decalin was heated to reflux for 5 h. The reaction mixture was cooled down to the room temperature and extracted by 1 N HCl (4 × 4 ml). The aqueous extract was basified with 2 N NaOH to pH = 8 and extracted by DCM (3 × 10 ml). The DCM was removed in *vacuo* to give 220 mg **39** as brown oil. Yield 45%. MS (ESI, positive ion) $[M+H]^+$ 278.0. ^1H NMR (400 MHz, $CDCl_3$) δ 3.90 (3H, s, OCH_3), 4.59 (2H, s, benzyl CH_2), 7.17 (1H, d, J = 4.0 Hz, CH), 7.27 (1H, dd, J_1 = 8.4 Hz, J_2 = 1.6 Hz, CH), 7.43 – 7.48 (2H, m, CH), 7.57 (1H, s, CH), 7.74 – 7.82 (3H, m, CH), 8.56 (1H, s, CH), 9.13 (1H, s, CH).

4-Naphthalen-1-ylmethyl-nicotinic acid methyl ester (40)

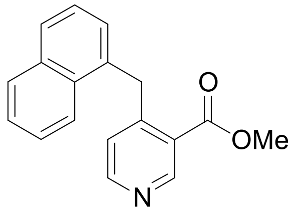

A solution of **35** (1.4 g, 3.25 mmol) and sulphur (112 mg, 3.5 mmol) in 10 ml decalin was heated to reflux for 5 h. The reaction mixture was cooled down to the room temperature and extracted by 1 N HCl (4 × 4 ml). The aqueous extract was basified with 2 N NaOH to pH = 8 and extracted by DCM (3 × 10 ml). The DCM was removed in *vacuo* to give 200 mg **40** as brown oil. Yield 21%. MS (ESI, positive ion) $[M+H]^+$ 278.1. ^1H NMR (400 MHz, $CDCl_3$) δ 3.94 (3H, s, OCH_3), 4.86 (2H, s, benzyl CH_2), 6.78 (1H, d, J = 4.8 Hz, CH), 7.23 (1H, d, J = 7.2 Hz, CH), 7.42 – 7.51 (3H, m, CH), 7.78 -7.92 (3H, m, CH), 8.44 (1H, s, CH), 9.16 (1H, s, CH).

4-Benzyl-nicotinic acid hydrazide (41)

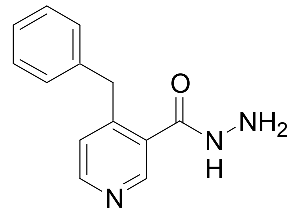

Hydrazine mono hydrate (0.44 ml, 8.8 mmol) and **36** (200 mg, 0.88 mmol) in 10 ml ethanol were refluxed for overnight. Solvent was then removed in *vacuo*, and the residue was partitioned between water and EtOAc. The organic phase was then separated and purified by column chromatography (MeOH : $CHCl_3$ = 1:10) to give 150 mg **41** as light yellow oil. Yield 75%. MS (ESI, positive ion) $[M+H]^+$ 228.1. ^1H NMR (400 MHz, $CDCl_3$) δ 4.18 (2H, s, benzyl CH_2), 7.13 (1H, d, J = 1.6 Hz, CH), 7.20 – 7.25 (3H, m, CH), 7.27 – 7.31 (2H, m, CH), 8.52 (1H, s, br, CH), 8.54 (1H, s, br, CH).

4-(4-*tert*-Butyl-benzyl)-nicotinic acid hydrazide (42)

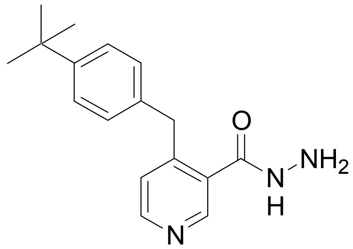

Hydrazine mono hydrate (0.86 ml, 17 mmol) and **37** (480 mg, 1.7 mmol) in 10 ml ethanol were refluxed for overnight. Solvent was then removed in *vacuo*, and the residue was partitioned between water and EtOAc. The organic phase was then separated and purified by column chromatography (MeOH : $CHCl_3$ = 1:10) to give 300 mg **42** as yellow solid. Yield 62%. MS (ESI, positive ion) $[M+H]^+$ 284.3. ^1H NMR (400 MHz, DMSO-d_6) δ 1.24 (9H, s, $C(CH_3)_3$), 4.06 (2H, s, benzyl CH_2), 4.54 (2H, s, br, NH_2), 7.15 (2H, d, J = 8.0 Hz, CH), 7.21 (1H, d, J = 5.6 Hz, CH), 7.29 (2H, d, J = 8.0 Hz, CH), 8.44 (1H, s, CH), 8.47 (1H, d, J = 5.6 Hz, CH), 9.72 (1H, s, br, NH).

4-(4-Trifluoromethyl-benzyl)-nicotinic acid hydrazide (43)

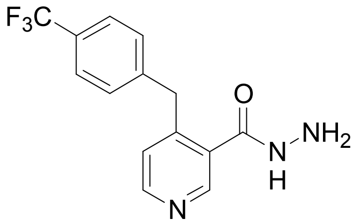

Hydrazine mono hydrate (0.81 ml, 16 mmol) and **38** (470 mg, 1.6 mmol) in 10 ml ethanol were refluxed for overnight. Solvent was then removed in *vacuo*, and the residue was diluted with water. The precipitate was collected and washed with $CHCl_3$ to give 270 mg **43** as light brown solid. Yield 57%. MS (ESI, positive ion) $[M+H]^+$ 296.1.^{1}H NMR (400 MHz, DMSO-d_6) δ 4.20 (2H, s, benzyl CH_2), 4.52 (2H, s, br, NH_2), 7.27 (1H, d, J = 5.2 Hz, CH), 7.46 (2H, d, J = 8.0 Hz, CH), 7.63 (2H, d, J = 8.0 Hz, CH), 8.47 (1H, s, CH), 8.51 (1H, d, J = 5.2 Hz, CH), 9.72 (1H, s, br, NH).

4-Naphthalen-2-ylmethyl-nicotinic acid hydrazide (44)

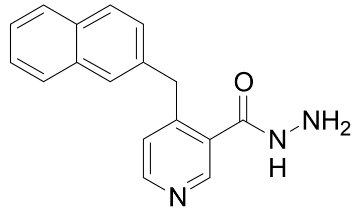

Hydrazine mono hydrate (0.36 ml, 7.2 mmol) and **39** (200 mg, 0.72 mmol) in 5 ml ethanol were refluxed for overnight. Solvent was then removed in *vacuo*, and the residue was partitioned between water and EtOAc. The organic phase was then separated and purified by column chromatography (MeOH : $CHCl_3$ = 1:10) to give 35 mg **44** as light yellow oil. Yield 18%. MS (ESI, positive ion) $[M+H]^+$ 278.2. ^{1}H NMR (400 MHz, $CDCl_3$) δ 4.35 (2H, s, benzyl CH_2), 7.17 (1H, d, J = 5.2 Hz, CH), 7.45 – 7.48 (2H, m, CH), 7.59 – 7.61 (1H, m, CH), 7.71 – 7.82 (4H, m, CH), 8.49 – 8.64 (2H, m, CH).

4-Naphthalen-1-ylmethyl-nicotinic acid hydrazide (45)

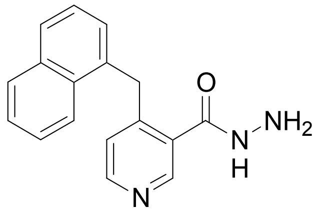

Hydrazine mono hydrate (0.36 ml, 7.2 mmol) and **40** (200 mg, 0.72 mmol) in 5 ml ethanol were refluxed for overnight. Solvent was then removed in *vacuo*, and the residue was partitioned between water and EtOAc. The organic phase was then separated and purified by column chromatography (MeOH : $CHCl_3$ = 1:10) to give 140 mg **45** as orange solid. Yield 70%. MS (ESI, positive ion) $[M+H]^+$ 278.2. ^1H NMR (400 MHz, DMSO-d_6) δ 4.59 (2H, s, benzyl CH_2), 6.86 (1H, d, *J* = 5.2 Hz, CH), 7.37 (1H, d, *J* = 6.8 Hz, CH), 7.46 – 7.52 (3H, m, CH), 7.86 (1H, d, *J* = 8.4 Hz, CH), 7.93 – 7.97 (2H, m, CH), 8.38 (1H, d, *J* = 5.2 Hz, CH), 8.52 (1H, s, CH), 9.84 (1H, s, NH).

4-[*N'*-(4-Benzyl-pyridine-3-carbonyl)-hydrazino]-4-oxo-but-2-enoic acid ethyl ester (25)

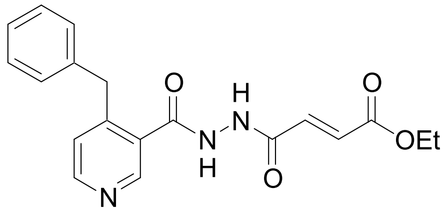

Ethyl pentafluorophenyl fumarate (230 mg, 0.73 mmol) and **41** (150 mg, 0.66 mmol) in Tetrahydrofuran : EtOAc (1:1, 8 ml) were stirred at room temperature for 24 h. Solvents were removed in *vacuo* and the residue was recrystallized from Hexane : EtOAc (5:1) to give 100 mg product as pale yellow solid. Yield 42%. m.p. 186 – 187^oC. MS (ESI, positive ion) $[M+H]^+$ 354.3. IR (KBr disk) υ/cm^{-1}: 3188 (NH), 3026 (NH), 1716 (CO ester), 1599 (CO amide), 1576 (CO amide). ^1H NMR (400 MHz, DMSO-d_6) δ 1.26 (3H, t, *J* = 7.2 Hz, OCH_2CH_3), 4.16 (2H, s, benzyl CH_2), 4.22 (2H, q, *J* = 7.2 Hz, OCH_2CH_3), 6.73 (1H, d, *J* = 15.6 Hz, CH), 7.10 (1H, d, *J* = 15.6 Hz, CH), 7.18 -7.24 (1H, m, CH), 7.26 – 7.30 (5H, m, CH), 8.54 (1H, d, *J* = 5.2 Hz, CH), 8.58 (1H, s, CH), 10.76 (1H, s, NH), 10.77 (1H, s, NH). ^{13}C NMR (100 MHz, DMSO-d_6) δ 13.9 (OCH_2CH_3), 36.9 (benzyl CH_2), 60.8 (OCH_2CH_3), 124.9 (CH), 126.3 (CH),

128.4 (C2'', C6''), 129.0 (C3'', C5''), 130.1 (C1'', C4''), 134.5 (C4'), 139.2 (C2'), 147.8 (C3'), 148.7 (C5'), 150.9 (C1'), 161.9 (CO), 164.6 (CO), 165.6 (CO).

4-{*N*'-[4-(4-*tert*-Butyl-benzyl)-pyridine-3-carbonyl]-hydrazino}-4-oxo-but-2-enoic acid ethyl ester (47)

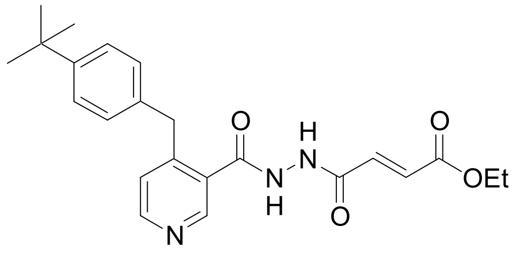

Ethyl pentafluorophenyl fumarate (290 mg, 0.93 mmol) and **42** (240 mg, 0.85 mmol) in Tetrahydrofuran : EtOAc (1:1, 10 ml) were stirred at room temperature for 24 h. Solvents were removed in *vacuo* and the residue was recrystallized from Hexane : EtOAc (5:1) to give 103 mg product as white solid. Yield 30%. m.p. 170 – 172°C. MS (ESI, positive ion) $[M+H]^+$ 410.2. IR (KBr disk) υ/cm^{-1}: 3183 (NH), 2966 (NH), 1717 (CO ester), 1603 (CO amide), 1578 (CO amide). ^{1}H NMR (400 MHz, DMSO-d$_6$) δ 1.24 (9H, s, $C(CH_3)_3$), 1.26 (3H, t, *J* = 7.2 Hz, OCH_2CH_3), 4.11 (2H, s, benzyl CH_2), 4.22 (2H, q, *J* = 7.2 Hz, OCH_2CH_3), 6.74 (1H, d, *J* = 15.6 Hz, CH), 7.10 (1H, d, *J* = 15.6 Hz, CH), 7.20 – 7.30 (5H, m, CH), 8.53 (1H, d, *J* = 5.2 Hz, CH), 8.57 (1H, s, CH), 10.77 (1H, s, NH), 10.78 (1H, s, NH). ^{13}C NMR (100 MHz, DMSO-d$_6$) δ 13.9 (OCH_2CH_3), 31.1 ($C(CH_3)_3$), 34.0 ($C(CH_3)_3$), 36.4 (benzyl CH_2), 60.8 (OCH_2CH_3), 124.9 (CH), 125.2 (C3'', C5''), 128.6 (C2'', C6''), 130.0 (CH), 130.1 (C1''), 134.5 (C4''), 136.1 (C4'), 147.8 (C2'), 148.6 (C3'), 148.8 (C5'), 150.9 (C1'), 161.9 (CO), 164.6 (CO), 165.6 (CO).

4-Oxo-4-{*N*'-[4-(4-trifluoromethyl-benzyl)-pyridine-3-carbonyl]-hydrazino}-but-2-enoic acid ethyl ester (48)

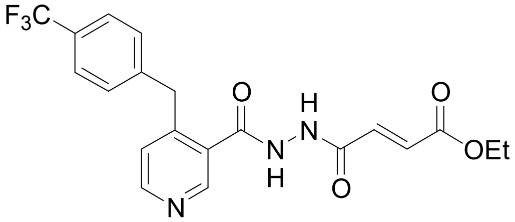

43 (210 mg, 0.71 mmol) and ethyl pentafluorophenyl fumarate (250 mg, 0.8 mmol) in Tetrahydrofuran : EtOAc (1:1, 10 ml) were stirred at room temperature for 24 h. Solvents were removed in *vacuo* and the residue was recrystallized from Hexane : EtOAc (5:1) to give 160 mg product as light brown crystals. Yield 54%. m.p. 221 – 223°C. MS (ESI, positive ion) $[M+H]^+$ 422.0. IR (KBr disk) υ/cm^{-1}: 3144 (NH), 2989 (NH), 1722 (CO ester), 1616 (CO amide). ^{1}H NMR (400 MHz, DMSO-d_6) δ 1.26 (3H, t, J = 7.2 Hz, OCH_2CH_3), 4.20 (2H, s, benzyl CH_2), 4.24 (2H, q, J = 7.2 Hz, OCH_2CH_3), 6.74 (1H, d, J = 15.6 Hz, CH), 7.10 (1H, d, J = 15.6 Hz, CH), 7.34 (1H, d, J = 5.2 Hz, CH), 7.52 (2H, d, J = 8.0 Hz, CH), 7.65 (2H, d, J = 8.0 Hz, CH), 8.58 (1H, d, J = 5.2 Hz, CH), 8.62 (1H, s, CH), 10.78 (2H, br, NH). ^{13}C NMR (100 MHz, DMSO-d_6) δ 13.9 (OCH_2CH_3), 36.6 (benzyl CH_2), 60.8 (OCH_2CH_3), 125.1 (CH), 125.2 (CH), 125.3 (C1’’), 129.7 (C2’’, C6’’, C3’’, C5’’), 130.0 (C4’’), 130.1 (C4’), 134.4 (C2’), 144.1 (CF_3), 147.6 (C3’), 147.9 (C5’), 151.1 (C1’), 161.9 (CO), 164.6 (CO), 165.5 (CO).

4-[*N*'-(4-Naphthalen-2-ylmethyl-pyridine-3-carbonyl)-hydrazino]-4-oxo-but-2-enoic acid ethyl ester (49)

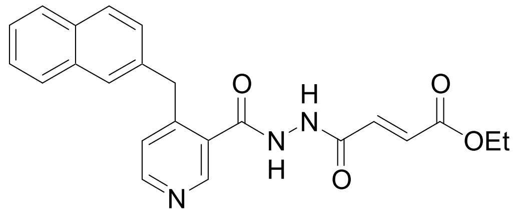

Ethyl pentafluorophenyl fumarate (40 mg, 0.14 mmol) and **44** (30 mg, 0.11 mmol) in Tetrahydrofuran : EtOAc (1:1, 2 ml) were stirred at room temperature for 24 h. Solvents were removed in *vacuo* and the residue was recrystallized from Hexane : EtOAc (5:1) to give 40 mg product as orange solid. Yield 91%. m.p. 154 – 156°C. MS (ESI, positive ion) $[M+H]^+$ 404.2. IR (KBr disk) υ/cm^{-1}: 3181 (NH), 3022 (NH), 1714 (CO ester), 1599 (CO amide), 1578 (CO amide). ^{1}H NMR (400 MHz, DMSO-d_6) δ

1.26 (3H, t, J = 7.2 Hz, OCH_2CH_3), 4.22 (2H, q, J = 7.2 Hz, OCH_2CH_3), 4.34 (2H, s, benzyl CH_2), 6.75 (1H, d, J = 15.6 Hz, CH), 7.12 (1H, d, J = 15.6 Hz, CH), 7.33 (1H, d, J = 4.8 Hz, CH), 7.44 – 7.49 (3H, m, CH), 7.80 – 7.87 (4H, m, CH), 8.54 (1H, d, J = 4.8 Hz, CH), 8.62 (1H, s, CH), 10.80 (1H, s, NH), 10.83 (1H, s, NH). ^{13}C NMR (100 MHz, DMSO-d_6) δ 13.9 (OCH_2CH_3), 37.0 (benzyl CH_2), 60.8 (OCH_2CH_3), 125.0 (CH), 125.5 (CH), 126.0 (C3''), 127.2 (C2''), 127.3 (C1''), 127.4 (C4''), 127.4 (C5''), 127.5 (C10''), 128.0 (C6''), 130.1 (C9''), 131.6 (C7''), 133.0 (C8''), 134.5 (C4'), 136.8 (C2'), 147.9 (C3'), 148.5 (C5'), 151.0 (C1'), 161.9 (CO), 164.6 (CO), 165.6 (CO).

4-[*N'*-(4-Naphthalen-1-ylmethyl-pyridine-3-carbonyl)-hydrazino]-4-oxo-but-2-enoic acid ethyl ester (50)

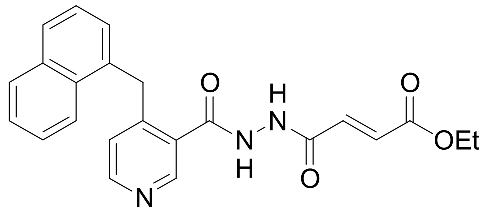

Ethyl pentafluorophenyl fumarate (176 mg, 0.56 mmol) and **45** (120 mg, 0.44 mmol) in Tetrahydrofuran : EtOAc (1:1, 8 ml) were stirred at room temperature for 24 h. Solvents were removed in *vacuo* and the residue was recrystallized from Hexane : EtOAc (5:1) to give 140 mg product as yellow solid. Yield 79%. m.p. 151 – 153°C. MS (ESI, positive ion) $[M+H]^+$ 403.9. IR (KBr disk) υ/cm^{-1}: 3177 (NH), 3024 (NH), 1714 (CO ester), 1601 (CO amide), 1577 (CO amide). ^{1}H NMR (400 MHz, DMSO-d_6) δ 1.25 (3H, t, J = 7.2 Hz, OCH_2CH_3), 4.21 (2H, q, J = 7.2 Hz, OCH_2CH_3), 4.63 (2H, s, benzyl CH_2), 6.73 (1H, d, J = 15.6 Hz, CH), 6.88 (1H, d, J = 4.8 Hz, CH), 7.10 (1H, d, J = 15.6 Hz, CH), 7.40 (1H, d, J = 6.8 Hz, CH), 7.46 – 7.53 (3H, m, CH), 7.87 (1H, d, J = 8.0 Hz, CH), 7.94 – 7.97 (2H, m, CH), 8.44 (1H, d, J = 4.8 Hz, CH), 8.66 (1H, s, CH), 10.83 (1H, s, NH), 10.90 (1H, s, NH). ^{13}C NMR (100 MHz, DMSO-d_6) δ 13.9 (OCH_2CH_3), 34.2 (benzyl CH_2), 60.8 (OCH_2CH_3), 123.9 (CH), 124.0 (CH), 125.6 (C2''), 125.7 (C3''), 126.3 (C10''), 127.4 (C5''), 127.9 (C4''), 128.5 (C1''), 130.0 (C9''), 130.1 (C6''), 131.3 (C8''), 133.5 (C7''), 134.4 (C4'), 134.5 (C2'), 147.7 (C3'), 148.3 (C5'), 151.0 (C1'), 161.9 (CO), 164.6 (CO), 165.7 (CO).

5-Benzyl-nicotinic acid methyl ester (52)

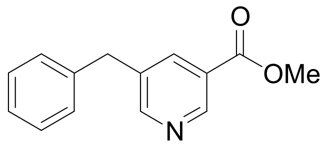

To a solution of **51** (2.5 g, 11.6 mmol) in 15 ml DMF was added subsequently LiCl (0.98 g, 23.2 mmol), $Pd(PPh_3)_4$ (0.7 g, 0.58 mmol), and the benzyl indium bromide (30 mmol) DMF solution (15 ml, prepared by treating benzyl bromide with indium dust in DMF at room temperature). The reaction mixture was heated to 100°C and stirred for overnight. The reaction mixture was then diluted with Et_2O (100 ml) and washed with water (3×20 ml). The organic layer was separated and the solvent was removed in *vacuo*. The residue was purified by column chromatography (Hexane : EtOAc = 5 : 1) to give 1 g **8** as yellow oil. Yield 38%. MS (ESI, positive ion) $[M+H]^+$ 228.0. ^1H NMR (400 MHz, $CDCl_3$) δ 3.91 (3H, s, OCH_3), 4.02 (2H, s, benzyl CH_2), 7.16 – 7.36 (5H, m, CH), 8.09 (1H, t, J = 1.6 Hz, CH), 8.64 (1H, d, J = 1.6 Hz, CH), 9.06 (1H, d, J = 1.6 Hz, CH).

5-Benzyl-nicotinic acid hydrazide (53)

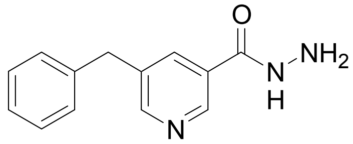

Hydrazine mono hydrate (1 ml, 22 mmol) and **52** (500 mg, 2.2 mmol) in 10 ml ethanol were refluxed for overnight. Solvent was then removed in *vacuo*, and the residue was diluted with water. The precipitate coming out was collected to give 400 mg **53** as gray crystals. Yield 80%. MS (ESI, positive ion) $[M+H]^+$ 228.1. ^1H NMR (400 MHz, DMSO-d_6) δ 4.02 (2H, s, benzyl CH_2), 4.53 (2H, s, br, NH_2), 7.19 – 7.32 (5H, m, CH), 7.99 (1H, t, J = 2 Hz, CH), 8.62 (1H, d, J = 2.0 Hz, CH), 8.79 (1H, d, J = 2.0Hz, CH), 9.93 (1H, s, br, NH).

4-[*N*'-(5-Benzyl-pyridine-3-carbonyl)-hydrazino]-4-oxo-but-2-enoic acid ethyl ester (54)

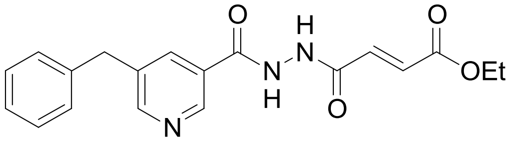

Ethyl pentafluorophenyl fumarate (343 mg, 1.1 mmol) and **53** (227 mg, 1 mmol) in Tetrahydrofuran : EtOAc (1:1, 10 ml) were stirred at room temperature for 24 h. Solvents were removed in *vacuo* and the residue was recrystallized from Hexane : EtOAc (5:1) to give 180 mg product as gray solid. Yield 51%. m.p. 188 – 189°C. MS (ESI, positive ion) $[M+H]^+$ 354.1. IR (KBr disk) υ/cm^{-1}: 3170 (NH), 3031 (NH), 1708 (CO ester), 1602 (CO amide), 1584 (CO amide). ^1H NMR (400 MHz, DMSO-d_6) δ 1.26 (3H, t, J = 7.2 Hz, OCH_2CH_3), 4.06 (2H, s, benzyl CH_2), 4.21 (2H, q, J = 7.2 Hz, OCH_2CH_3), 6.70 (1H, d, J = 15.6 Hz, CH), 7.09 (1H, d, J = 15.6 Hz, CH), 7.22 (1H, m, CH), 7.28 – 7.34 (4H, m, CH), 8.05 (1H, d, J = 2.0Hz, CH), 8.70 (1H, d, J = 2.0 Hz, CH), 8.86 (1H, d, J = 2.0 Hz, CH), 10.70 (1H, s, NH), 10.86 (1H, s, NH). ^{13}C NMR (100 MHz, DMSO-d_6) δ 13.9 (OCH_2CH_3), 37.7 (benzyl CH_2), 60.8 (OCH_2CH_3), 126.3 (CH), 127.7 (CH), 128.6 (C2'', C6''), 128.7 (C3'', C5''), 130.0 (C4''), 134.6 (C1''), 135.1 (C4'), 136.8 (C2'), 139.9 (C3'), 146.1 (C5'), 152.6 (C1'), 161.9 (CO), 163.8 (CO), 164.6 (CO).

4-(4'-Methoxy-biphenyl-4-ylmethyl)-nicotinic acid methyl ester (56)

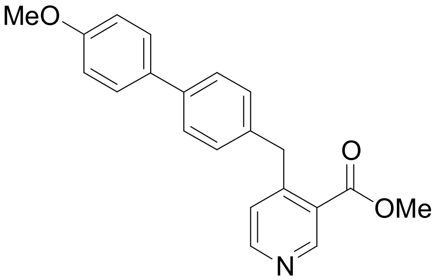

To a solution of **55** (0.3 g, 0.85 mmol) in 5 ml DMF was added 4-methoxyphenylboronic acid (0.155 g, 1.02 mmol), potassium carbonate (0.29 g, 2.1 mmol), and $Pd(PPh_3)_4$ (21 mg, 0.017 mmol). The reaction mixture was heated to 100°C and stirred for overnight. Upon cooling, the reaction mixture was poured into water (50 ml) and extracted with EtOAc (3×20 ml). EtOAc was then removed in *vacuo*, and the residue was purified by column chromatography (Hexane : EtOAc = 5 : 1) to give 100 mg **56** as yellow crystals. Yield 35%. MS (ESI, positive ion) $[M+H]^+$

334.1. ^{1}H NMR (400 MHz, $CDCl_3$) δ 3.85 (3H, s, OCH_3), 3.92 (3H, s, OCH_3), 4.42 (2H, s, benzyl CH_2), 6.97 (2H, d, J = 8.8 Hz, CH), 7.14 (1H, d, J = 5.2 Hz, CH), 7.20 (2H, d, J = 8.4 Hz, CH), 7.48 – 7.52 (4H, m, CH), 8.60 (1H, d, J = 5.2 Hz, CH), 9.10 (1H, s, CH).

4-(4'-Methoxy-biphenyl-4-ylmethyl)-nicotinic acid hydrazide (57)

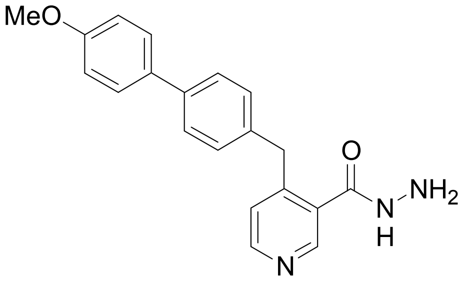

Hydrazine mono hydrate (0.15 ml, 3 mmol) and **56** (100 mg, 0.3 mmol) in 5 ml ethanol were refluxed for overnight. Upon cooling, the precipitate was collected to give 83 mg **57** as white crystals. Yield 83%. MS (ESI, positive ion) $[M+H]^+$ 334.1. ^{1}H NMR (400 MHz, $CDCl_3$) δ 3.85 (3H, s, OCH_3), 4.13 (2H, s, br, NH_2), 4.22 (2H, s, benzyl CH_2), 6.96 (2H, d, J = 8.8 Hz, CH), 7.15 (1H, d, J = 5.2 Hz, CH), 7.15 (2H, d, J = 8.4 Hz, CH), 7.47 – 7.51 (4H, m, CH), 8.53 (1H, d, J = 5.2 Hz, CH), 8.61 (1H, s, CH), 9.01 (1H, s, NH).

4-{*N'*-[4-(4'-Methoxy-biphenyl-4-ylmethyl)-pyridine-3-carbonyl]-hydrazino}-4-oxo-but-2-enoic acid ethyl ester (58)

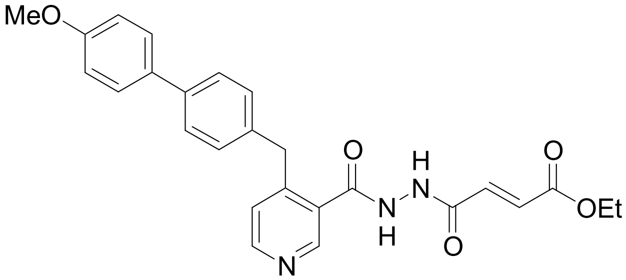

Ethyl pentafluorophenyl fumarate (104 mg, 0.33 mmol) and **57** (83 mg, 0.25 mmol) in Tetrahydrofuran : EtOAc (1:1, 6 ml) were stirred at room temperature for 24 h. Solvents were removed in *vacuo* and the residue was recrystallized from Hexane : EtOAc (5:1) to give 66mg product as white solid. Yield 58%. m.p. 190 – 192°C. MS (ESI, positive ion) $[M+H]^+$ 354.1. IR (KBr disk) υ/cm^{-1}: 3204 (NH), 3024 (NH), 1715 (CO ester), 1603 (CO amide), 1578 (CO amide). ^{1}H NMR (400 MHz, DMSO-d_6) δ 1.26 (3H, t, J = 7.2 Hz, OCH_2CH_3), 3.78 (3H, s, OCH_3), 4.19 (2H, s, benzyl CH_2),

4.23 (2H, q, J = 7.2 Hz, OCH_2CH$_3$), 6.74 (1H, d, J = 15.6 Hz, CH), 7.00 (2H, d, J = 8.8 Hz, CH), 7.11 (1H, d, J = 15.6 Hz, CH), 7.32 (1H, d, J = 5.2 Hz, CH), 7.35 (2H, d, J = 8.0 Hz, CH), 7.52 (2H, d, J = 8.0 Hz, CH), 7.56 (2H, d, J = 8.8 Hz, CH), 8.56 (1H, d, J = 5.2 Hz, CH), 8.60 (1H, s, CH), 10.79 (2H, s, NH). ^{13}C NMR (100 MHz, DMSO-d$_6$) δ13.9 (OCH$_2$$CH_3$), 36.5 (benzyl CH$_2$), 55.1 (OCH$_3$), 60.8 (O$CH_2CH_3$), 114.3 (C3''', C5'''), 125.0 (CH), 126.2 (C2''', C6'''), 127.5 (C2'', C6''), 129.5 (C3'', C5''), 130.0 (CH), 130.1 (C1'''), 132.2 (C4''), 134.5 (C1''), 137.7 (C4'), 137.9 (C2'), 147.8 (C3'), 148.7 (C5'), 150.9 (C1'), 158.7 (C4'''), 161.9 (CO), 164.6 (CO), 165.6 (CO).

4-Benzyl-4*H*-pyridine-1,2-dicarboxylic acid 2-methyl ester 1-phenyl ester (60)

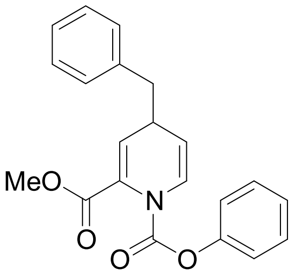

Phenyl chloroformate (0.82 ml, 6.5 mmol) was added dropwise to a solution of **59** (0.79 ml, 6.5 mmol) in 15 ml dry Tetrahydrofuran at 0ºC. The reaction mixture was stirred for 1 h in an ice bath. Benzyl zinc bromide (5 mmol) in 15 ml dry Tetrahydrofuran (prepared by treating benzyl bromide with zinc dust in dry Tetrahydrofuran at room temperature) was then added dropwise to the cold phenoxycarbonyl pyridinium chloride solution. The reaction mixture was stirred for 2 h in an ice bath. The reaction was then quenched by 20 ml saturated NH_4Cl solution. The reaction mixture was then extracted with EtOAc (3 × 20 ml), and the extract was washed with saturated $NaHCO_3$, saturated NH_4Cl, and brine. The extract was concentrated in *vacuo*, and the residue was purified by column chromatography (Hexane : EtOAc = 4 : 1) to give 500 mg **60** as yellow oil. Yield 29%. MS (ESI, positive ion) $[M+H]^+$ 350.0. ^{1}H NMR (400 MHz, CDCl$_3$) δ 2.80 – 2.82 (2H, dd, J_1 = 7.2 Hz, J_2 = 3.2Hz, benzyl CH$_2$), 3.29 – 3.35 (1H, m, CH), 3.76 (3H, s, OCH$_3$), 5.06 – 5.10 (1H, m, CH), 5.33 (1H, s, br, CH), 5.92 – 5.94 (1H, dd, J_1 = 4.8 Hz, J_2 = 1.6 Hz, CH), 7.11 – 7.38 (10H, m, CH).

4-Benzyl-pyridine-2-carboxylic acid methyl ester (61)

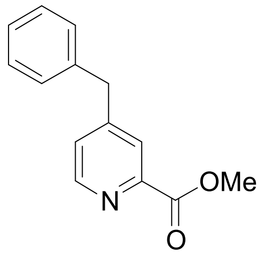

A solution of **60** (500 mg, 1.44 mmol) and sulphur (46 mg, 1.44 mmol) in 3 ml decalin was heated to reflux for 5 h. The reaction mixture was cooled down to the room temperature and extracted by 1 N HCl (4 × 4 ml). The aqueous extract was basified with 2 N NaOH to pH = 8 and extracted by DCM (3 × 10 ml). The DCM was removed in *vacuo*, and the residue was purified by column chromatography (Hexane : EtOAc = 3 : 1) to give 130 mg **61** as brown oil. Yield 40%. MS (ESI, positive ion) $[M+H]^+$ 228.0. ^1H NMR (400 MHz, $CDCl_3$) δ 3.98 (3H, s, OCH_3), 4.03 (2H, s, benzyl CH_2), 7.16 – 7.18 (2H, m, CH), 7.23 – 7.34 (4H, m, CH), 7.99 (1H, s, CH), 8.62 (1H, d, J = 4.8 Hz, CH).

4-Benzyl-pyridine-2-carboxylic acid hydrazide (62)

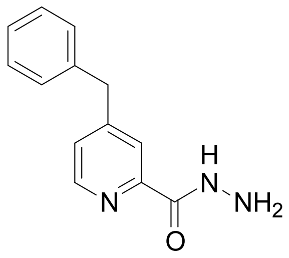

Hydrazine mono hydrate (0.3 ml, 5.8 mmol) and **61** (130 mg, 0.58 mmol) in 5 ml ethanol were refluxed for overnight. Solvent was then removed in *vacuo*, and the residue was partitioned between water and EtOAc. The organic phase was then separated, washed with brine and dried over Na_2SO_4. EtOAc was removed in *vacuo* to give 110 mg **62** as brown oil. Yield 85%. MS (ESI, positive ion) $[M+H]^+$ 228.2. ^1H NMR (400 MHz, $CDCl_3$) δ 4.03 (2H, s, benzyl CH_2), 7.17 – 7.33 (6H, m, CH), 8.02 (1H, s, CH), 8.41 (1H, d, J = 4.8 Hz, CH), 8.97 (1H, s, br, NH).

4-[*N'*-(4-Benzyl-pyridine-2-carbonyl)-hydrazino]-4-oxo-but-2-enoic acid ethyl ester (63)

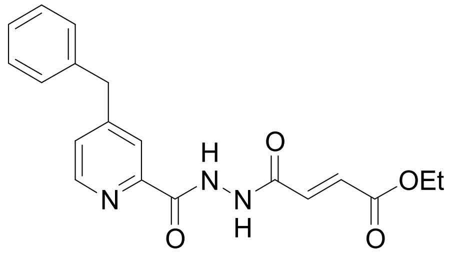

Ethyl pentafluorophenyl fumarate (172 mg, 0.55 mmol) and **62** (110 mg**,** 0.5 mmol**)** in Tetrahydrofuran : EtOAc (1:1, 6 ml) were stirred at room temperature for 24 h. Solvents were removed in *vacuo* and the residue was recrystallized from Hexane : EtOAc (5:1) to give 58 mg product as yellow solid. Yield 33%. m.p. 191 – 193°C. MS (ESI, positive ion) $[M+H]^+$ 354.4. IR (KBr disk) υ/cm^{-1}: 3336 (NH), 3192 (NH), 1716 (CO ester), 1619 (CO amide), 1598 (CO amide). ^1H NMR (400 MHz, DMSO-d_6) δ 1.25 (3H, t, J = 7.2 Hz, OCH_2CH_3), 4.10 (2H, s, benzyl CH_2), 4.21 (2H, q, J = 7.2 Hz, OCH_2CH_3), 6.68 (1H, d, J = 15.6 Hz, CH), 7.09 (1H, d, J = 15.6 Hz, CH), 7.21 -7.35 (5H, m, CH), 7.52 -7.53 (1H, m, CH), 7.89 (1H, s, CH), 8.57 (1H, d, J = 5.2 Hz, CH), 10.69 (1H, s, NH), 10.76 (1H, s, NH). ^{13}C NMR (100 MHz, DMSO-d_6) δ 13.9 (OCH_2CH_3), 40.0 (benzyl CH_2), 60.8 (OCH_2CH_3), 122.4 (CH), 126.4 (CH), 127.1 (C4''), 128.6 (C2'', C6''), 128.9 (C3'', C5''), 129.8 (C1''), 134.7 (C4'), 139.1 (C2'), 148.7 (C3'), 148.8 (C5'), 152.2 (C1'), 161.3 (CO), 162.2 (CO), 164.6 (CO).

The NMR spectra for selected compounds

^{1}H NMR spectrum of 12

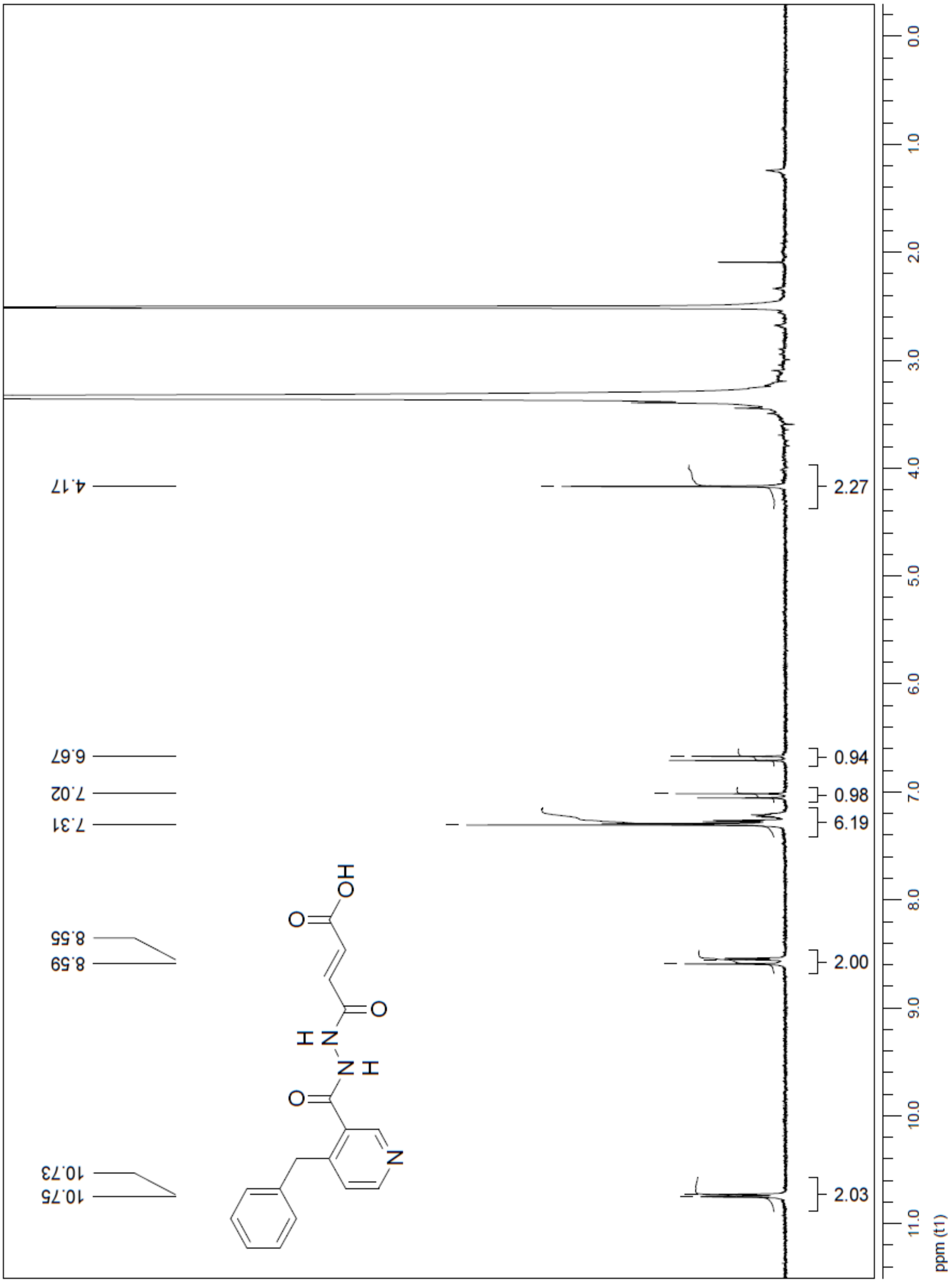

^{1}H NMR spectrum of 13

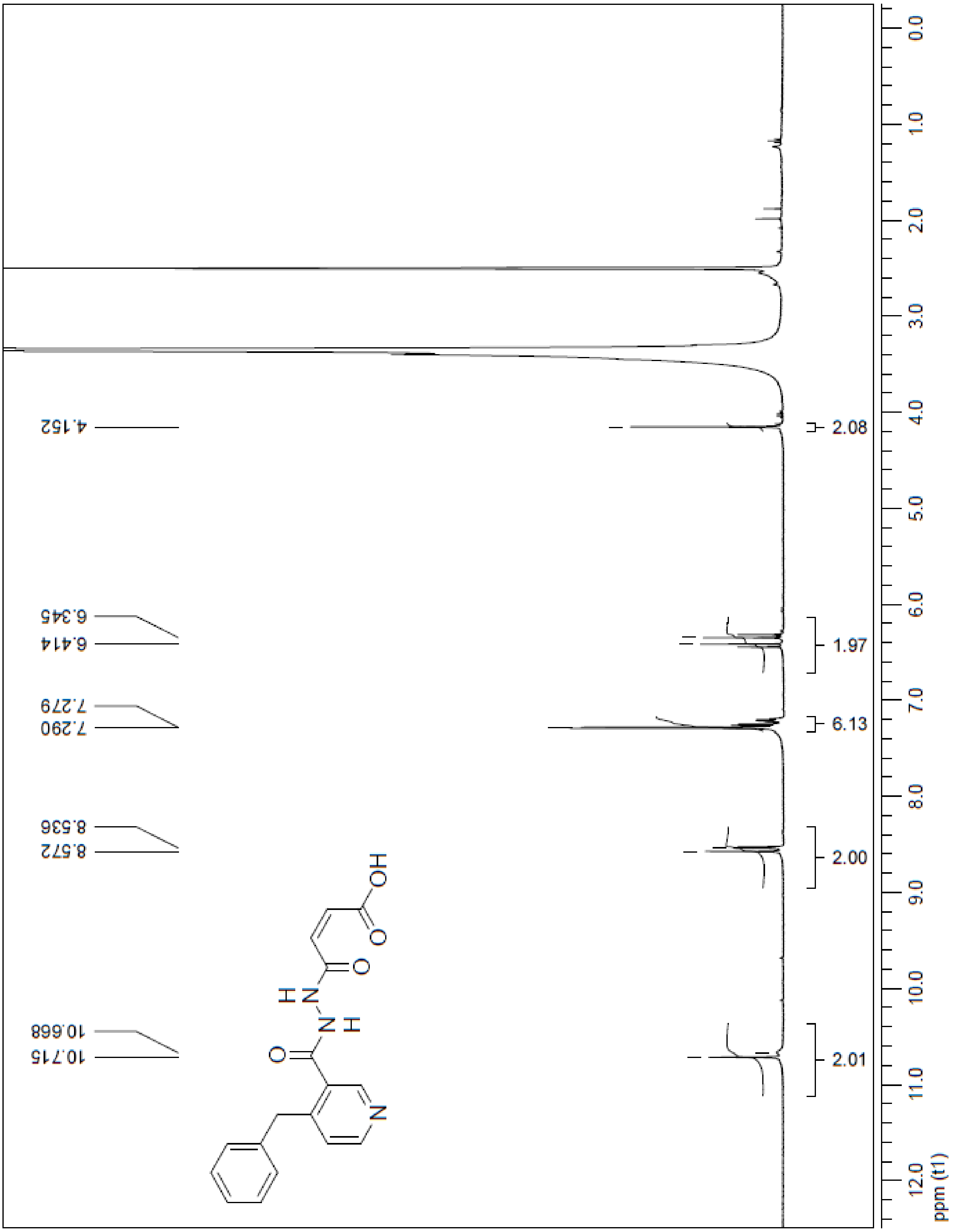

^{1}H NMR spectrum of 14

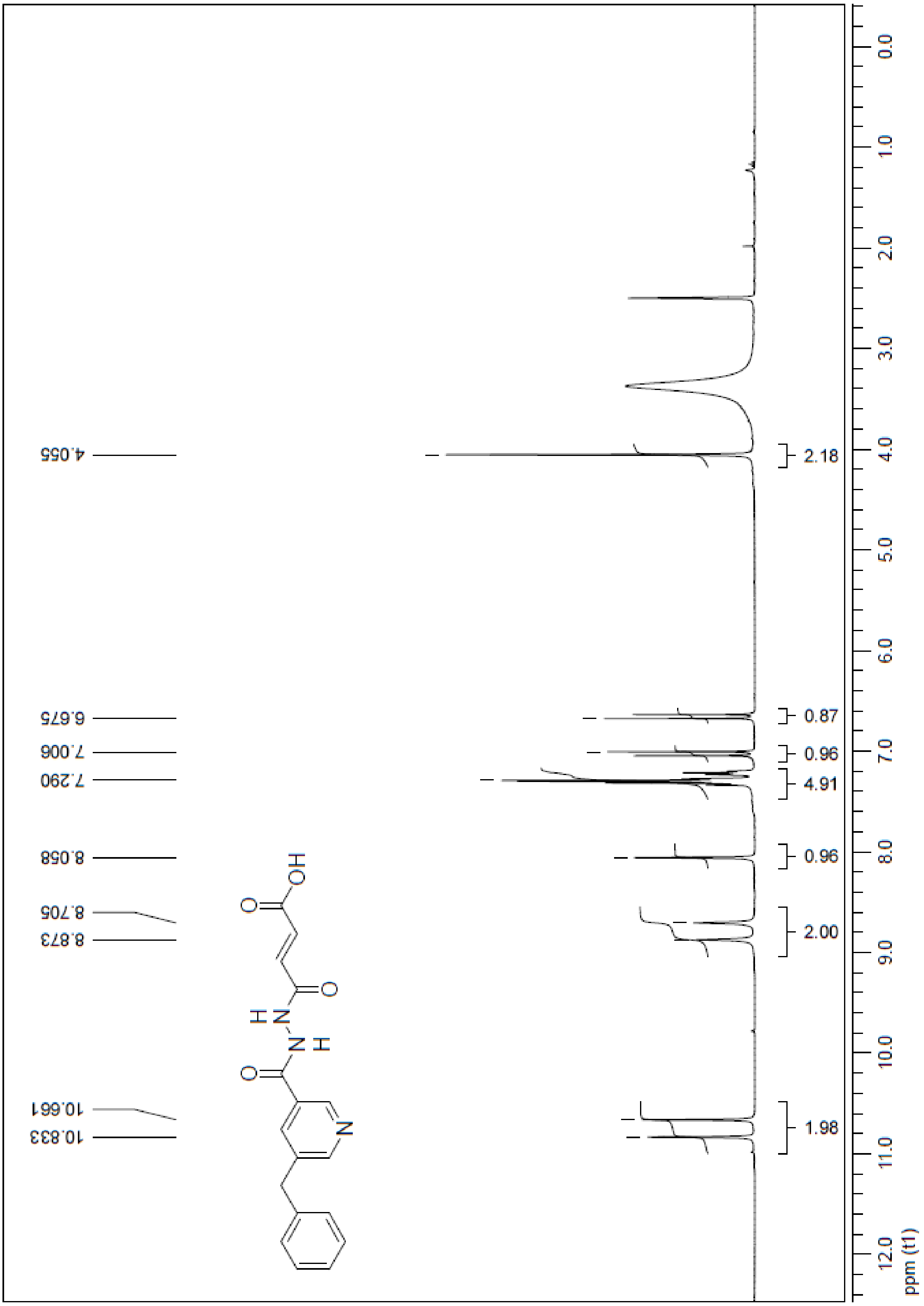

^{1}H NMR spectrum of 15

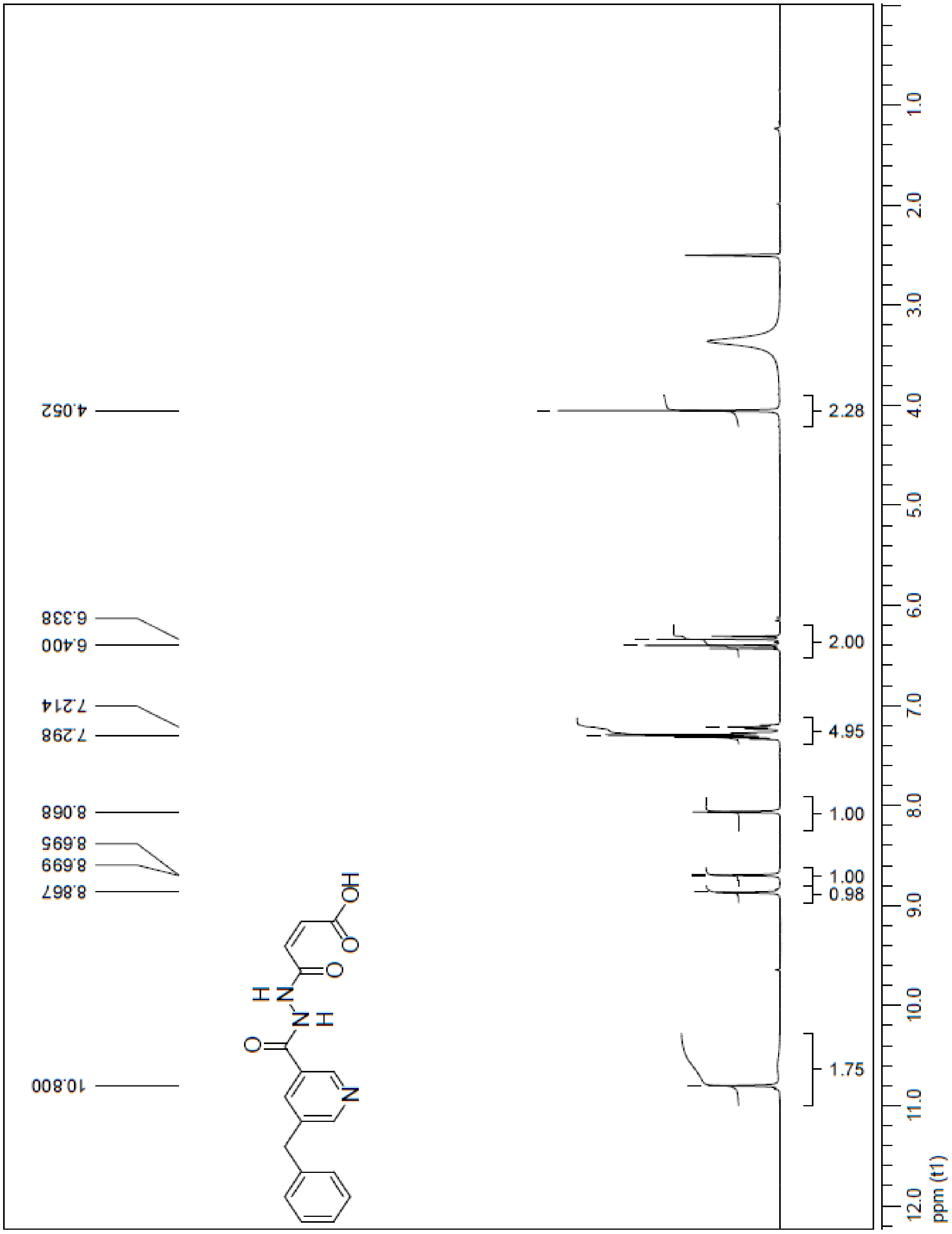

^{1}H NMR spectrum of 16

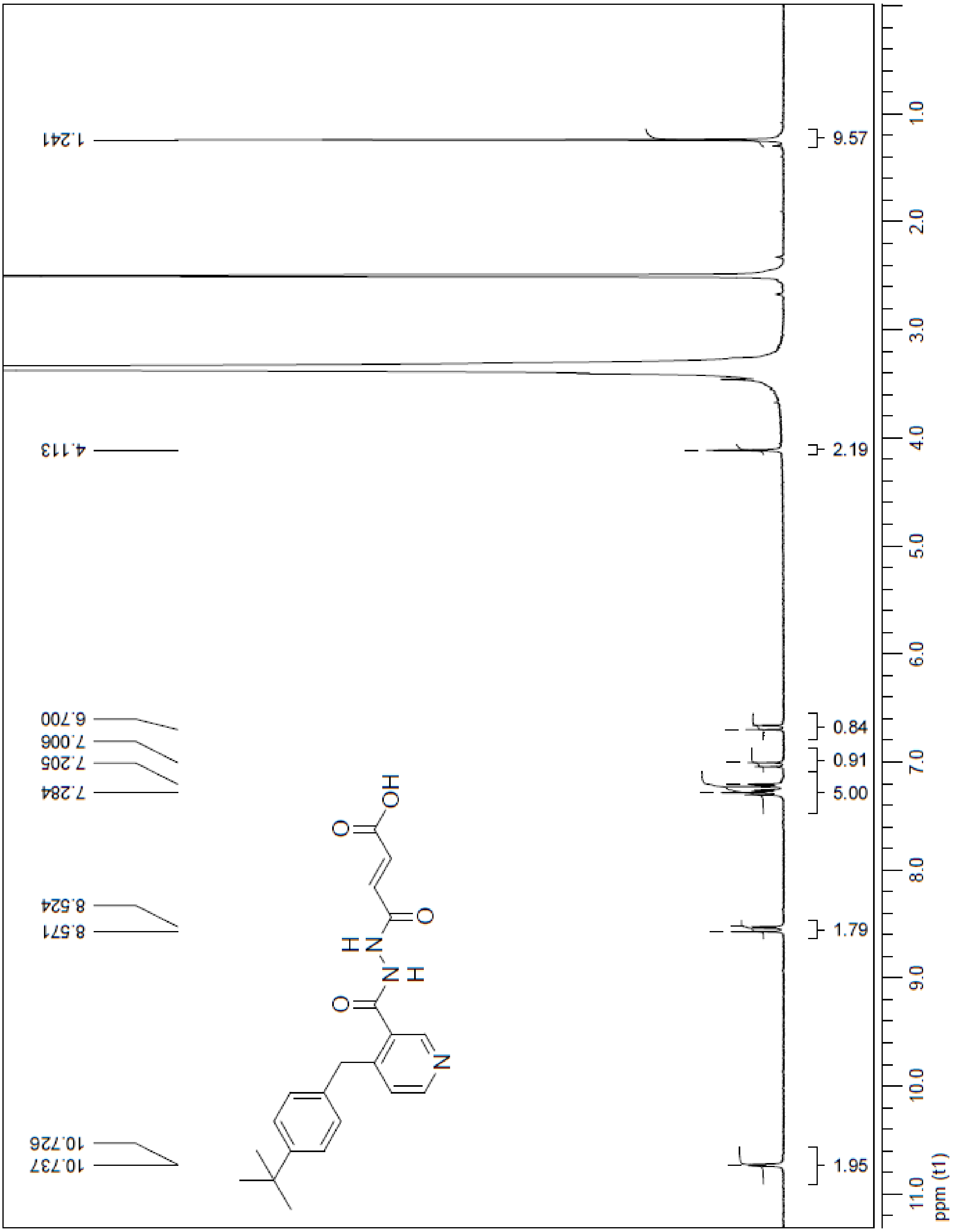

^{1}H NMR spectrum of 17

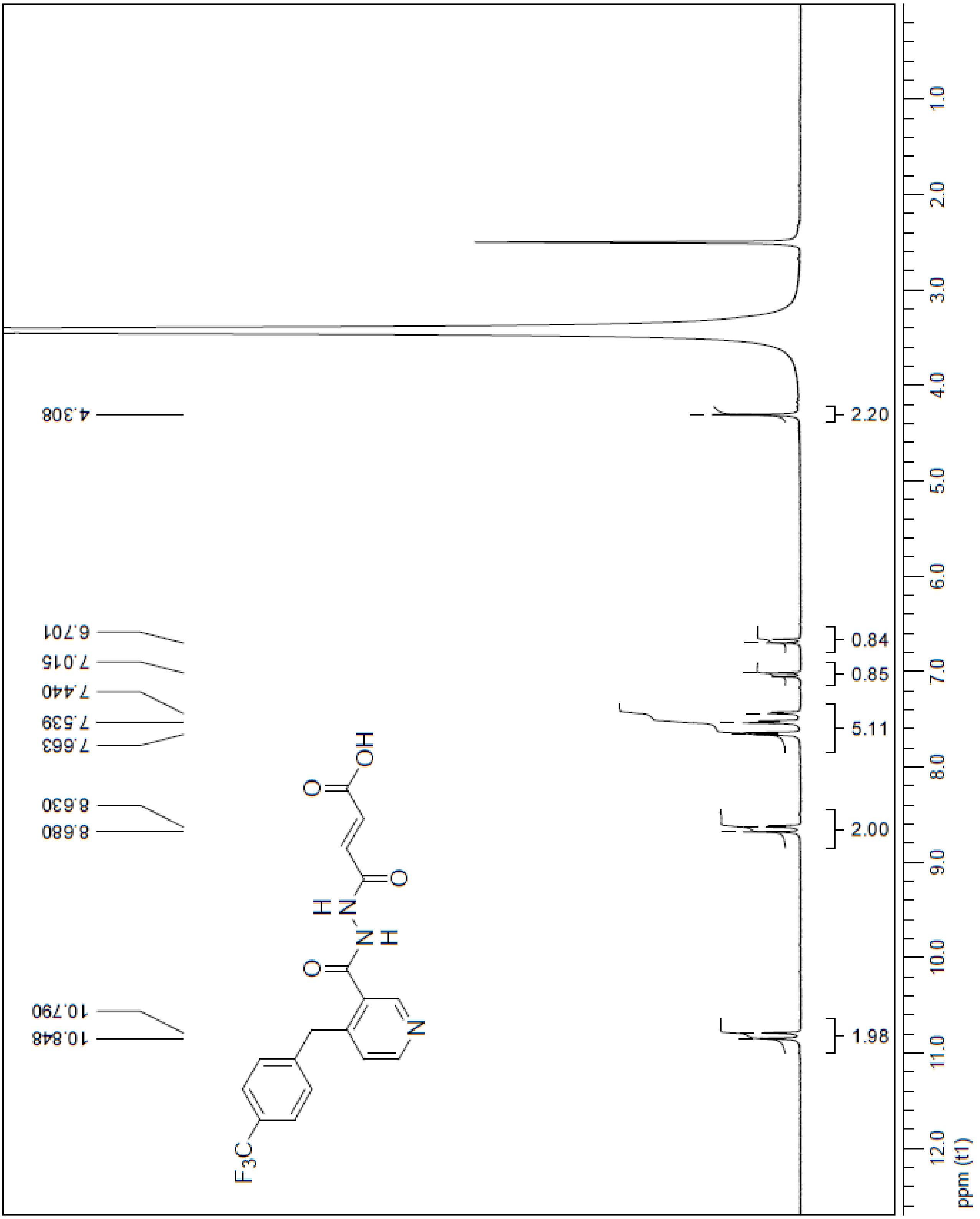

^{1}H NMR spectrum of 18

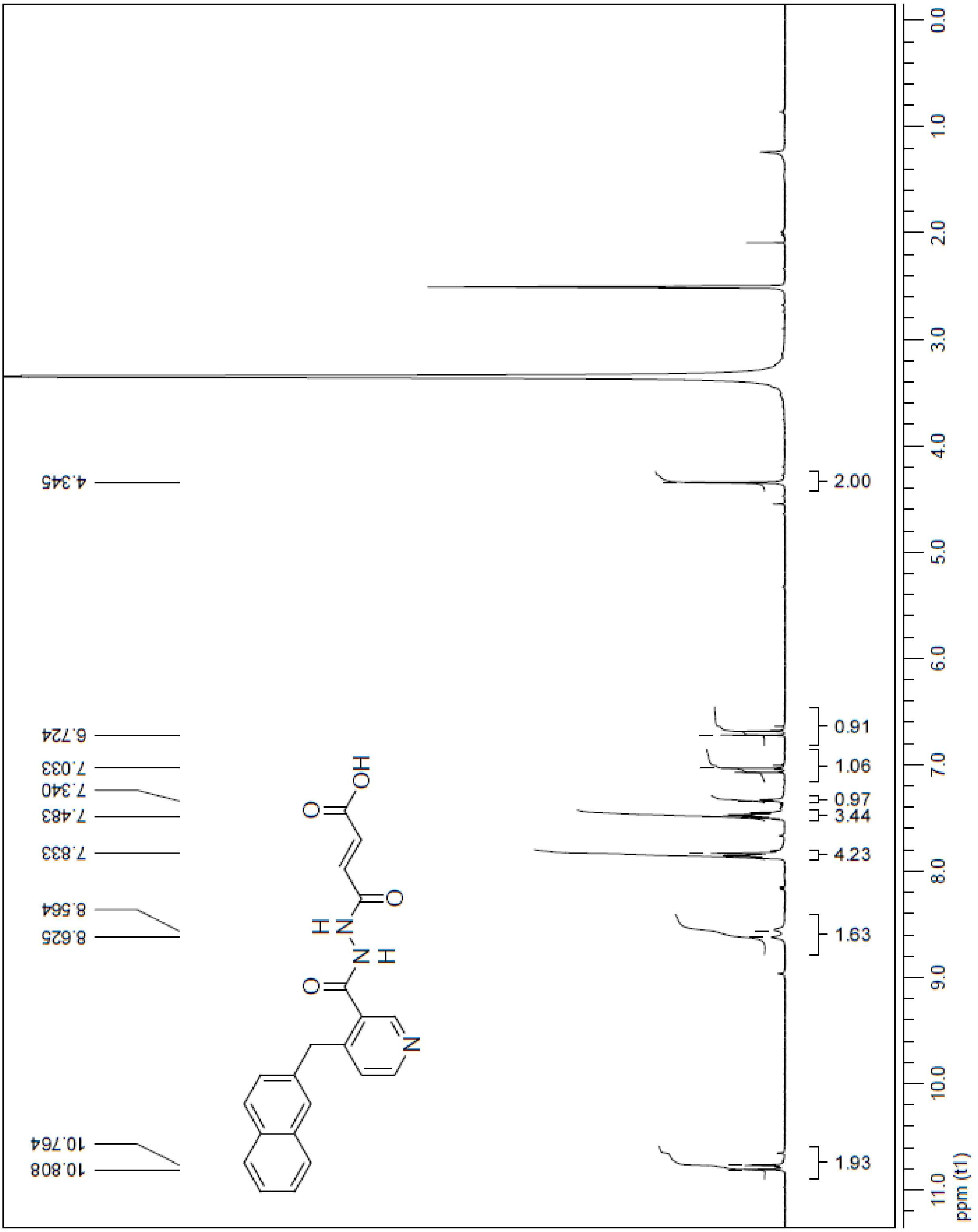

^{1}H NMR spectrum of 19

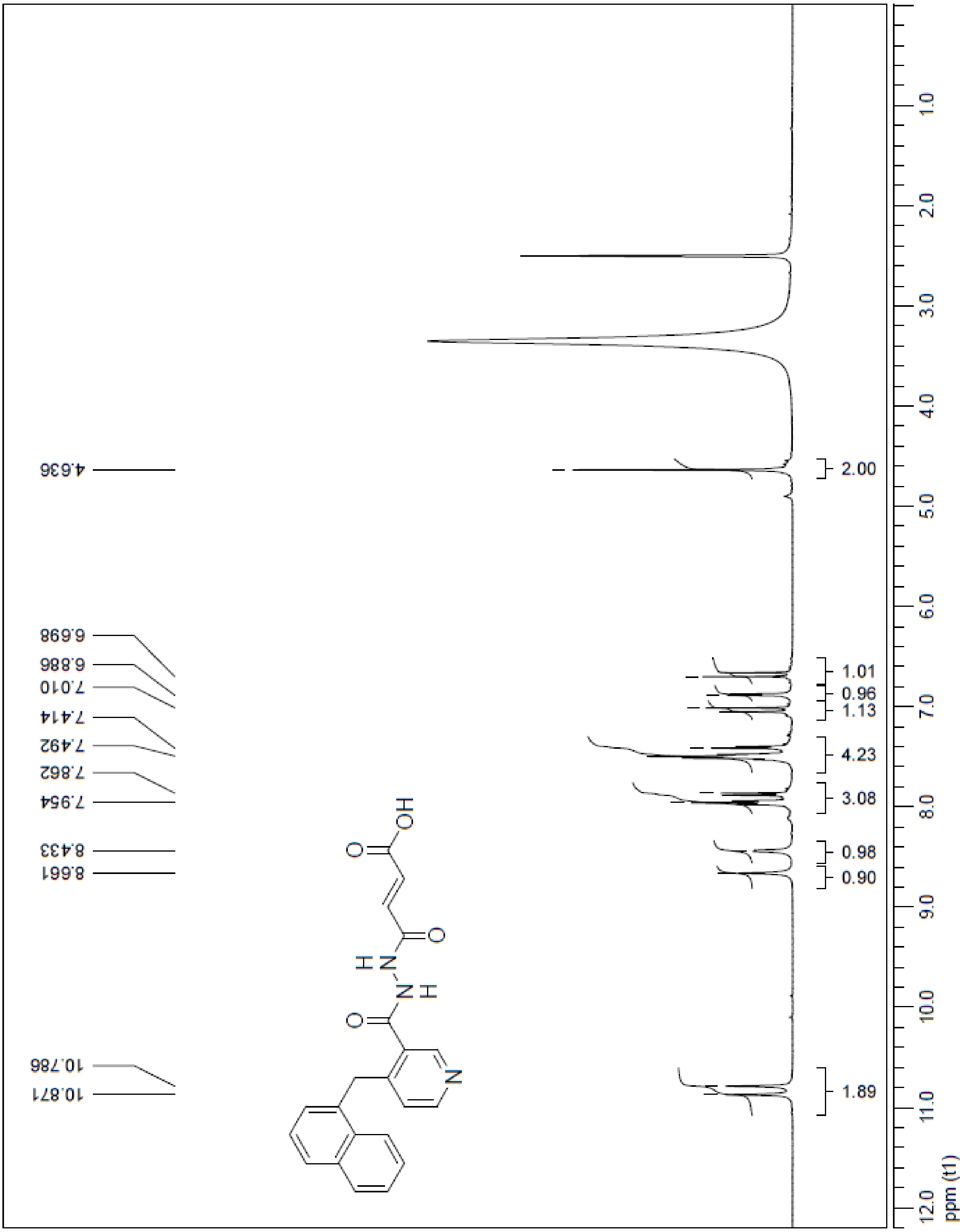

^{1}H NMR spectrum of 20

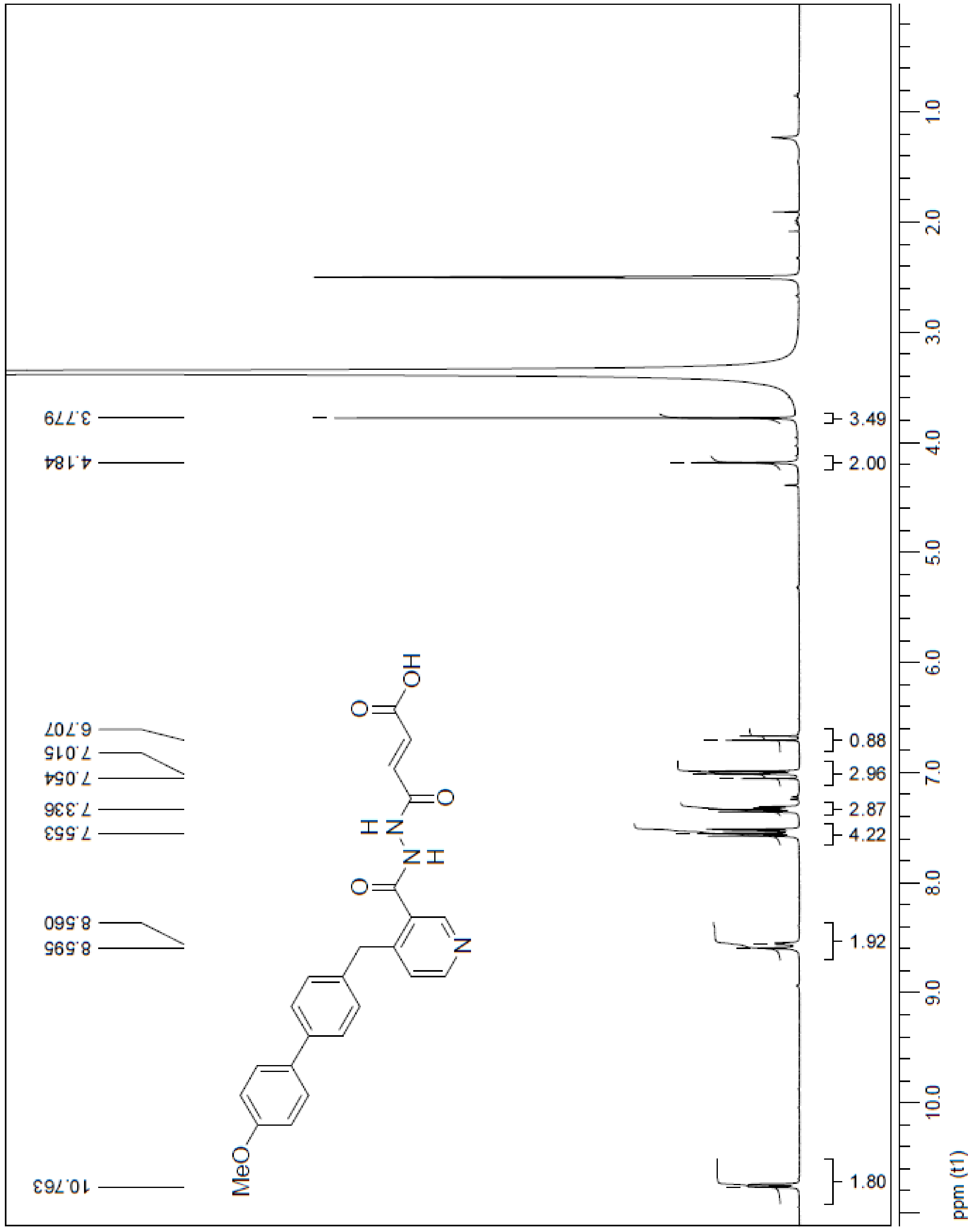

^{1}H NMR spectrum of 22

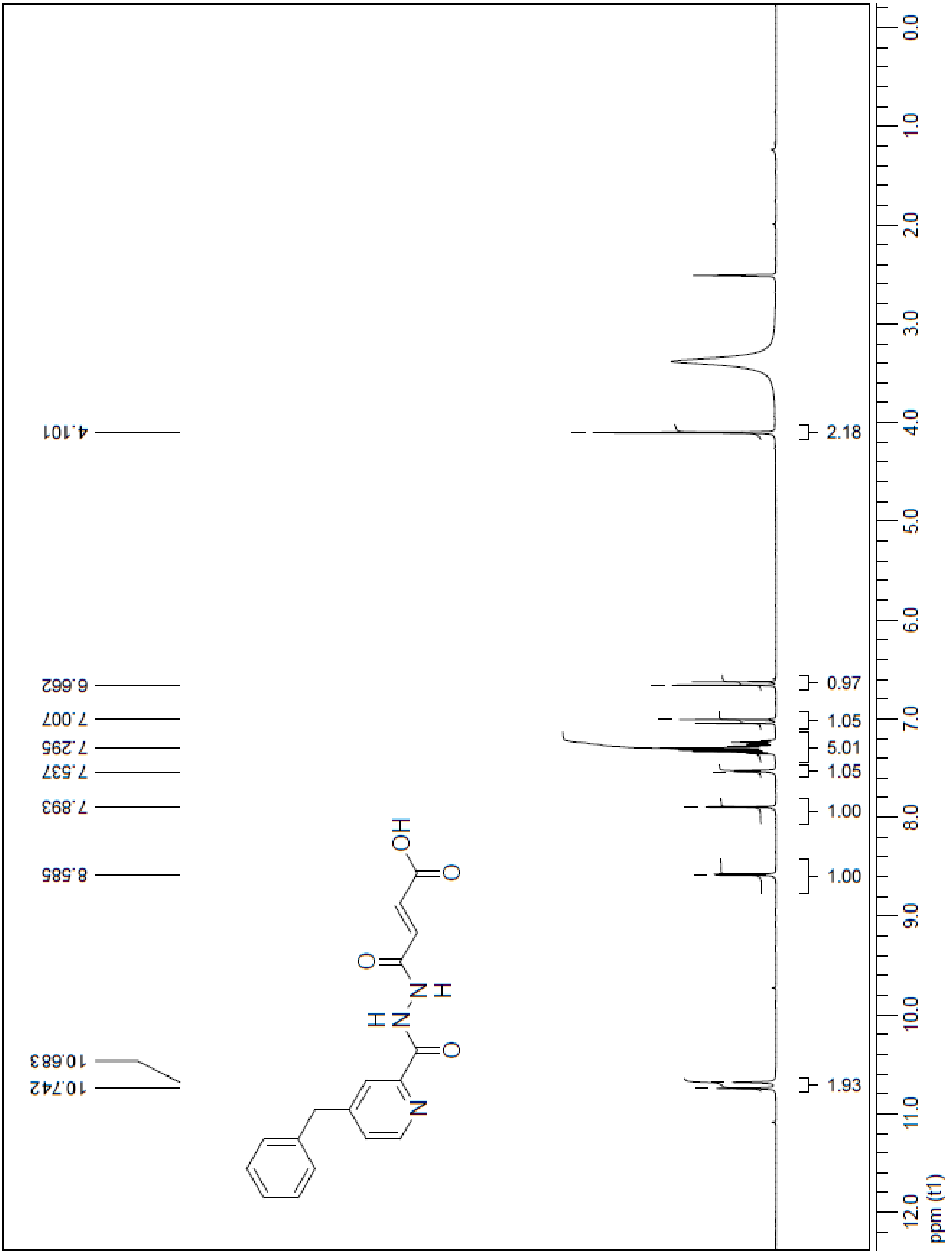

^{13}C NMR spectrum of 12

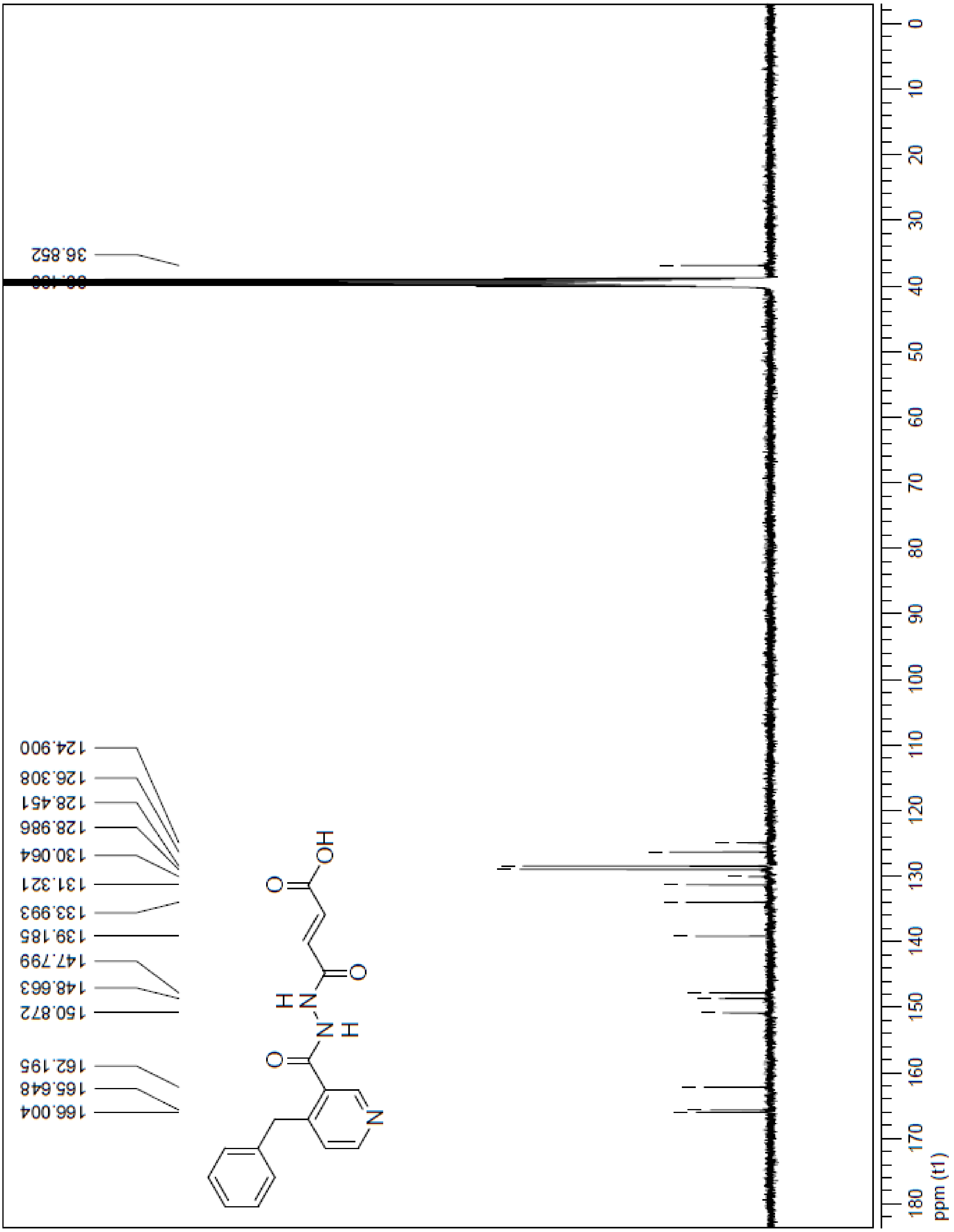

^{13}C NMR spectrum of 13

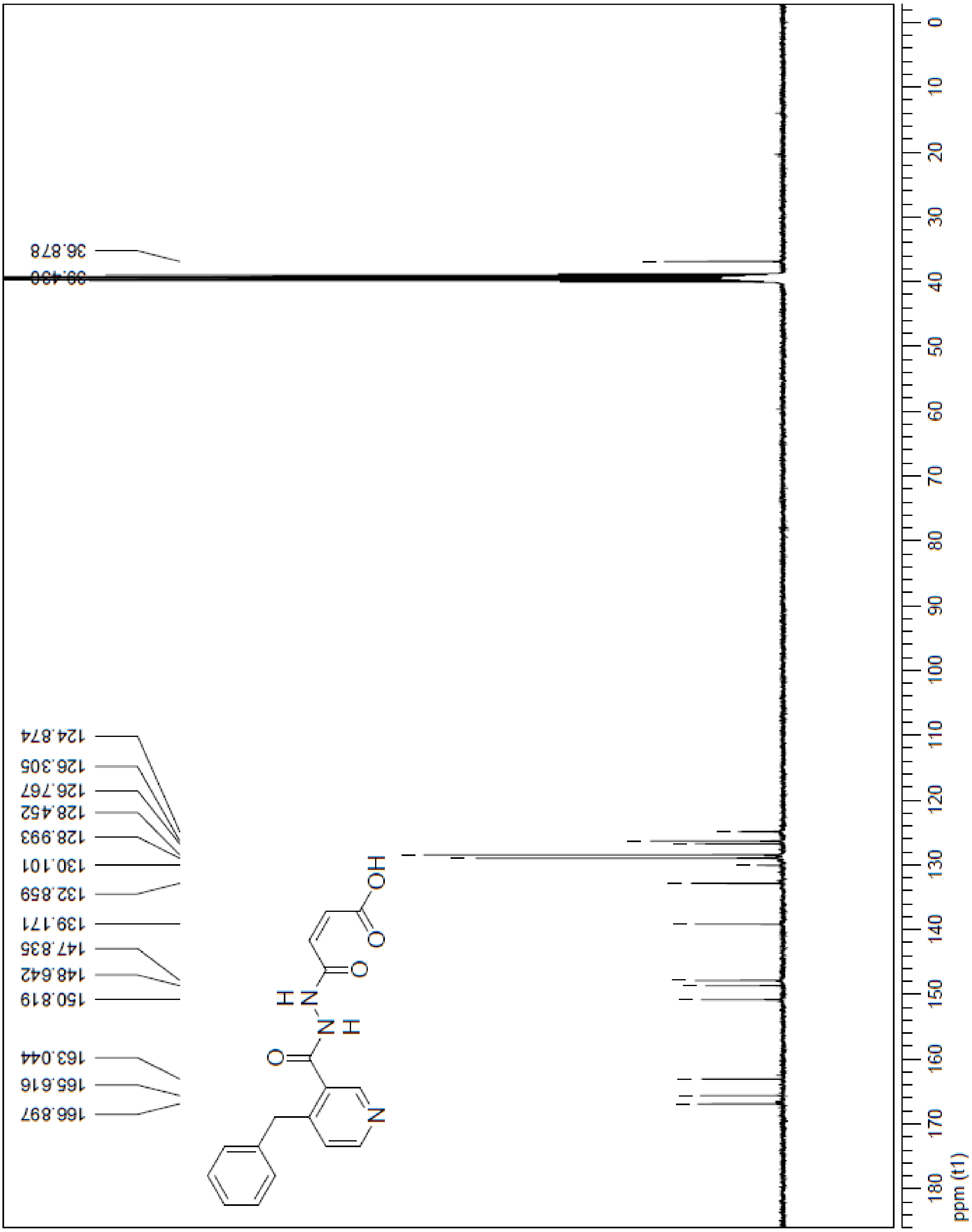

^{13}C NMR spectrum of 14

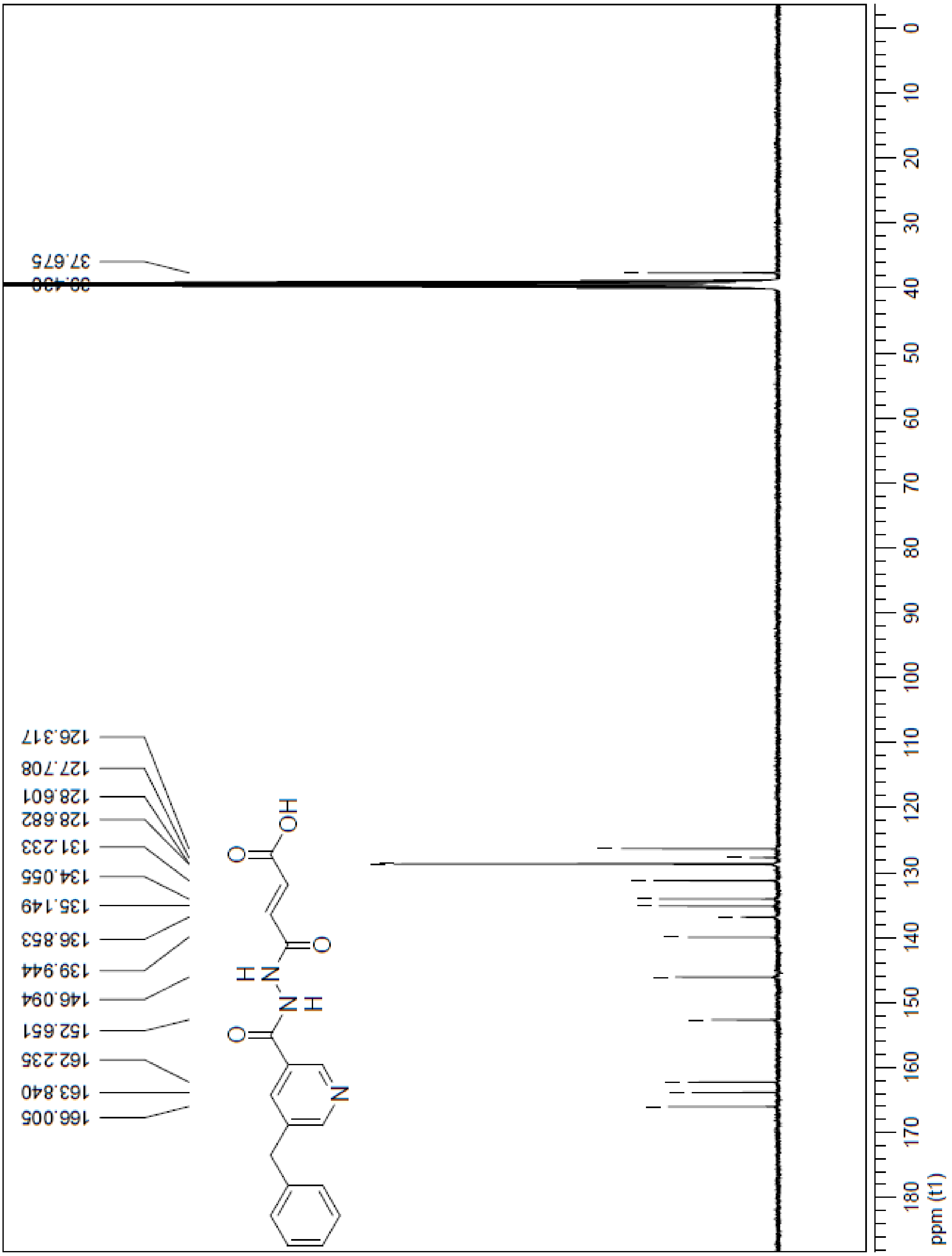

^{13}C NMR spectrum of 15

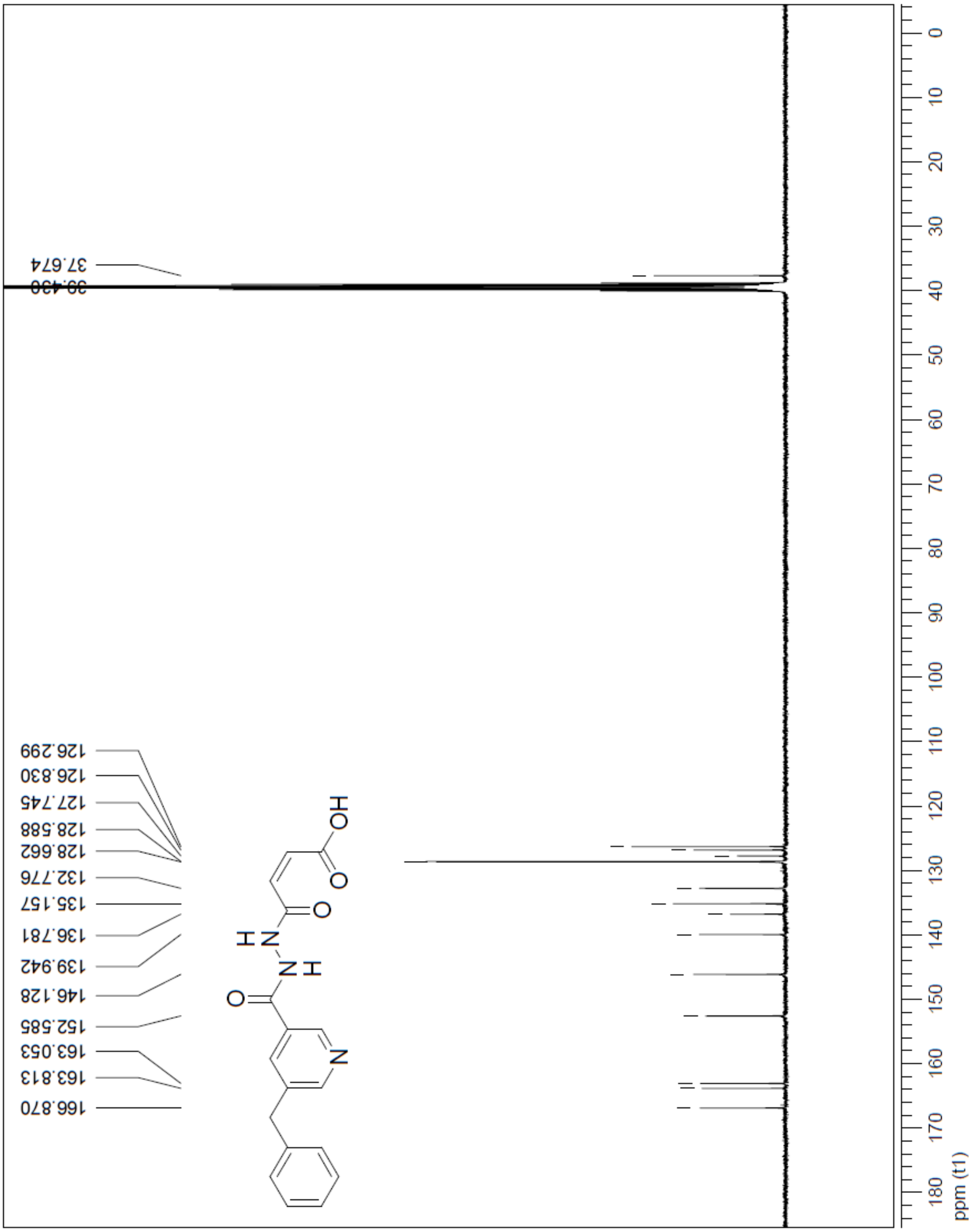

^{13}C NMR spectrum of 16

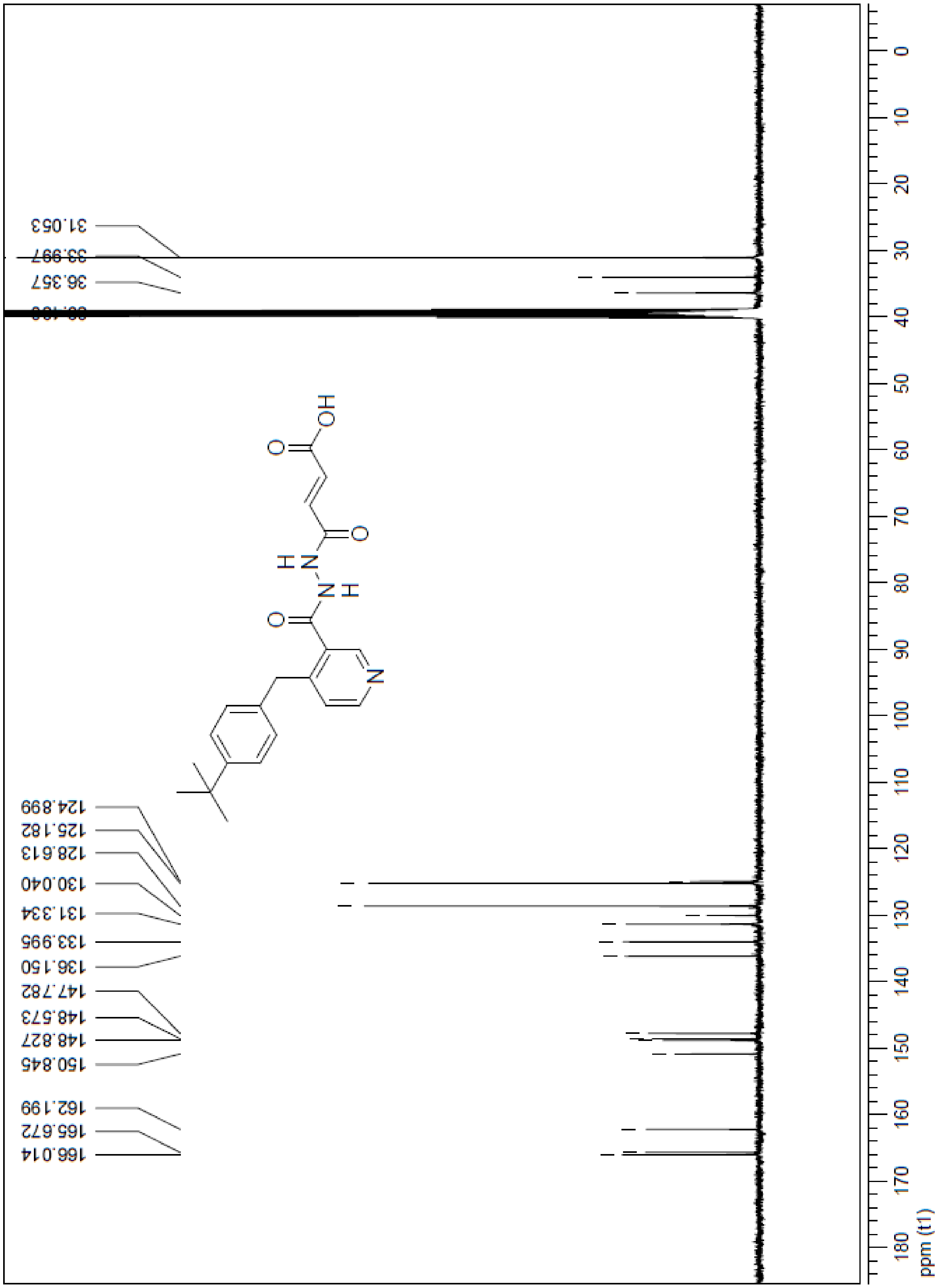

^{13}C NMR spectrum of 17

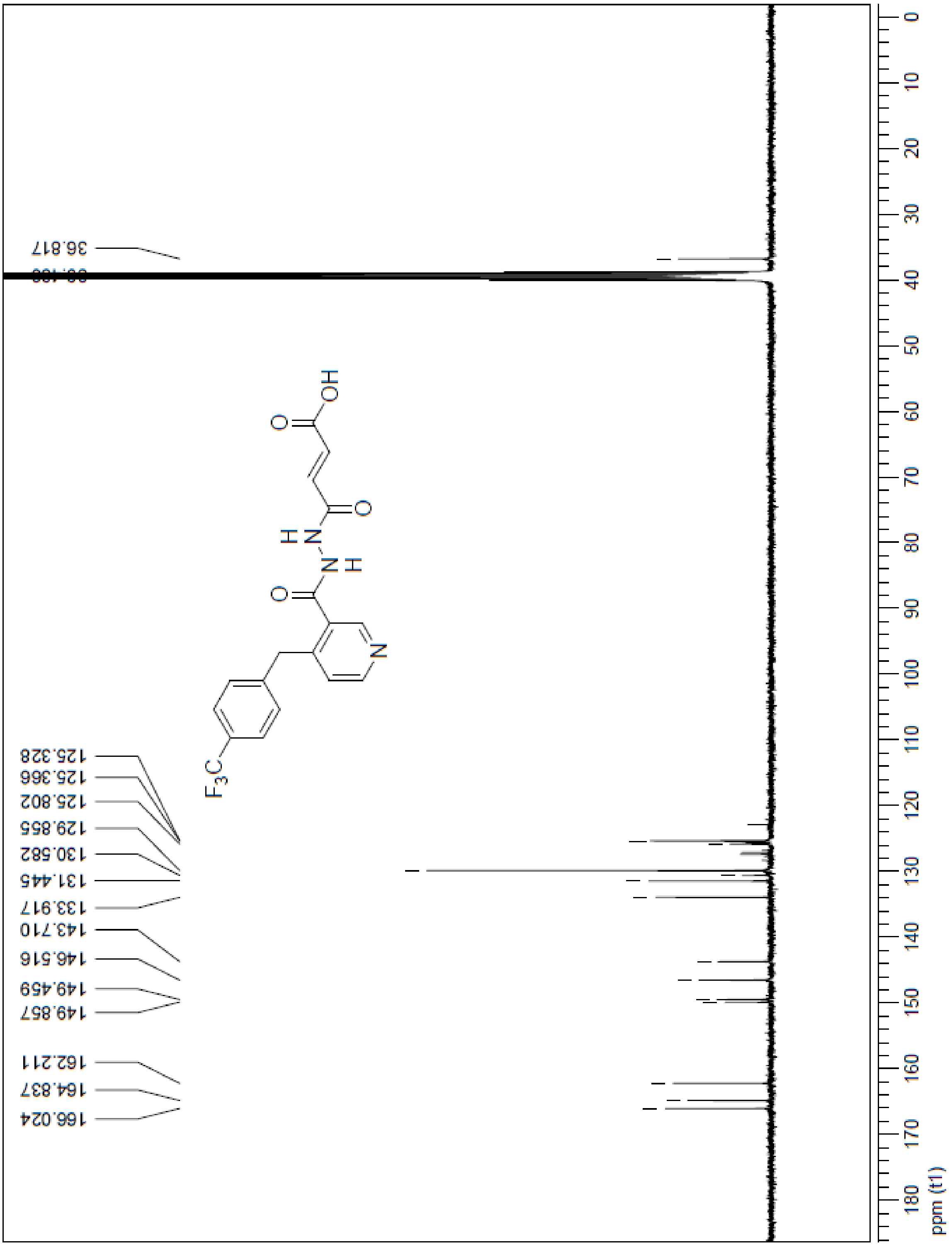

^{13}C NMR spectrum of 18

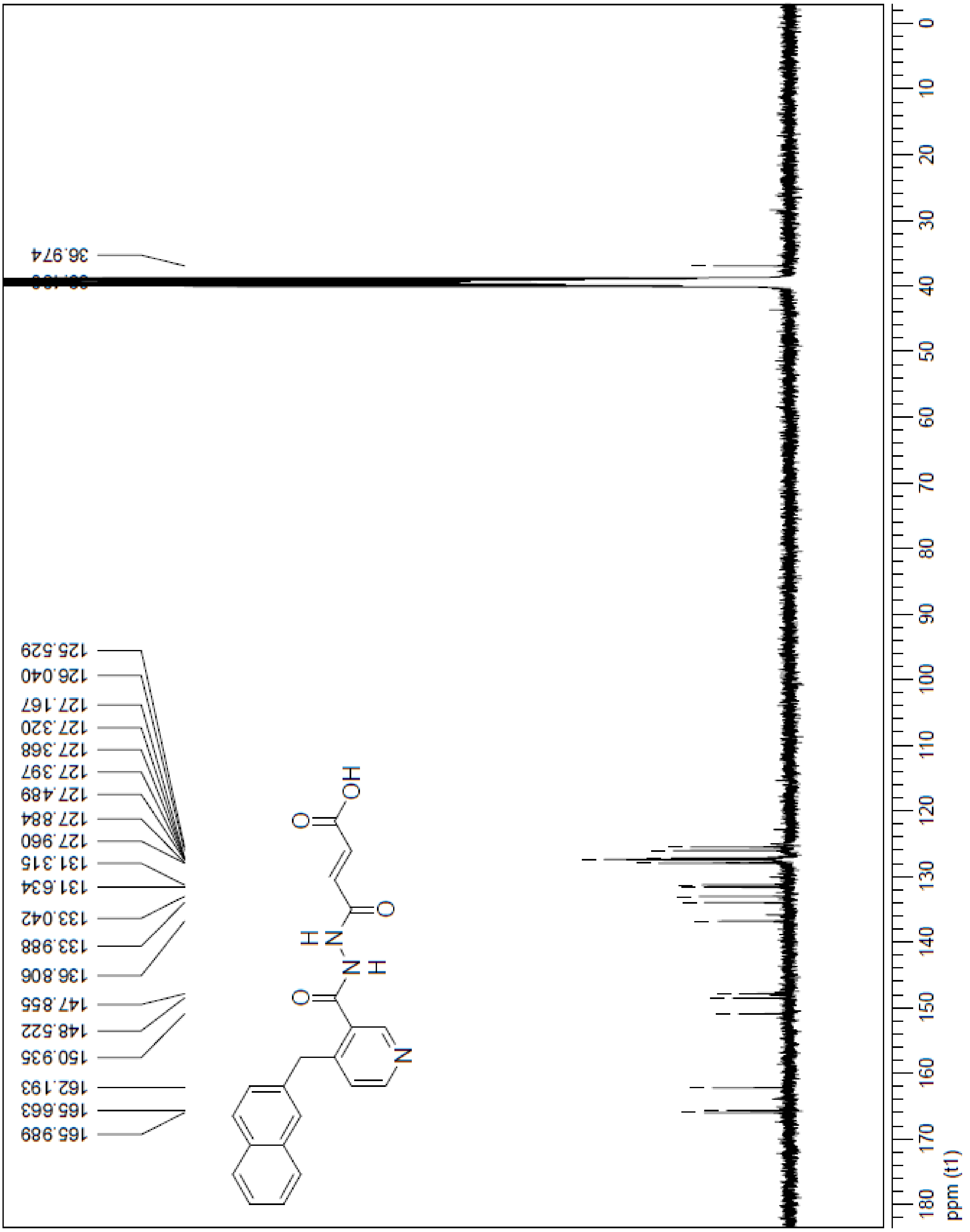

^{13}C NMR spectrum of 19

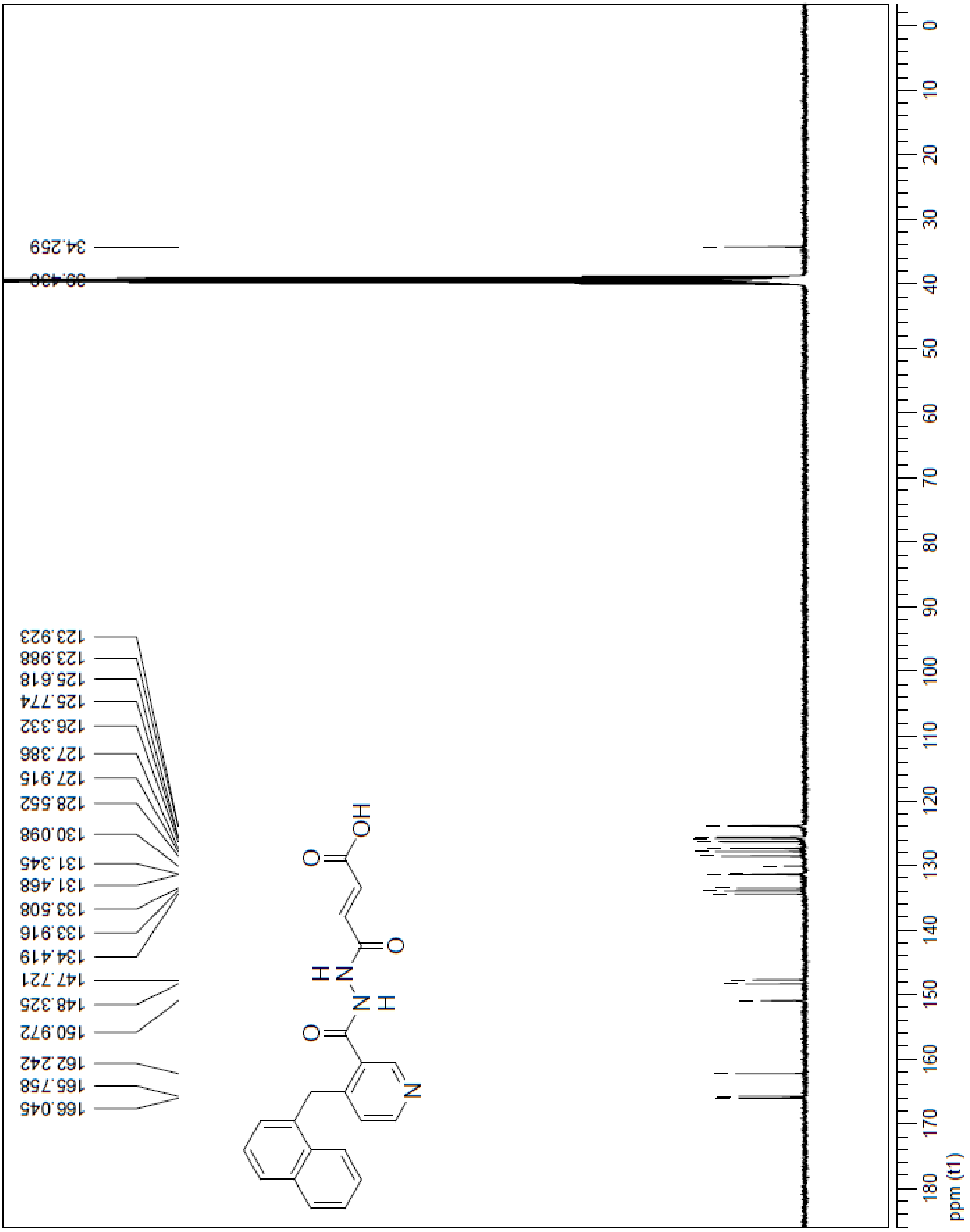

^{13}C NMR spectrum of 20

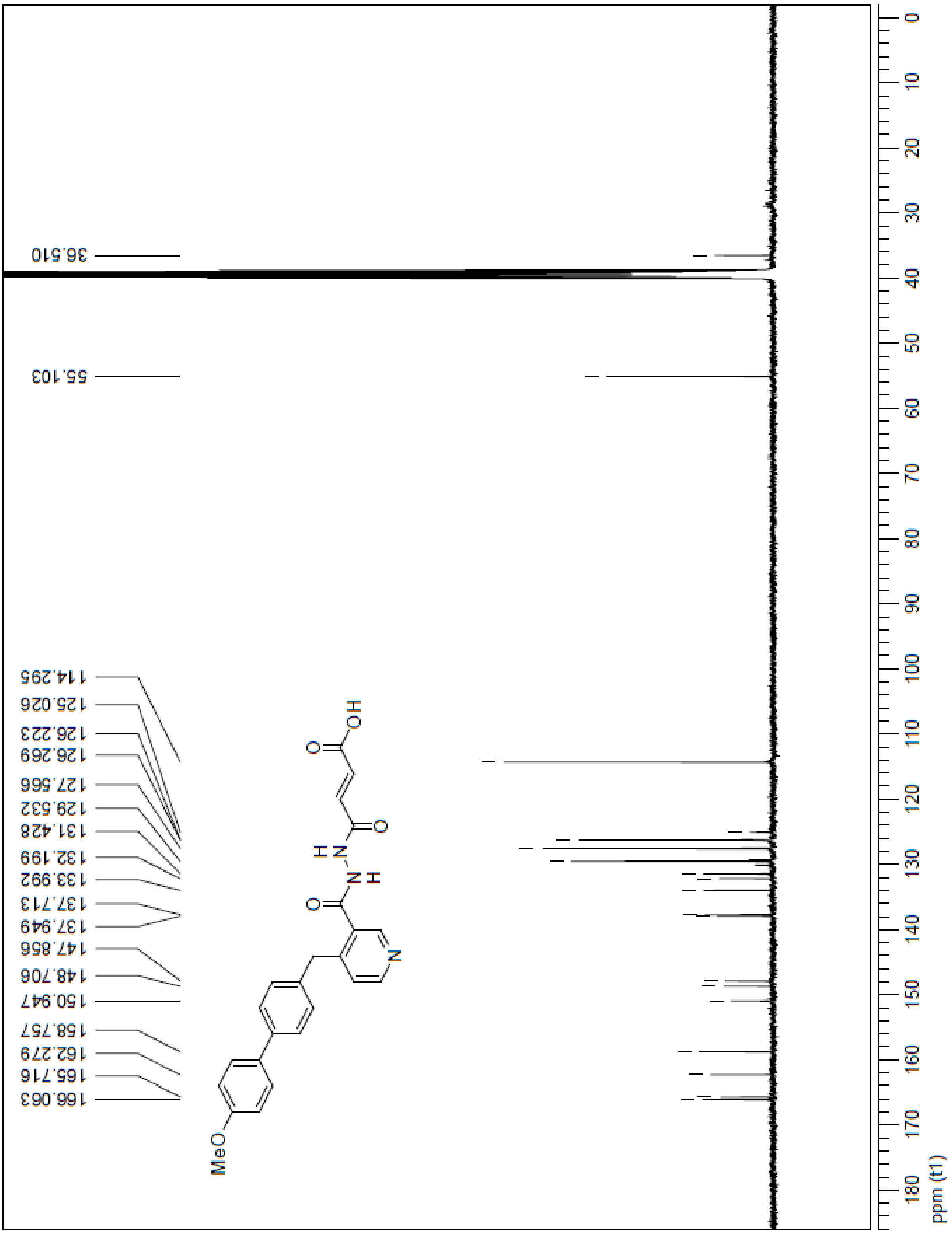

^{13}C NMR spectrum of 22

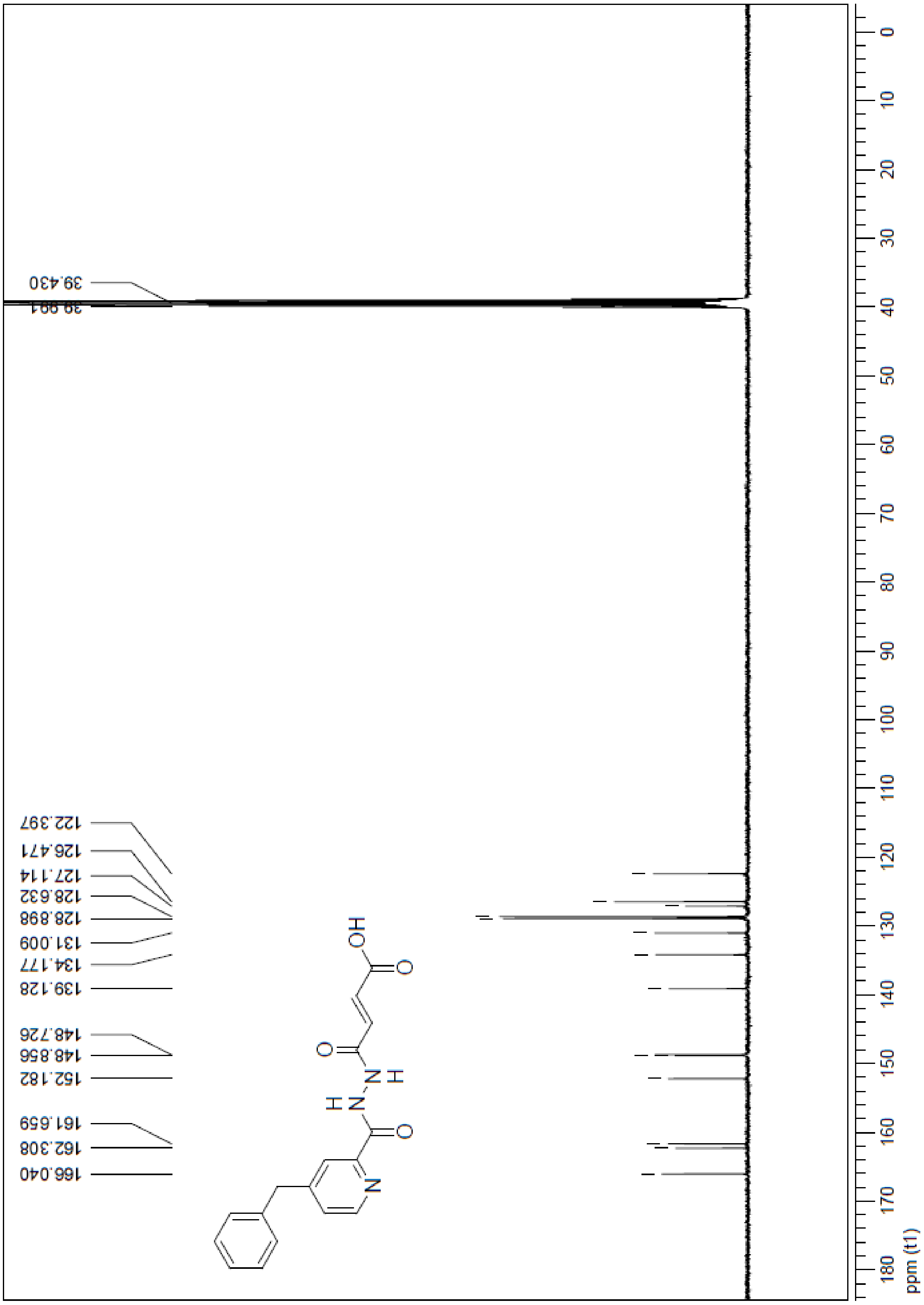
39.430
39.991
122.397
126.471
127.114
128.632
128.898
131.009
134.177
139.128
148.726
148.856
152.182
161.659
162.308
166.040
ppm (t1)

References

[1] Z. Han, T. Niu, J. Chang, X. Lei, M. Zhao, Q. Wang, W. Cheng, J. Wang, Y. Feng, J. Chai, *Nature* 2010, ***464***, 1205.

[2] B. Yu, J. F. Hunt, *Proc. Natl. Acad. Sci. U S A* 2009, ***106***, 14315.

[3] C. G. Yang, C. Yi, E. M. Duguid, C. T. Sullivan, X. Jian, P. A. Rice, C. He, *Nature* 2008, ***452***, 961.

[4] O. Sundheim, C. B. Vagbo, M. Bjoras, M. M. Sousa, V. Talstad, P. A. Aas, F. Drablos, H. E. Krokan, J. A. Tainer, G. Slupphaug, *EMBO J.* 2006, ***25***, 3389.

[5] W. Aik, J. S. Scotti, H. Choi, L. Gong, M. Demetriades, C. J. Schofield, M. A. McDonough, *Nucleic Acids Res.* 2014, ***42***, 4741.

[6] C. Zhu, C. Yi, *Angew. Chem. Int. Ed. Engl.* 2014, ***53***, 3659.

[7] C. Feng, Y. Liu, G. Wang, Z. Deng, Q. Zhang, W. Wu, Y. Tong, C. Cheng, Z. Chen, *J. Biol. Chem.* 2014, ***289***, 11571

[8] E. C. Y. Woon, M. Demetriades, E. A. Bagg, W. Aik, S. M. Krylova, J. H. Ma, M. Chan, L. J. Walport, D. W. Wegman, K. N. Dack, M. A. McDonough, S. N. Krylov, C. J. Schofield, *J. Med. Chem.* 2012, ***55***, 2173.

[9] P. Savitsky, J. Bray, C. D. Cooper, B. D. Marsden, P. Mahajan, N. A. Burgess-Brown, O. Gileadi, *J. Struct. Biol.* 2010, ***172***, 3.

[10] E. C. Y. Woon, A. Tumber, A. Kawamura, L. Hillringhaus, W. Ge, N. R. Rose, J. H. Ma, M. C. Chan, L. J. Walport, K. H. Che, S. S. Ng, B. D. Marsden, U. Oppermann, M. A. McDonough, C. J. Schofield, *Angew. Chem. Int. Ed. Engl.* 2012, ***51***, 1631.

[11] W. Kabsch, *J. Appl. Crystallogr.* 1993, **26,** 795.

[12] A. J. McCoy, R. W. Grosse-Kunstleve, P. D. Adams, M. D. Winn, L. C. Storoni, R. J. Read, *J. Appl. Crystallogr.* 2007, ***40***, 658.

[13] P. D. Adams, P. V. Afonine, G. Bunkoczi, V. B. Chen, I. W. Davis, N. Echols, J. J. Headd, L. W. Hung, G. J. Kapral, R. W. Grosse-Kunstleve, A. J. McCoy, N. W. Moriarty, R. Oeffner, R. J. Read, D. C. Richardson, J. S. Richardson, T. C. Terwilliger, P. H. Zwart, *Acta. Crystallogr. D Biol. Crystallogr.* 2010, ***66***, 213.

[14] W. Aik, M. Demetriades, M. K. Hamdan, E. A. Bagg, K. K. Yeoh, C. Lejeune, Z. Zhang, M. A. McDonough, C. J. Schofield, *J. Med. Chem.* 2013, ***56***, 3680.

[15] P. Emsley, B. Lohkamp, W. G. Scott, K. Cowtan, *Acta. Crystallogr. D Biol. Crystallogr.* 2010, ***66***, 486.

[16] F.H. Niesen, H. Berglund, M. Vedadi, *Nat. Protoc.* 2007, ***2***, 2212.

[17] Z. Han, T. Niu, J. Chang, X. Lei, M. Zhao, Q. Wang, W. Cheng, J. Wang, Y. Feng, J. Chai, *Nature* 2010, ***464*,** 1205.

[18] C. G. Yang, C. Yi, E. M. Duguid, C. T. Sullivan, X. Jian, P. A. Rice, C. He, *Nature* 2008, ***452***, 961.

[19] E. Flashman, E. A. Bagg, R. Chowdhury, J. Mecinovic, C. Loenarz, M. A. McDonough, K. S. Hewitson, C. J. Schofield, *J. Biol. Chem.* 2008, ***283***, 3808.

[20] N. R. Rose, E. C. Y. Woon, G. L. Kingham, O. N. King, J. Mecinovic, I. J. Clifton, S. S. Ng, J. Talib-Hardy, U. Oppermann, M. A. McDonough, C. J. Schofield, *J. Med. Chem.* 2010, ***53***, 1810.

[21] B. Chen, F. Ye, L. Yu, G. Jia, X. Huang, X. Zhang, S. Peng, K. Chen, M. Wang, S. Gong et al., *J. Am. Chem. Soc.* 2012, ***134***, 17963.

[22] K. K. Yeoh, M. C. Chan, A. Thalhammer, M. Demetriades, R. Chowdhury, Y. M. Tian, I. Stolze, L. A. McNeill, M. K. Lee, E. C. Y. Woon, M. M. Mackeen, A. Kawamura, P. J. Ratcliffe, J. Mecinovic, C. J. Schofield, *Org. Biomol. Chem.* 2013, ***11***, 732.

[23] Z. Moldovan, O. Garlea, T. Panea, A. Pop, D. Zinveliu, L. Bodochi, *Rapid Commun. Mass Spectrom* 1998, ***12***, 1313.

[24] N. V. Kolotova, E. N. Koz'minykh, V. É. Kolla, B. Y. Syropyatov, E. V. Voronina, V. O. Koz'minykh, *Pharm. Chem. J.* 1999, ***33***, 248.

[25] C. J. Cunliffe, T. J. Franklin, N. J. Hales, G. B. Hill, *J. Med. Chem.* 1992, ***35***, 2652.

[26] A. P. Krapcho, D. J. Waterhouse, A. Hammach, R. Di Domenico, E. Menta, A. Oliva, S. Spinelli, *Synth. Commun.*1997*, **27**,* 781.

Author contributions

Syntheses and characterizations of all compounds were carried out by Linyi Sun

Protein expression and crystallographic studies were carried out by Joel D. W. Toh, Jackie Tan and Yun Chen, under the supervision of Yong-Gui Gao and Wanjin Hong.

Activity assays were carried out by Lisa Z. M. Lau, Eleanor J. Y. Cheong and Melissa J. H. Tan.

Thermal shift assays and kinetic analyses were carried out by Joanne J. A. Low and Colin W. Q. Tang

The study was designed and the manuscript written by Esther C. Y. Woon, with assistance and comments from all other authors.
